# Immune dysregulation drives the relapse of peritoneal dialysis-associated peritonitis: a single-center prospective study

**DOI:** 10.3389/fimmu.2026.1810227

**Published:** 2026-07-03

**Authors:** Guang Yang, Xingbo Hong, Zhiwei Lai, Hua Zhang, Zuying Xiong, Zibo Xiong

**Affiliations:** 1 Division of Renal Medicine, Peking University Shenzhen Hospital, Peking University, Shenzhen, China; 2 Shenzhen Clinical Research Center for Urology and Nephrology, Shenzhen, China; 3 Renal Division, Peking University (PKU)-Shenzhen Clinical Institute of Shenzhen University Medical College, Shenzhen, China

**Keywords:** immune dysfunction, inflammation, peritoneal dialysis, peritonitis, relapsing

## Abstract

**Background:**

The diagnosis and management of relapsing peritoneal dialysis-associated peritonitis (PDAP) remains a clinical challenge. This study aimed to identify biomarkers associated with relapsing PDAP and discuss potential mechanisms.

**Methods:**

31 PDAP patients treated in 2023 were prospectively enrolled, including 23 cured patients and 8 with relapsing PDAP. Peritoneal dialysate samples were collected for conventional bacterial culture, 16S rDNA sequencing, and proteomic analysis.

**Results:**

The positivity rate for conventional culture was 64.5%, while that for 16S rDNA sequencing was 67.8%; combining both methods increased the detection rate to 83.9%. Microbiome analysis revealed that PDAP relapse may stem not only from exogenous pathogens but also from gut-derived bacterial translocation due to impaired local immunity. Proteomic profiling revealed that compared with the Cured group, the Relapse group showed downregulated levels of CCL28, CD40, and uPA, and upregulated NRTN. Bioinformatic analysis revealed dysregulation in pathways related to inflammation, fibrinolysis, and immune clearance, thus linking relapsing PDAP to a disturbed peritoneal immune microenvironment.

**Conclusion:**

Relapsing PDAP is linked to peritoneal immune dysregulation, and 16S rDNA sequencing represents a complementary diagnostic tool. These findings may guide precise management and improve patient outcomes.

## Highlights

- What was known: Relapsing peritoneal dialysis-associated peritonitis (PDAP) is clinically challenging, with unclear mechanisms beyond persistent infection. Conventional culture often fails to identify pathogens, and the role of host immune responses in relapse remains poorly understood.
- This study adds: Relapsing PDAP is linked to peritoneal immune dysregulation and gut bacterial translocation. A biomarker panel (CCL28, CD40, uPA, NRTN) distinguishes relapse from cure. 16S rDNA sequencing complements culture, raising detection rates to 83.9%.
- Potential impact: These findings support a shift toward immune-microenvironment-targeted management and precision diagnostics in PDAP, potentially improving relapse prediction, pathogen identification, and therapeutic strategies to preserve peritoneal membrane integrity and patient outcomes.

## 1 Introduction

Chronic kidney disease (CKD) frequently progresses to end-stage renal disease, necessitating renal replacement therapies such as peritoneal dialysis (PD) (1, 2). PD-associated peritonitis (PDAP) remains a major complication, with relapsing episodes posing a particularly difficult clinical challenge (3, 4). Relapsing PDAP, defined as a recurrent episode within four weeks of completing prior therapy, often leads to prolonged antibiotic use, catheter removal, and poor patient outcomes (4, 5). Accurate identification of relapse and understanding its underlying mechanisms are critical for improving management. Current diagnostic approaches, primarily reliant on conventional bacterial culture, frequently yield negative or delayed results, failing to guide timely and targeted therapy. Moreover, the pathogenesis of relapse extends beyond persistent infection (6); factors such as biofilm formation, bacterial translocation, residual microbial DNA fragments and immune dysregulation are implicated but poorly understood (7–9). This knowledge gap hinders the development of prognostic tools and mechanism-based interventions.

To address these limitations, we designed a prospective study integrating multi-omics profiling of peritoneal dialysis effluent (PDE). We combined 16S rDNA sequencing—to enhance pathogen detection—with high-throughput proteomics, aiming to uncover host-derived molecular signatures associated with relapse. We hypothesized that relapsing PDAP arises not only from microbial persistence but also from a dysregulated peritoneal immune microenvironment, potentially linked to gut-derived bacterial translocation. Our objectives were twofold: first, to evaluate the complementary diagnostic value of 16S rDNA sequencing alongside conventional culture; and second, to identify novel protein biomarkers and pathway alterations that distinguish relapsing from cured PDAP. These findings are expected to provide insights for the prevention and targeted intervention of relapsing PDAP.

## 2 Methods

### 2.1 Ethics

**Figure 1:**
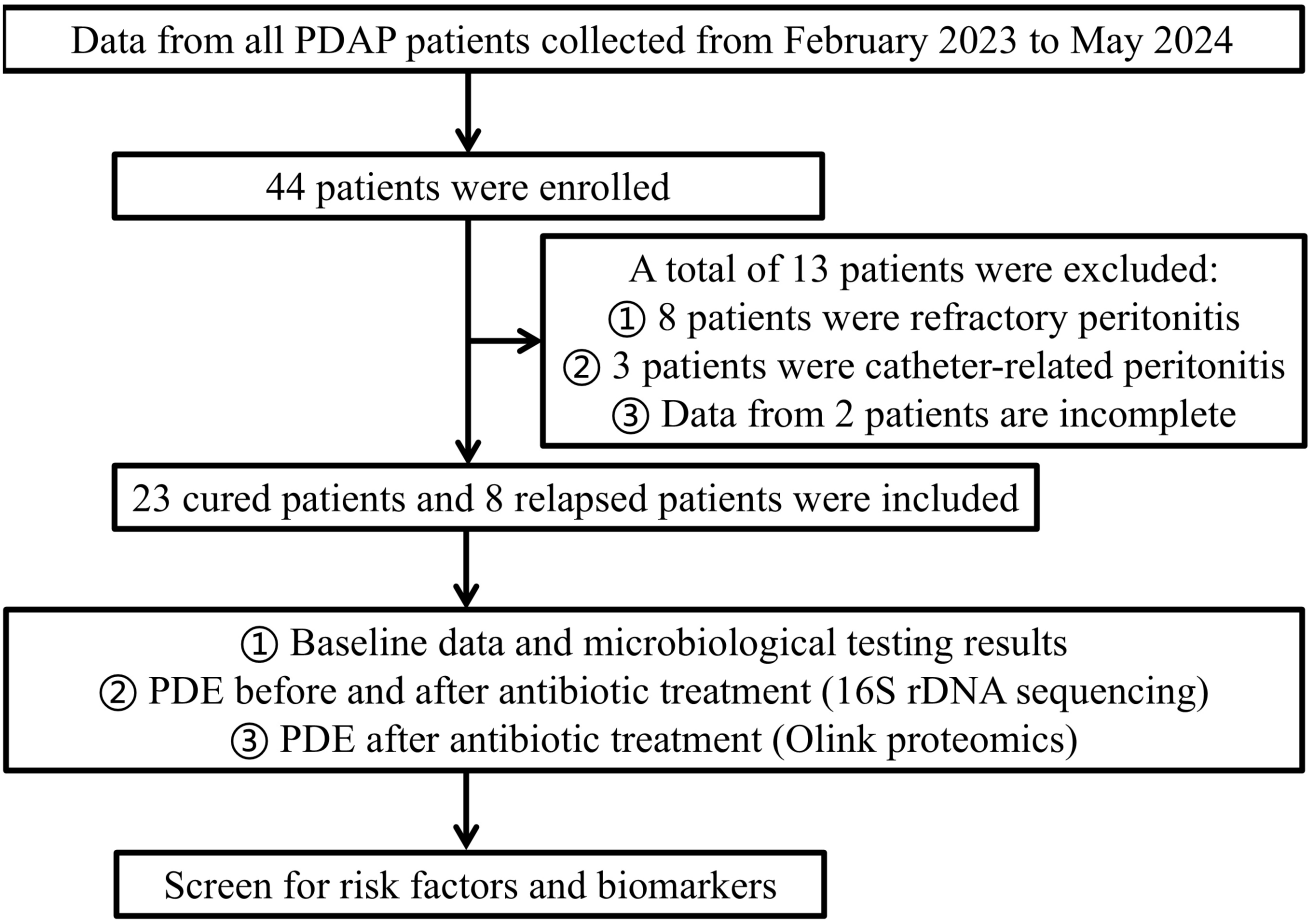
Research workflow.

This study is a prospective investigation conducted in accordance with the Declaration of Helsinki. All study procedures were approved by the Ethics Committee of Peking University Shenzhen Hospital (No. 2022050). National Clinical Trial Registration Number is ChiCTR2200064540. Written informed consent was obtained from all patients at the time of treatment. The diagnostic and therapeutic procedures strictly adhered to the International Society for Peritoneal Dialysis (ISPD) guidelines (4). No additional interventions or burdens were imposed on patients during the study, and their clinical management plans were not altered for research purposes (**Figure 1**). Ultimately, data from 31 individuals were included in the study. Biochemical testing for all patients was completed in the Clinical Laboratory of our hospital.

### 2.2 Selection criteria

Inclusion criteria: ① Long-term outpatient/inpatient visits at the department; ② Age 18–75 years; ③ Diagnosis of recurrent PDAP and receipt of treatment at our hospital. Exclusion criteria:​ ① Presence of severe genetic disease; ② Presence of psychiatric illness; ③ Concurrent diagnosis of infection at other sites or viral infection during treatment.

### 2.3 Culture

Diagnosis followed ISPD guidelines (4). Upon suspicion of peritonitis, the first cloudy effluent was submitted for cell count with differential analysis. Microbiological culture was performed in aerobic, anaerobic, and antibiotic-neutralizer blood culture bottles using centrifuged (or, if not possible, sedimented) samples.

### 2.4 16S rDNA

PDE samples were shipped on dry ice to Lianchuan-Bio (Hangzhou, China), a third-party sequencing service provider, for 5R 16S sequencing, i.e., multiplex PCR amplification and sequencing of five hypervariable regions of the 16S rDNA gene. Subsequently, bacterial taxonomic classification was performed using the Short MUltiple Regions Framework (SMURF), which integrates sequence data from all five amplified regions. This approach enabled within-group and between-group analyses of microbial diversity as well as identification of differentially abundant taxa. LEfSe (Linear Discriminant Analysis Effect Size) is a method used to identify microbial biomarkers that exhibit both statistical significance and biological relevance between different groups. The higher the LDA Score (log_10_), the greater the enrichment and representativeness of that taxon in a specific group.

### 2.5 Proteomics

After centrifugation, samples were aliquoted and stored at −80 °C, then shipped on dry ice to Lianchuan-Bio (Hangzhou, China). The Olink Proteomics platform (based on Proximity Extension Assay technology) was used for high-throughput quantitative profiling of protein biomarkers in PDE samples. Prior to analysis, all samples were thawed uniformly and processed according to the standard operating procedures provided by Olink, including dilution and incubation steps. Each assay plate included internal controls, negative controls, and calibrators to correct for inter-plate variability. Raw data were reported as Normalized Protein eXpression values, a log2-scale relative quantification unit.

### 2.6 Statistics

Results are expressed as mean ± standard deviation. For proteomic data analysis (n=6 per group), differences between groups were assessed using unpaired t-test, and statistical significance was determined using the Benjamini-Hochberg (BH) procedure for false discovery rate (FDR) correction, with a q-value ≤ 0.05 considered statistically significant. For non-proteomic analyses, a *p*-value ≤0.05 was considered statistically significant. Special algorithms are described in separate annotations.

## 3 Results

### 3.1 Patient characteristics

After rigorous screening, we selected 31 PDAP patients, including 23 who experienced a single episode and were subsequently cured, and 8 with relapsing PDAP. First, differences in baseline characteristics before antibiotic therapy were compared between patients in the Cured group and the Relapse group (**Table 1**). The results of the serum biochemical parameters indicated no statistically significant differences between the two groups in continuous variables, including age, dialysis vintage, white blood cell count, neutrophil count, hemoglobin, blood urea nitrogen, creatinine, potassium, calcium, log_2_−transformed procalcitonin, and log_2_−transformed interleukin−6. In terms of gender distribution, males accounted for 63% and 74% of the Cured and Relapse groups, respectively. The Chi-square test showed no statistically significant difference in gender distribution between the Cured group (63% male) and the Relapse group (74% male) (χ 2 χ 2 = 0.58, *p* = 0.446). This indicates that the gender composition is well balanced between the two groups, and the confounding effect of gender on the study outcomes is likely minimal. Overall, the baseline characteristics of the two groups were well balanced and demonstrated good comparability.

**Table 1:** Comparison of baseline data between the two groups before antibiotic therapy.

General information	Cured (n=23)	Relapse (n=8)	p
Age (years)	51.57 ± 13.27	54.38 ± 11.78	0.6005
Gender (male %)	63	74	
Dialysis vintage (months)	66.39 ± 57.21	40.13 ± 39.76	0.2415
WBC (E + 9/L)	8.673 ± 4.261	7 ± 3.527	0.3279
Neutrophil (E + 9/L)	6.601 ± 4.021	5.441 ± 3.099	0.4642
Hemoglobin (g/L)	95.62 ± 25.89	105.3 ± 17.75	0.3401
BUN (mmol/L)	16.99 ± 5.875	17.12 ± 3.538	0.9536
Creatinine (μmol/L)	893.4 ± 302	919.3 ± 281.3	0.8341
Potassium (mmol/L)	3.667 ± 0.4782	3.91 ± 0.9686	0.3577
Calcium (mmol/L)	2.128 ± 0.1667	2.073 ± 0.1407	0.408
Procalcitonin (ng/mL, log2)	0.7186 ± 2.796	1.796 ± 3.659	0.3932
Interleukin-6 (pg/mL, log2)	6.594 ± 3.399	6.287 ± 2.727	0.8199

WBC, white blood cell; BUN, blood urea nitrogen. Procalcitonin and interleukin-6 were log2-transformed before performing the t-test.

### 3.2 Diagnosis and treatment

Next, microbiological testing results (**Table 2**) showed that 20 patients (64.5%) had positive conventional bacterial cultures. Among the positive specimens, 13 cases (65.0%) were infected with Gram-positive cocci, and 7 cases (35.0%) with Gram-negative bacilli. The distribution of identified pathogens was as follows: *Staphylococcus aureus* (n = 3), *Escherichia coli* (n = 2), *Streptococcus salivarius* (n = 5), *Pseudomonas aeruginosa* (n = 4), *Staphylococcus epidermidis* (n = 5), *Burkholderia cepacia* (n = 1), and 11 cases with no identifiable pathogen (N/A). The positive detection rate was 64.5%, which does not meet the ISPD requirement of 80-85%.

**Table 2:** Patients information and grouping.

Case	Pathogen	Gram	Cured or relapsed	Number of episodes	Proteomics group
1	*Staphylococcus aureus*	+	C	1	
2	*Escherichia coli*	–	C	1	C
3	*Streptococcus salivarius*	+	C	2	
4	*Pseudomonas aeruginosa*	–	R	2	R
5	N/A		R	2	R
6	*Staphylococcus epidermidis*	+	C	2	
7	*Staphylococcus epidermidis*	+	R	4	R
8	*Streptococcus salivarius*	+	R	4	R
9	N/A		C	2	C
10	N/A		R	3	R
11	*Streptococcus salivarius*	+	C	2	
12	*Burkholderia cepacia*	–	R	3	R
13	*Pseudomonas aeruginosa*	–	R	2	R
14	*Streptococcus salivarius*	+	C	2	
15	*Staphylococcus aureus*	+	C	1	C
16	*Pseudomonas aeruginosa*	–	R	4	R
17	*Staphylococcus epidermidis*	+	C	1	
18	N/A		C	1	C
19	N/A		C	1	C
20	*Staphylococcus epidermidis*	+	C	1	C
21	N/A		C	1	C
22	N/A		C	1	
23	*Streptococcus salivarius*	+	C	1	
24	*Staphylococcus aureus*	+	C	1	
25	N/A		C	1	
26	*Staphylococcus epidermidis*	+	C	1	
27	N/A		C	1	
28	*Pseudomonas aeruginosa*	–	C	1	C
29	*Escherichia coli*	–	C	1	
30	N/A		C	1	
31	N/A		C	1	

C, cured; R, relapsed; N/A, culture-negative.

Clinical outcomes showed that, after standard anti-infective therapy (2–3 weeks), 23 patients were cured, yet 8 patients (25.8%) experienced relapse within one month. The number of peritonitis episodes ranged from 1 to 4. Relapsing cases were primarily associated with infections caused by *Pseudomonas aeruginosa*, *Staphylococcus epidermidis*, *Streptococcus salivarius*, or unknown pathogens. Overall, none of these results clearly indicate a correlation with relapsing PDAP, and further exploration is still needed.

### 3.3 16S rDNA sequencing increases the detection rate of unknown pathogens

**Figure 2:**
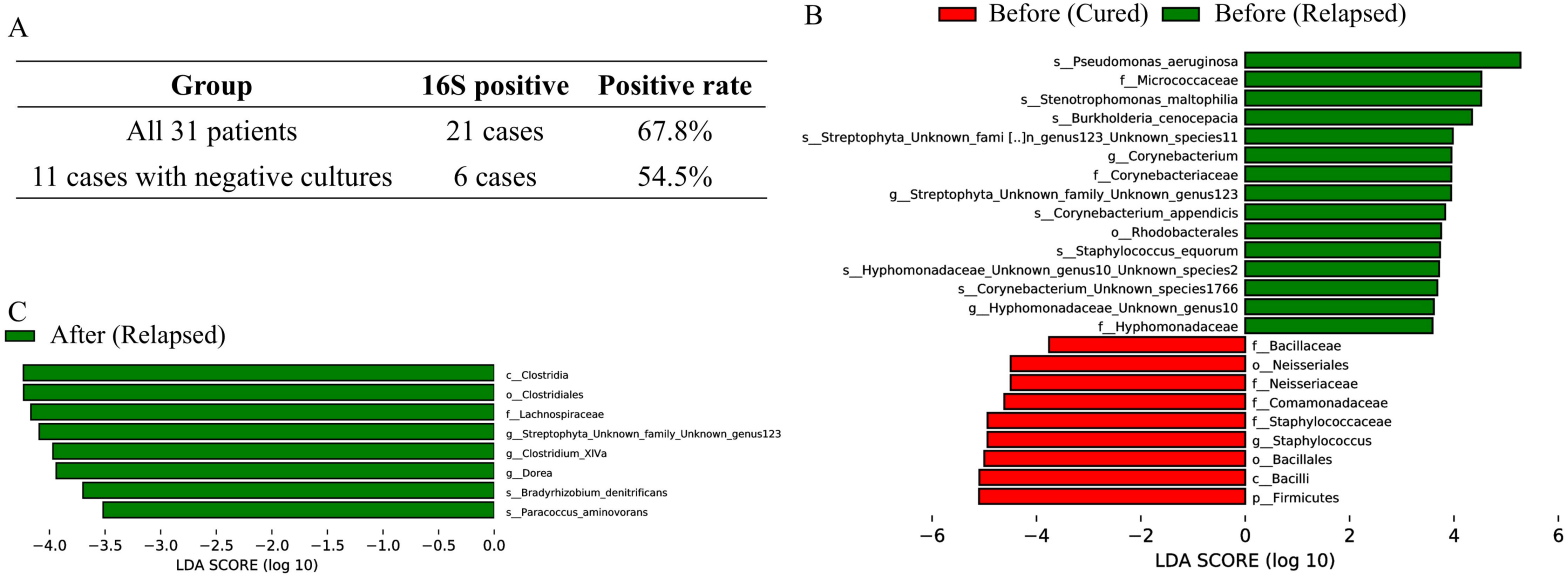
16S rDNA sequencing can be used to trace infection sources. **(A)** 16S rDNA sequencing increases the detection rate of pathogens. **(B, C)** LEfSe was applied to analyze the peritoneal microecology before and after treatment.

Subsequently, we validated whether 16S rDNA sequencing can be used to trace infection sources and discover new biomarkers. Based on pre-treatment PDE samples, 16S rDNA sequencing yielded an overall positive rate of 67.8% (21 cases), including identifying 6 positive cases among the 11 patients with negative cultures (**Figure 2A**). While this demonstrates that 16S rDNA sequencing can serve as a supplementary method when bacterial cultures yield negative results, it also indicates that it does not fully meet the clinical need for higher positive detection rates.

Moreover, the composition and relative abundance of the peritoneal microbial community changed significantly before and after treatment (**Supplementary Figure 1**). The pre-treatment results revealed that (**Figure 2B**), compared to cured patients, relapsing patients already harbored a more aggressive and antibiotic-resistant pathogenic microbial community in their dialysate prior to therapy. In contrast, cured patients were enriched with low-virulence or environmentally associated microbes. This suggests that baseline microbial profiles may serve as prognostic biomarkers to guide personalized treatment strategies. The post-treatment results (**Figure 2C**) indicate that all bars in the figure are positive (green), suggesting that the listed microorganisms were significantly enriched in the Relapse group after treatment. In contrast, no specific enrichment of microbes was observed in the Cured group post-treatment (or the enrichment did not reach the LEfSe threshold). This may indicate an extremely low microbial load or near-sterile condition in their dialysate. This also reflects effective control of inflammation, absence of persistent bacterial colonization, or translocation of enteric bacteria. Overall, these findings suggest that infections in Relapse group may arise not only from exogenous pathogens but also from endogenous translocation of gut-derived bacteria. Even after antibiotic treatment, their peritoneal cavity still harbors or becomes re-colonized by anaerobic gut microbiota, indicating underlying intestinal barrier dysfunction or insufficient local immune clearance. The underlying reasons for these phenomena warrant further investigation.

### 3.4 Relapsing PDAP is associated with aberrant expression of CCL28, CD40, uPA, and NRTN

**Figure 3:**
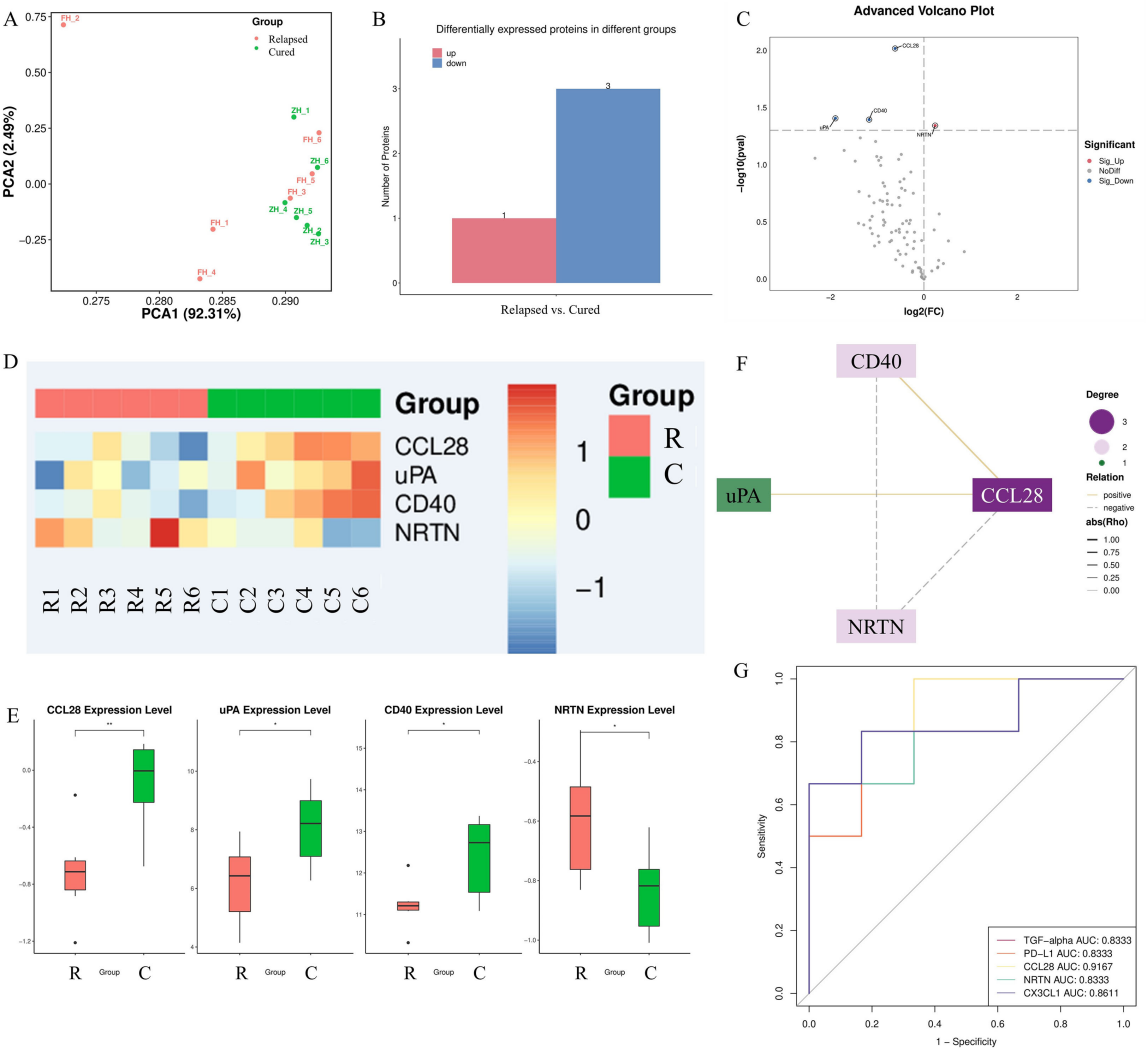
The protein composition of PDE differs between patients with relapsing PDAP and those who were cured. **(A)** PCA graph. **(B)** After consistency filtering, the Relapse group exhibited three downregulated proteins and one upregulated protein. **(C)** A volcano plot highlights these four proteins as showing the greatest differential expression between the two groups. **(D, E)** Heatmap and bar plots illustrate the magnitude and statistical significance of these differences. **(F)** Interrelationships among the four proteins. CCL28 may serve as a central regulatory factor, with its expression level positively correlated with CD40 and uPA, and negatively correlated with NRTN. **(G)** ROC curve analysis identified five key proteins with significant diagnostic/prognostic value.

To investigate whether relapsing PDAP is associated with the peritoneal immune microenvironment and to identify novel biomarkers, we selected PDE samples from 16 patients (8 who were cured and 8 who experienced relapse) and applied proteomics to screen for specific biomarkers associated with PDAP relapse. After data cleaning, 6 samples per group remained for analysis. Principal component analysis (PCA) (**Figure 3A**) revealed that samples from the Cured group clustered tightly together, whereas some relapsing PDAP samples were markedly distant from this cluster, indicating substantial differences in protein composition. We then performed differential protein expression analysis. Following consistency filtering, we identified three proteins significantly downregulated in the Relapse group (CCL28, CD40, and uPA; q<0.05) and one protein (NRTN, neurturin, a neurotrophic factor) significantly upregulated (q<0.05) (**Figures 3B–E**). Subsequent bioinformatic analysis suggested that CCL28 may serve as a central regulatory node among these four proteins (**Figure 3F**). Its expression was positively correlated with CD40 and uPA, and negatively correlated with NRTN. Furthermore, we evaluated the diagnostic/prognostic potential of individual proteins using receiver operating characteristic (ROC) curve analysis (**Figure 3G**). This identified five proteins with strong discriminatory power (all q<0.05 after FDR correction): CCL28 (AUC = 0.9167), CX3CL1 (0.8611), TGF-alpha (0.8333), PD-L1 (0.8333), and NRTN (0.8333). Based on these findings, we propose that the combination of CCL28 and NRTN may represent an optimal biomarker panel for predicting relapsing PDAP.

### 3.5 Relapsing PDAP is associated with immune microenvironment

**Figure 4:**
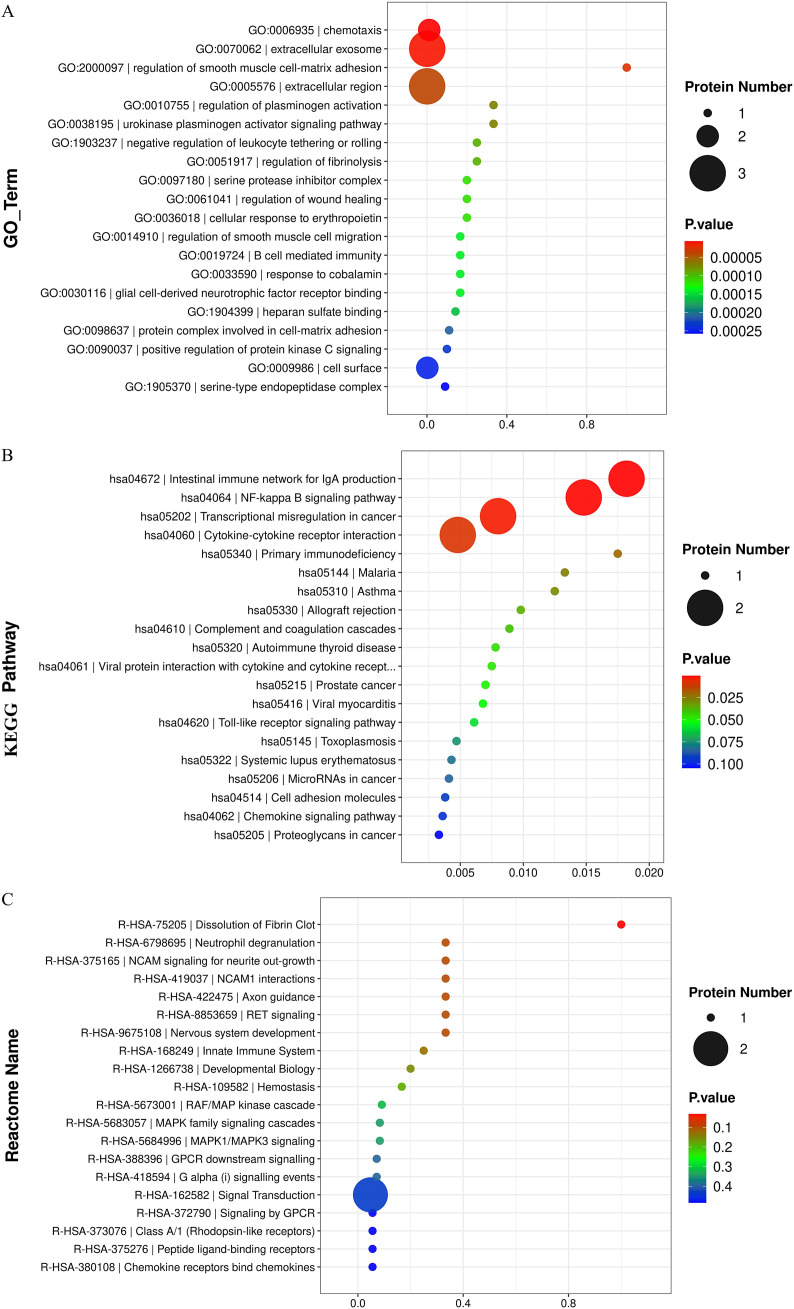
Bioinformatic analyses of the potential mechanisms underlying PDE compositional differences. **(A)** GO, **(B)** KEGG, and **(C)** Reactome. All converge on immune-fibrinolytic-repair dysregulation as a central driver of PDAP relapse.

Both CCL28 and CD40 are involved in immune regulation, suggesting that alterations in the peritoneal immune microenvironment may serve as a regulatory mechanism for PDAP relapse. To further validate this hypothesis, we conducted additional bioinformatics analyses on our results. GO (Gene Ontology) enrichment analysis indicated that compared to Cured group, the differentially expressed proteins in Relapse group were significantly enriched in pathways related to dysregulation of the fibrinolytic/protease system, potentially leading to impaired fibrin clearance (**Figure 4A**). Additionally, there was abnormal extracellular matrix remodeling and altered cell adhesion, which could promote fibrosis and bacterial retention. Furthermore, there was an imbalance in immune regulation (involving B cells and leukocyte adhesion), along with compromised tissue repair capacity. These findings collectively point to a core pathological mechanism: in relapsing PDAP patients, there is a dysfunction in the “inflammation-fibrinolysis-repair” axis, resulting in persistent infection, ongoing damage to peritoneal structure, and ultimately relapse.

KEGG (Kyoto Encyclopedia of Genes and Genomes) enrichment analysis revealed that the protein profile of Relapse group is characterized by (**Figure 4B**): sustained activation of pro-inflammatory pathways (such as NF-κB and cytokine signaling); imbalance in the complement-coagulation-fibrinolysis system, promoting fibrin deposition; inadequate expression of key immune effector molecules, akin to a “functional immunodeficiency”; and potential involvement of aberrant adaptive or autoimmune components that interfere with normal healing processes.

Reactome pathway analysis showed that compared to the Cured group, the proteins in Relapse group were significantly enriched in biological processes such as inflammatory responses (particularly chemokine/GPCR/MAPK pathways), neutrophil activation, and coagulation-fibrinolysis imbalance (**Figure 4C**). Relapsing PDAP patients may experience persistent or dysregulated innate immune activation; impaired fibrin clearance could facilitate pathogen persistence or worsen the local microenvironment; and abnormalities in neuroimmune-related pathways might reflect failed peritoneal tissue repair or immune homeostasis restoration.

Additionally, both DO (Disease Ontology) and InterPro analyses yielded similar results (**Supplementary Figure 2**). Overall, the consistent results from GO, KEGG, and Reactome analyses collectively indicate a tripartite mechanism of relapse involving “uncontrolled inflammation + fibrinolysis impairment + insufficient immune clearance.” This suggests that an imbalance in the peritoneal immune microenvironment may underlie PDAP relapse.

## 4 Discussion

Currently, the clinical challenges in relapsing PDAP lie in pathogen identification and understanding its underlying mechanisms. This study conducted preliminary explorations to address these issues. The first key finding is that neither bacterial culture nor 16S rDNA sequencing alone can fully identify the pathogens, yet when used in combination, they complement each other and increase the detection rate to 83.9%. Moreover, through multi-omics analysis of PDE from patients with relapsing versus cured PDAP, this study preliminary uncovers a complex host-microbe interplay underlying disease relapse. Post-treatment enrichment of gut-derived anaerobic commensals in the PDE strongly implicates intestinal barrier dysfunction and bacterial translocation as key endogenous triggers of PDAP relapse. On the other hand, relapsing PDAP is not merely driven by persistent pathogens, but more likely stems from a pathological triad comprising uncontrolled inflammation, impaired fibrinolysis, and inadequate immune clearance. Specifically, proteomic profiling identified key biomarkers signature: in relapsing PDAP patients, CCL28, CD40, and uPA were significantly downregulated, while NRTN was markedly upregulated. This creates a microenvironment conducive to fibrosis and persistent bacterial colonization. Together, these molecular alterations paint a picture of “immune paralysis” coupled with “repair failure.” This finding highlights the need for therapeutic strategies that address local peritoneal immunity.

### 4.1 16S rDNA sequencing can serve as a supplementary method for pathogen identification

Currently, the ISPD recommends a microbiological diagnostic yield of 80%–85% (4); however, many clinical microbiology laboratories fail to achieve this benchmark, including our own department. In this study, the positivity rate using 16S rDNA sequencing was 67.8%, which was nearly identical to that of conventional culture methods (64.5%). Notably, among 11 patients with negative culture results, 6 (54.5%) were found positive by 16S rDNA sequencing. When combining both culture and 16S rDNA testing, the overall diagnostic positivity rate increased to 83.9%, approaching the ISPD-recommended standard. Therefore, we suggest that 16S rDNA sequencing may be considered a valuable complementary approach in cases where conventional cultures are negative.

### 4.2 Relapsing PDAP is associated with peritoneal immune dysregulation and reduced pathogen clearance

Peritonitis is fundamentally an inflammation, and persistent inflammation may arise from perturbations in systemic immune homeostasis driven by a confluence of intrinsic and extrinsic factors (10–13). Chuah et al. highlighted that the peritoneal cavity hosts a complex and dynamic network of immune cells essential for maintaining tissue integrity and mounting effective antimicrobial responses (14). Under pathological conditions, however, this finely tuned immune milieu can become dysregulated, resulting in unresolved inflammation and accelerated disease progression. Our results support the applicability of this theory to relapsing PDAP. Compared with patients who achieved successful recovery from peritonitis, those with relapsing peritonitis exhibited a distinct molecular signature in their peritoneal dialysate-characterized by concurrent impairment of immune function and disruption of tissue repair. Specifically, the expression levels of CCL28, CD40, and uPA were significantly reduced, whereas NRTN was markedly elevated. Therefore, we suggest that dysregulation of the immune system may be one of the contributing factors to PDAP relapse.

CCL28 is a chemokine that recruits anti-inflammatory and mucosal defense–associated immune cells, such as IgA^+^ plasmablasts and regulatory T cells (15, 16). A clinical study demonstrated that high expression of CCL28/CCR10 is crucial for recruiting antiviral memory B cells and T cells to the vaginal mucosa, thereby providing protective immunity against genital herpes (17). Meanwhile, mouse studies indicated that knockout of CCL28 reduces the frequency of herpes simplex virus-specific effector memory CD8^+^ T cells and memory B cells, consequently increasing the infection rate of herpes simplex virus (17). Another clinical study also demonstrated similar findings, showing that CCL28 mediates mucosal immune protection during HIV exposure and infection, particularly by promoting HIV-specific IgA responses (18). In this study, lower CCL28 levels in relapsing PDAP patients are linked to impaired immune surveillance and pathogen clearance. This aligns with our observed situation of immune microenvironment alterations, pathogen persistence, and disease relapse.Therefore, the downregulation of CCL28 may compromise local immune surveillance and pathogen clearance within the peritoneum.

CD40, a critical co-stimulatory molecule, plays an essential role in facilitating interactions between antigen-presenting cells and T cells (19, 20). Findings in both human and rat models demonstrate that peritoneal mesothelial cells functionally express CD40, suggesting that the CD40/CD40L axis contributes to localized peritoneal inflammation and T-cell-mediated immune responses (21, 22). In mice, B cells engage in CD40-CD40L interactions via CD40 expression, which is essential for establishing sustained immunity against Pneumocystis, indicating that B cells function not only as antibody producers but also as critical antigen-presenting cells (23). Clinically, genetic polymorphisms in CD40 correlate with HBV infection outcomes. Specifically, the C allele of rs1883832 upregulates CD40 expression, enhances CD40/CD40L-mediated cytotoxic T lymphocyte responses, and promotes HBV clearance and immune recovery (24). In contrast, CD40-deficient patients display increased susceptibility to recurrent infections, pathogen burden, and associated complications (25). At present, hematopoietic stem cell transplantation represents the most promising curative strategy for such patients (25). Therefore, the reduced expression of CD40 likely impairs adaptive immune responses.

uPA is involved in macrophage migration, fibrinolysis, and tissue remodeling (26–28). Its deficiency may hinder inflammatory cell infiltration while promoting fibrin deposition, thereby increasing the risk of peritoneal adhesions or encapsulating peritonitis. uPA promotes monocyte-to-macrophage differentiation and inhibits macrophage apoptosis through the uPAR-dependent ERK1/2-Bim pathway (29). Correspondingly, uPAR-/- mice exhibit a 30% reduction in peritoneal macrophage numbers. In mice expressing uPA specifically in macrophages, macrophage-derived uPA acts as a key driver of muscle regeneration, coordinating tissue repair by enhancing macrophage recruitment, angiogenesis, and myogenesis (30). Separately, uPA also functions as an endogenous antimicrobial molecule that contributes to host defense against *Staphylococcus aureus* infection (31). Its activity at infection sites is regulated by plasminogen activator inhibitor type 1 (PAI-1), implying that PAI-1 overexpression may compromise innate immunity. Collectively, these findings suggest that diminished uPA levels likely impair local immune clearance and tissue repair capacity.

NRTN is a neurotrophic factor primarily involved in neuronal survival, but emerging evidence suggests it also modulates immunity (32, 33). The aberrant elevation of NRTN may reflect a compensatory attempt by the body to activate neural-immune regulatory pathways for tissue repair under persistent inflammatory stress (34–36). For example, NRTN is essential for ocular surface homeostasis in mice; its deficiency leads to neurogenic dry eye with chronic inflammation (37). In models of allergic airway inflammation, NRTN deficiency exacerbates disease progression by amplifying Th2-type immune responses and inflammatory pathology (38). Furthermore, in a complete Freund’s adjuvant-induced skin inflammation model, keratinocyte-derived NRTN contributes to early immune regulation by upregulating TNF-α and IL-1β, initiating protective responses and supporting injury repair (35). However, without adequate immune activation and extracellular matrix remodeling, this compensatory response may appear insufficient to reverse disease progression. Therefore, in our study, upregulated NRTN in relapsing peritonitis may reflect a reparative or immunoregulatory response to recurrent inflammation, possibly via interactions with the gut-peritoneal axis.

### 4.3 Antibiotic-mediated dysbiosis and immune consequences

Indeed, broad-spectrum antibiotics, while necessary for eradicating the primary infection, act as a double-edged sword by inducing significant microbial dysbiosis (39, 40). Although our study focused on baseline (pre-treatment) signatures, we recognize that antibiotic-mediated ecological disruption may be a factor in the relapse mechanism. First, antibiotics exert selective pressure that facilitates the “rebound” of resistant pathogens. As shown in Section 3.3, relapsing patients harbored aggressive, antibiotic-resistant species (e.g., *Pseudomonas aeruginosa*) prior to treatment. Standard regimens may fail to eliminate these strains, leading to their post-antibiotic proliferation. Furthermore, antibiotics disrupt competitive exclusion within the microbial community, allowing typically suppressed gut-derived anaerobes to colonize the peritoneal cavity once susceptible species are cleared.

Second, this dysbiosis directly impairs local immune reconstitution, creating a state of “immune paralysis” (41). A healthy microbiome provides tonic stimulation essential for mucosal immunity. Its disruption by antibiotics likely contributes to the downregulation of key immune mediators we observed in the Relapse group. CCL28 recruits IgA+ plasma cells and regulatory T cells (15, 16). Reduced antigenic stimulation due to dysbiosis may suppress CCL28 expression, compromising the recruitment of effector cells needed for final pathogen clearance. The downregulation of CD40, a critical co-stimulatory molecule, may reflect a state of “endotoxin tolerance” (42). Chronic exposure to translocating microbial products (e.g., LPS) due to gut barrier dysfunction can desensitize peritoneal macrophages, impairing their bactericidal capacity despite the presence of infection. The uPA system is vital for macrophage migration and fibrinolysis (26–28). Dysbiosis alters microbial metabolites (e.g., short-chain fatty acids), which modulate macrophage function. Reduced uPA levels suggest a failure in tissue repair, potentially exacerbated by an antibiotic-altered metabolic environment.

In summary, we hypothesize that antibiotics may inadvertently perpetuate the relapse cycle by selecting for resistant strains and disrupting the host-microbiome symbiosis. This leads to a failure in the “inflammation-fibrinolysis-repair” axis, manifesting as the immune dysregulation signature (CCL2, CD40, uPA) identified in our study. Conversely, the upregulation of NRTN may represent a compensatory, albeit insufficient, attempt to restore homeostasis under this dysbiotic stress. Future longitudinal studies are needed to validate this causal link.

### 4.4 Limitations

This study has several limitations. First, the relatively small sample size may introduce selection bias, potentially affecting the generalizability and robustness of the findings. Second, although we identified several potential biomarkers associated with peritonitis recurrence, their clinical relevance and predictive value require further validation in larger, multicenter cohorts. Additionally, while our clinical cohort analysis highlighted potential therapeutic targets, the underlying molecular mechanisms remain unclear. Future studies should employ experimental approaches to thoroughly investigate the functional roles and signaling pathways of these targets in the pathogenesis and progression of peritonitis, thereby strengthening the theoretical foundation for their use as intervention strategies. Third, long-term antibiotic treatment may alter the inflammatory microenvironment; therefore, its potential impact on inflammatory biomarkers cannot be excluded. Fourth, this study reported proteomic data from the dialysate exclusively, without measuring corresponding serum levels. Future studies should include paired serum and dialysate samples to distinguish between local and systemic contributions. Fifth, we acknowledge that sampling from PD catheters carries a risk of contamination. In this study, dialysate samples were collected under strict aseptic techniques, and all proteomic and 16S rRNA analyses included negative controls.

## 5 Conclusions

In conclusion, PDAP relapse is a multifactorial process underpinned by host immune dysregulation and disrupted gut microbial homeostasis. Our work identifies the CCL28/CD40/uPA/NRTN panel as a potential predictive biomarker signature for relapse and suggests novel therapeutic avenues. These molecular changes reflect a pathological state of relapsing peritonitis marked by compromised immune defense, unresolved inflammation, and dysregulated tissue repair. Furthermore, 16S rDNA sequencing provides a supplementary approach for pathogen detection. Collectively, these findings may advance the prospect of precision management in PDAP and lead to improved clinical outcomes.

## Data Availability

The 16S data presented in the study are deposited in the NCBI GEO repository, accession number PRJNA1482158. Protein profiling was performed using Olink sequencing. The raw data can be found in the **Supplementary Material**.
