# Supplementary figures and images for: Immune dysregulation drives the relapse of peritoneal dialysis-associated peritonitis: a single-center prospective study
Source: Front Immunol. 2026 Jul 3;17:1810227. doi: 10.3389/fimmu.2026.1810227 (PMC13375459; doi:10.3389/fimmu.2026.1810227)

# Inflammation

IQR

QC\_Warning

Pass

Sample Median

8  
6  
4  
2

0

2

4

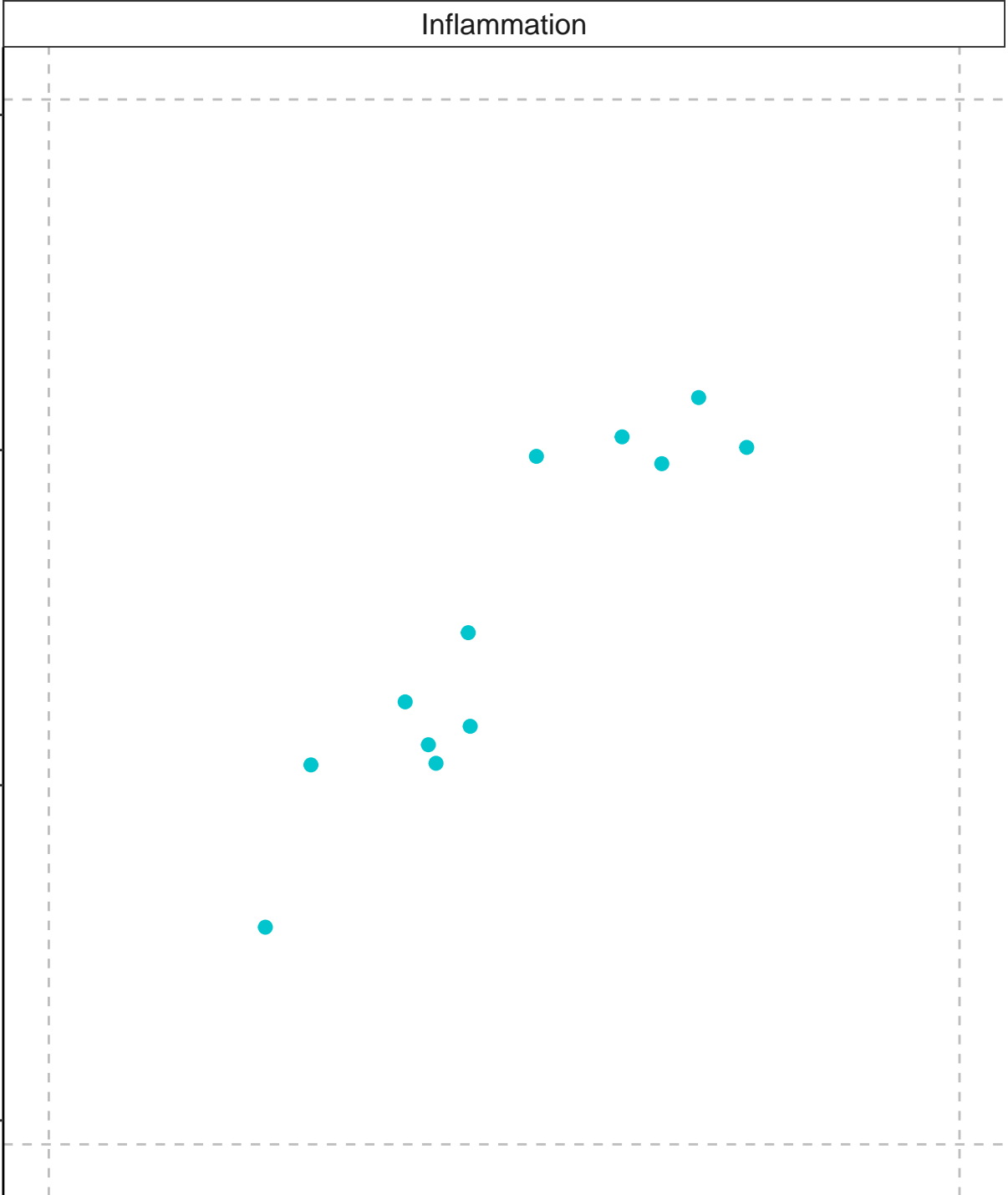

Supplement: Supplementary file 1 [file DataSheet1.zip › summary of proteomics/summary/02.QC/QC_IQR_1.pdf]

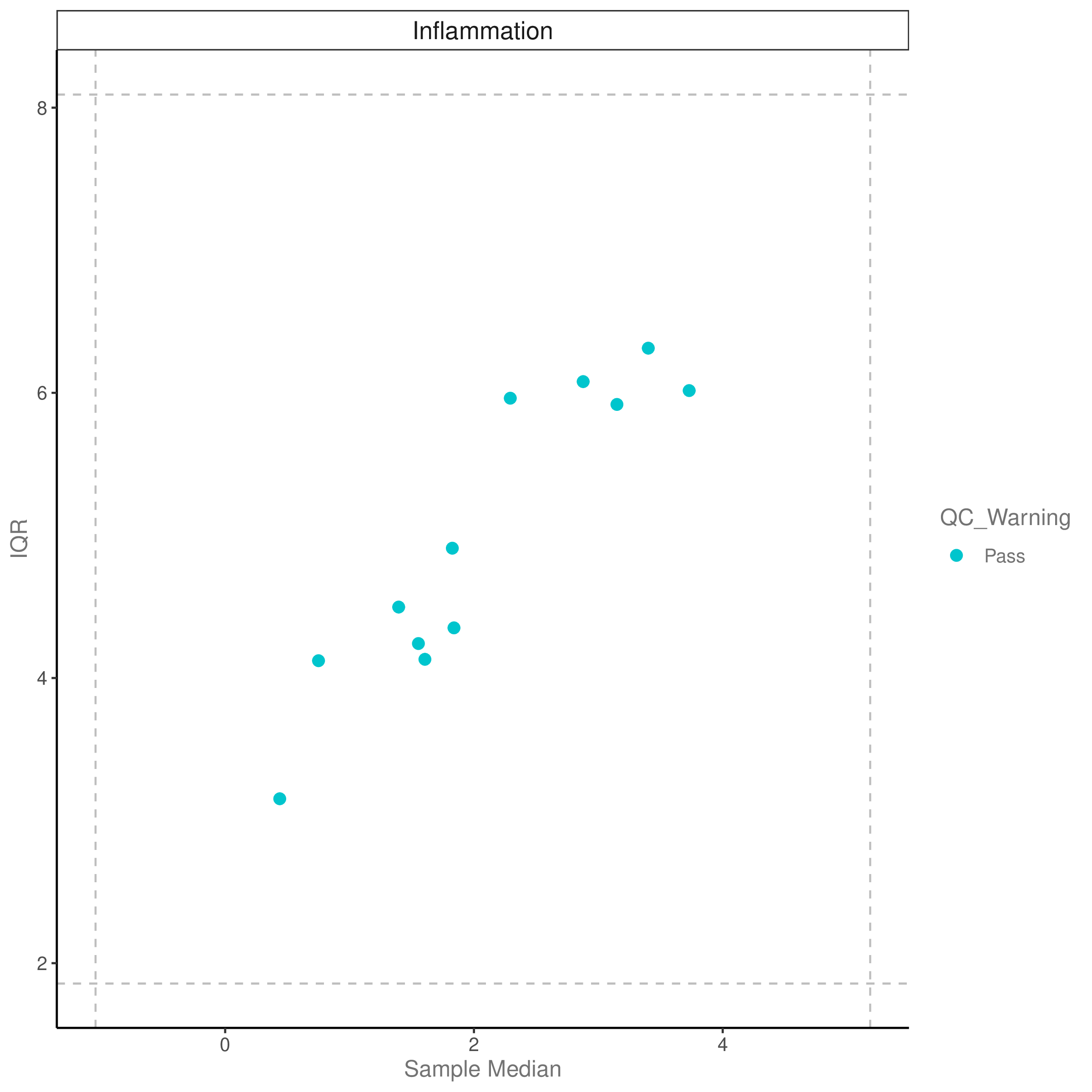

Supplement: Supplementary file 1 [file DataSheet1.zip › summary of proteomics/summary/02.QC/QC_IQR_1.png]

# Inflammation

NPX

10

5

0

QC\_Warning

Pass

FH\_1

FH\_2

FH\_3

FH\_4

FH\_5

FH\_6

ZH\_1

ZH\_2

ZH\_3

ZH\_4

ZH\_5

ZH\_6

Samples

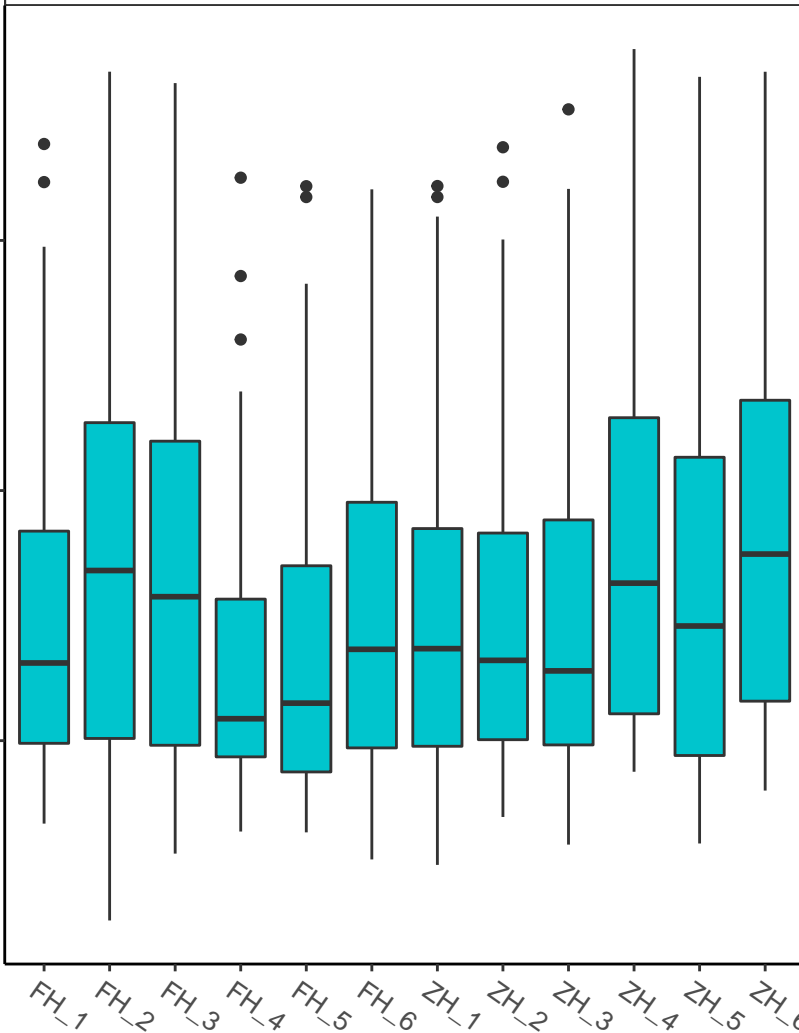

Supplement: Supplementary file 1 [file DataSheet1.zip › summary of proteomics/summary/02.QC/QC_NPX_distribution_1.pdf]

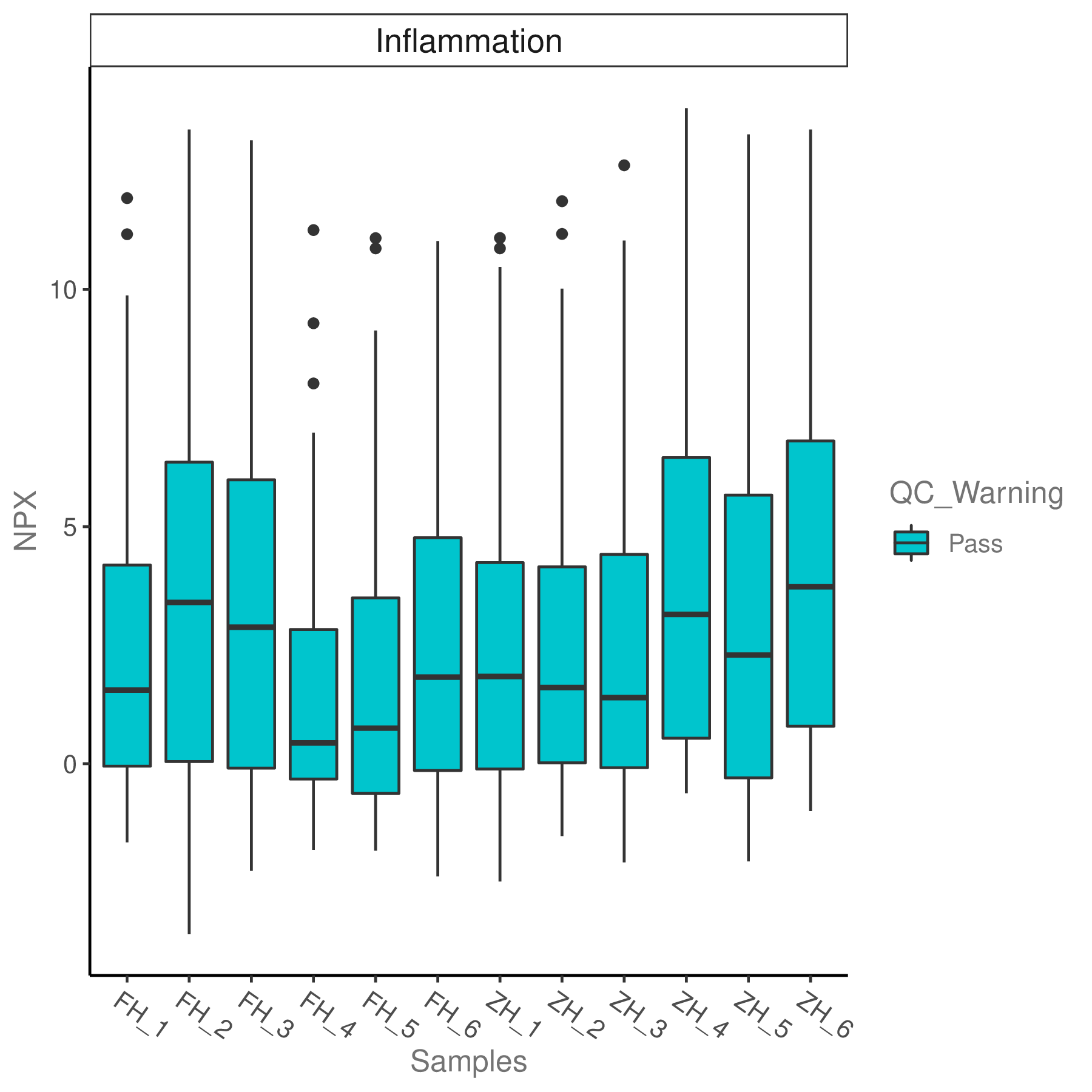

Supplement: Supplementary file 1 [file DataSheet1.zip › summary of proteomics/summary/02.QC/QC_NPX_distribution_1.png]

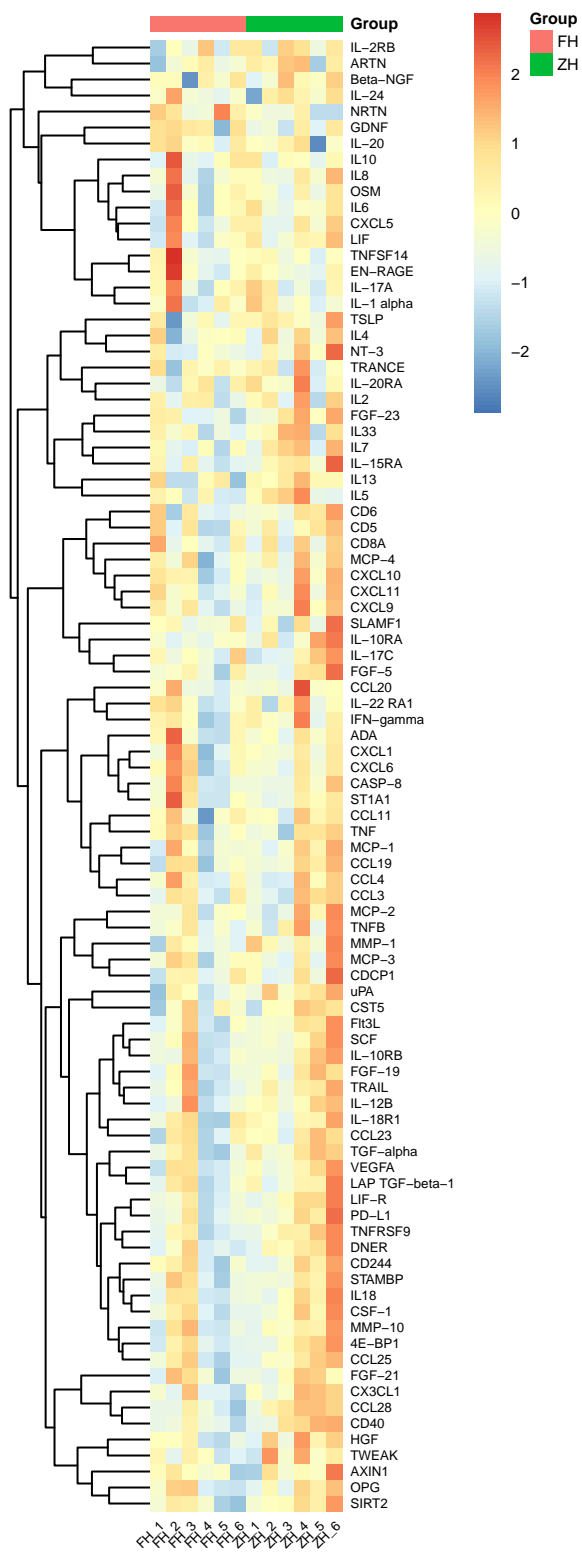

Supplement: Supplementary file 1 [file DataSheet1.zip › summary of proteomics/summary/03.Profile/all_heatmap.pdf]

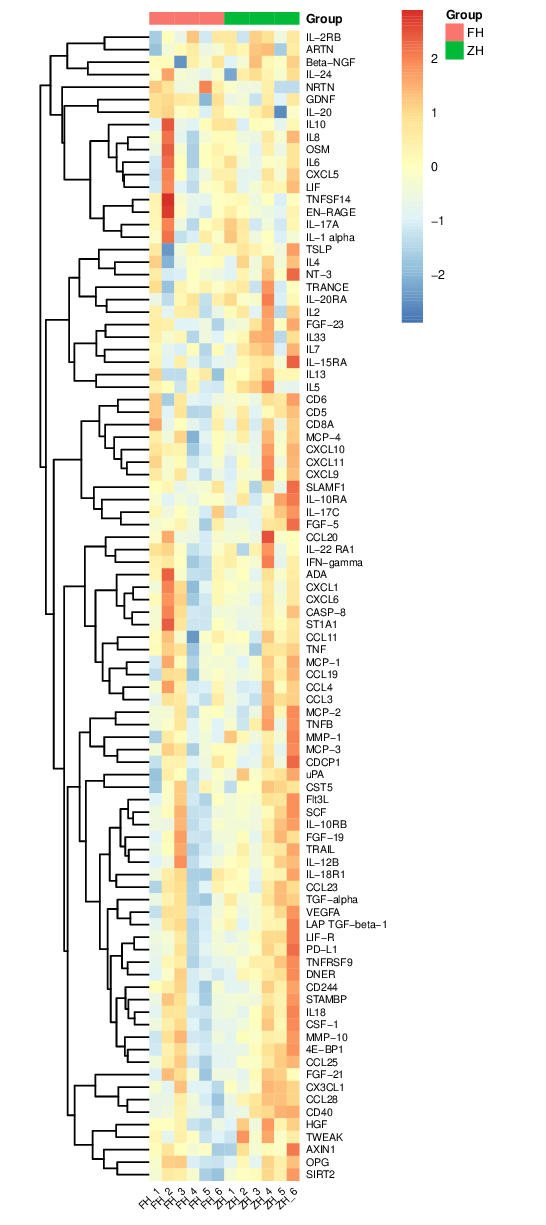

Supplement: Supplementary file 1 [file DataSheet1.zip › summary of proteomics/summary/03.Profile/all_heatmap.png]

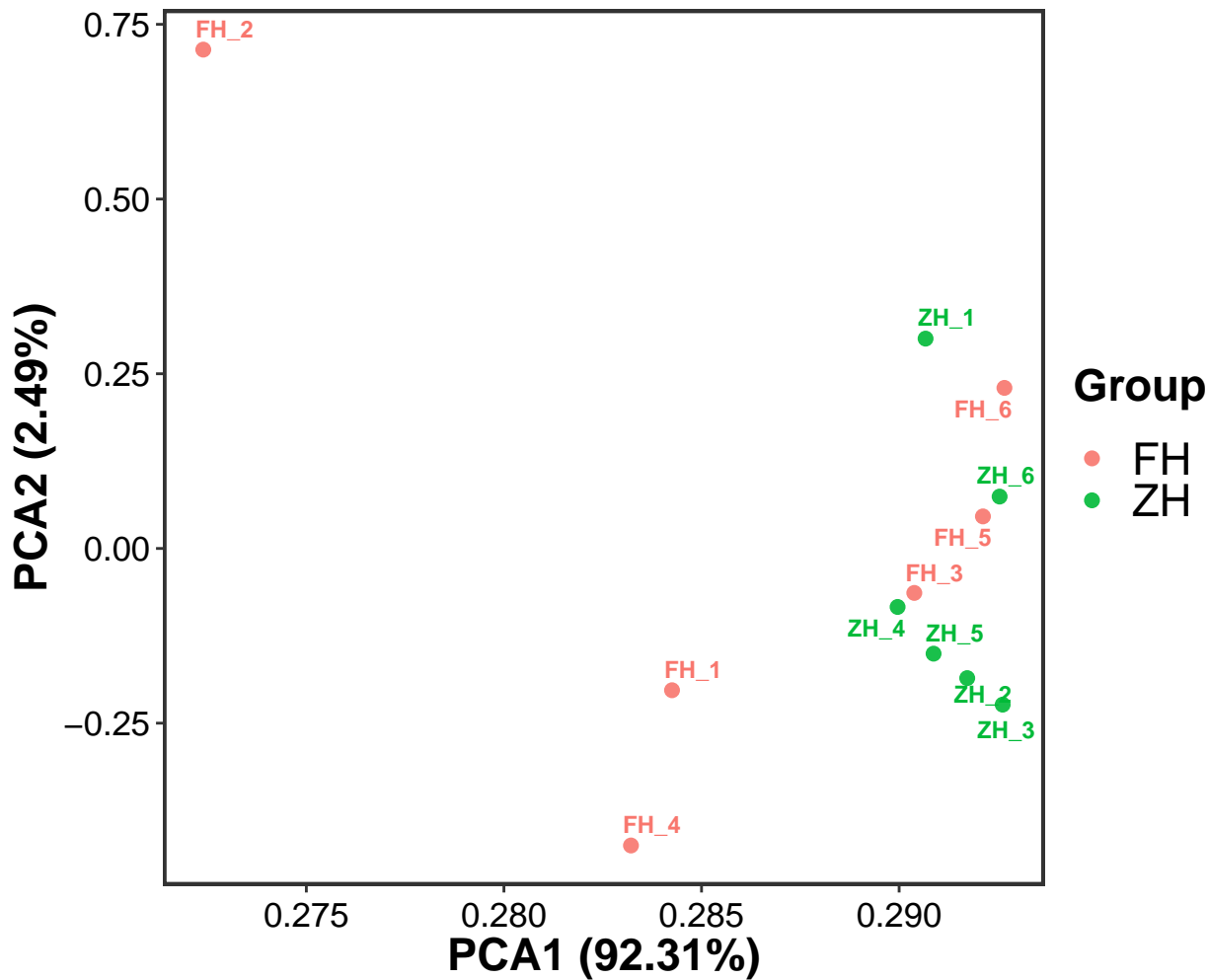

Supplement: Supplementary file 1 [file DataSheet1.zip › summary of proteomics/summary/03.Profile/all_pca.pdf]

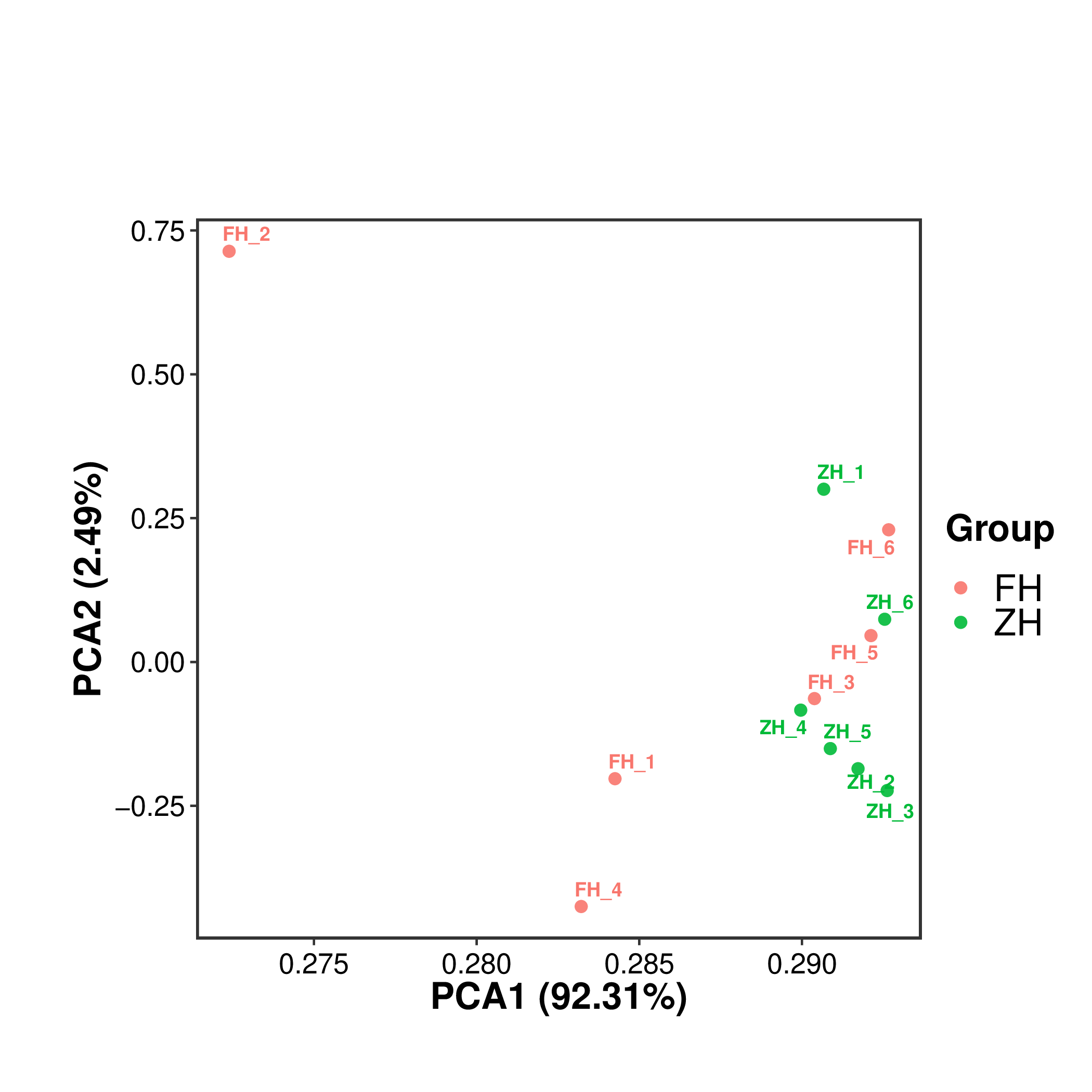

Supplement: Supplementary file 1 [file DataSheet1.zip › summary of proteomics/summary/03.Profile/all_pca.png]

tSNE Plot

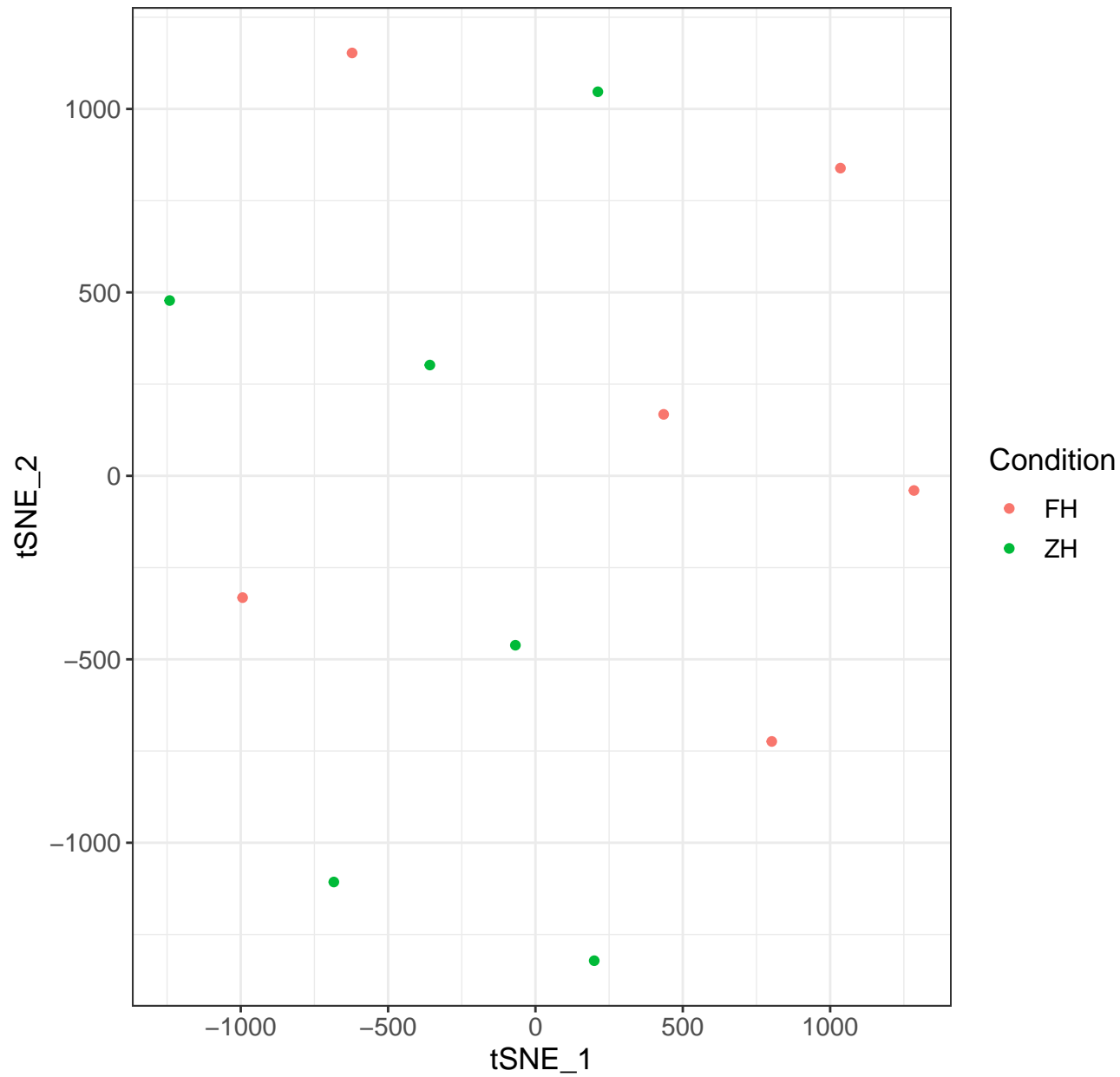

Supplement: Supplementary file 1 [file DataSheet1.zip › summary of proteomics/summary/03.Profile/all_tsne.pdf]

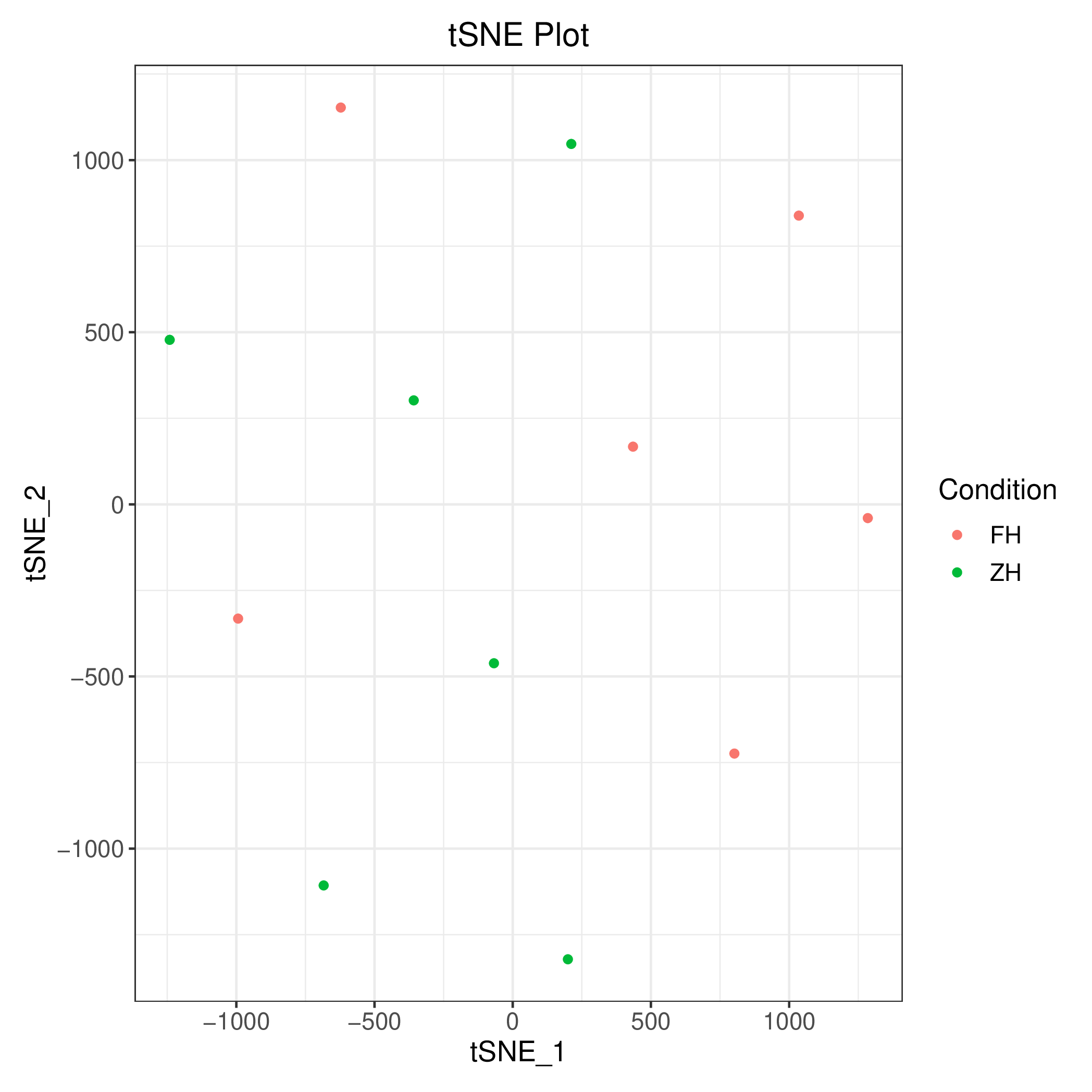

Supplement: Supplementary file 1 [file DataSheet1.zip › summary of proteomics/summary/03.Profile/all_tsne.png]

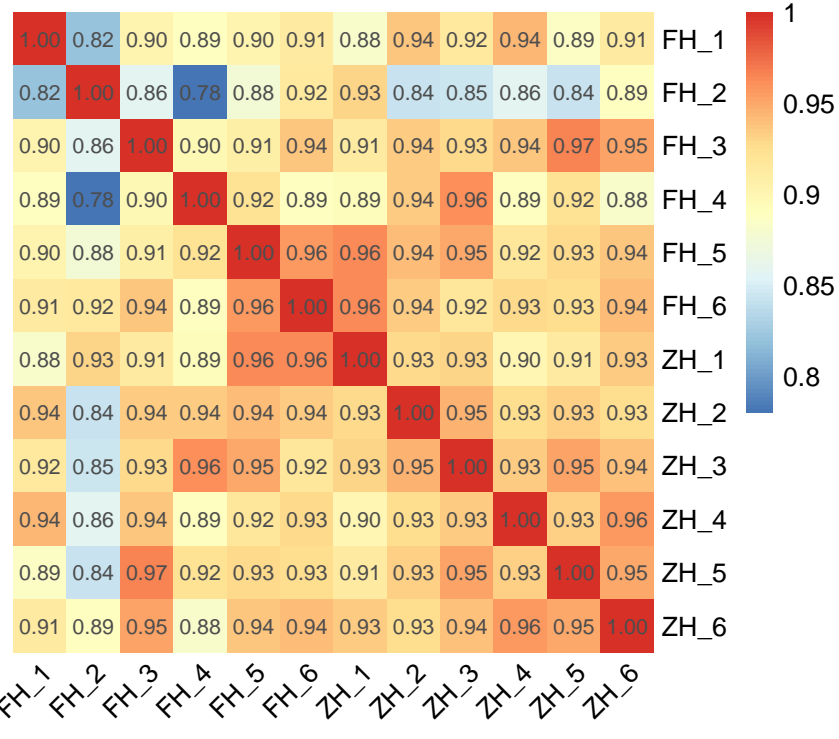

Supplement: Supplementary file 1 [file DataSheet1.zip › summary of proteomics/summary/03.Profile/cor_heatmap.pdf]

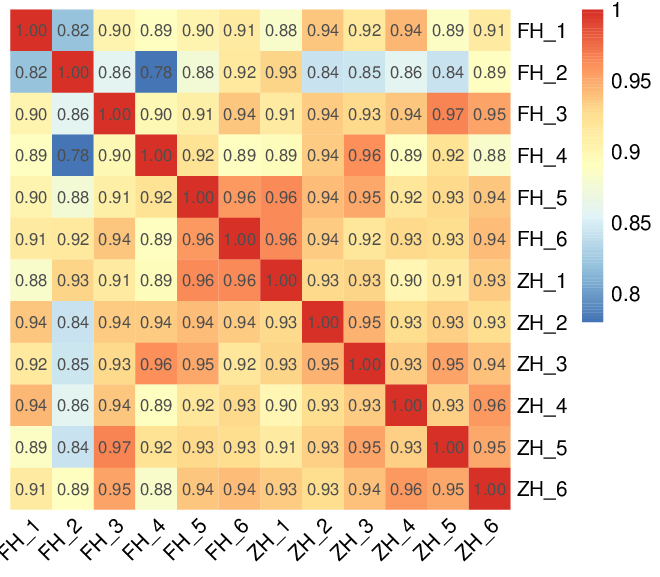

Supplement: Supplementary file 1 [file DataSheet1.zip › summary of proteomics/summary/03.Profile/cor_heatmap.png]

# Differentially expressed proteins in different groups

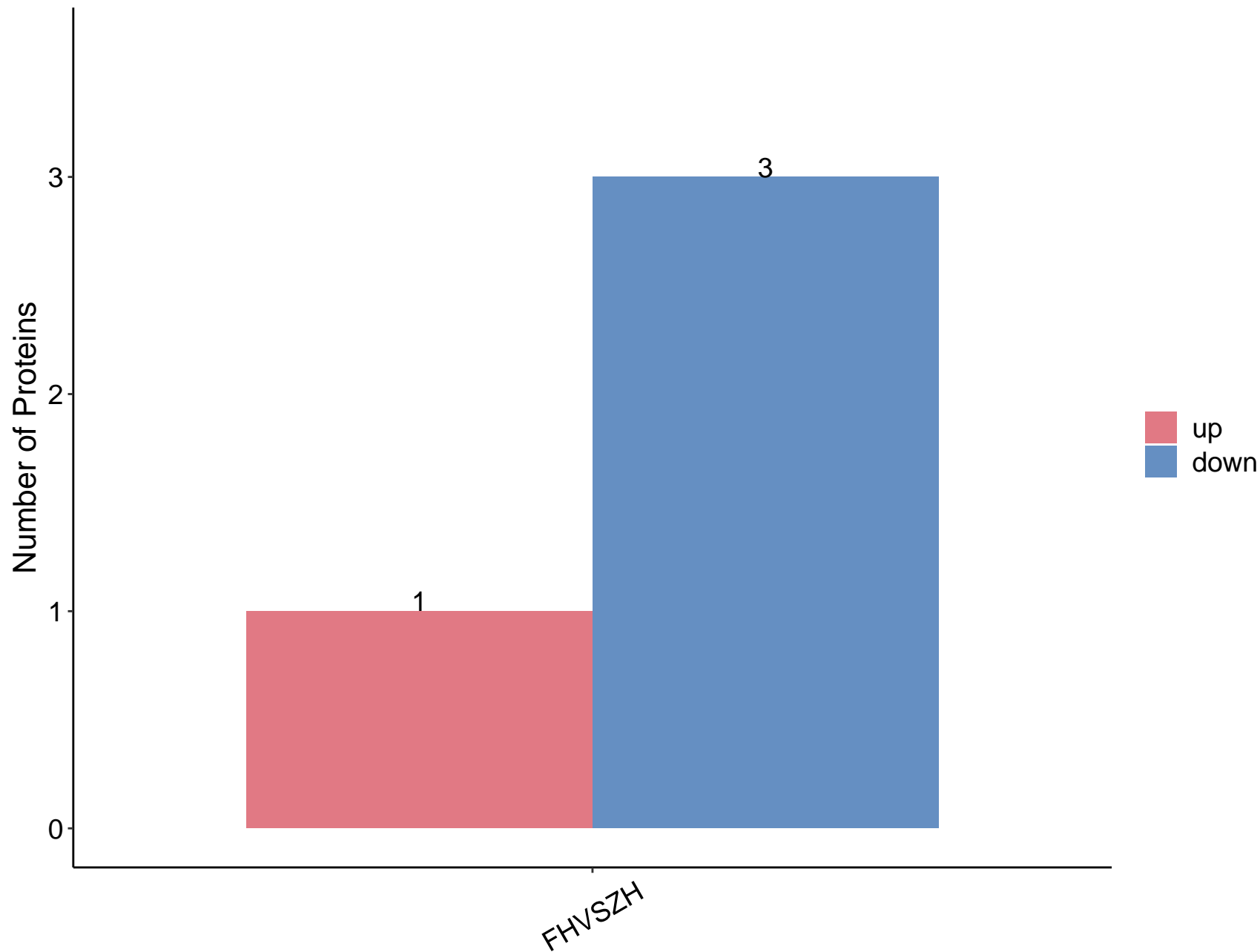

Supplement: Supplementary file 1 [file DataSheet1.zip › summary of proteomics/summary/04.Diff_analysis/COND1/COND1_diff_regulation.pdf]

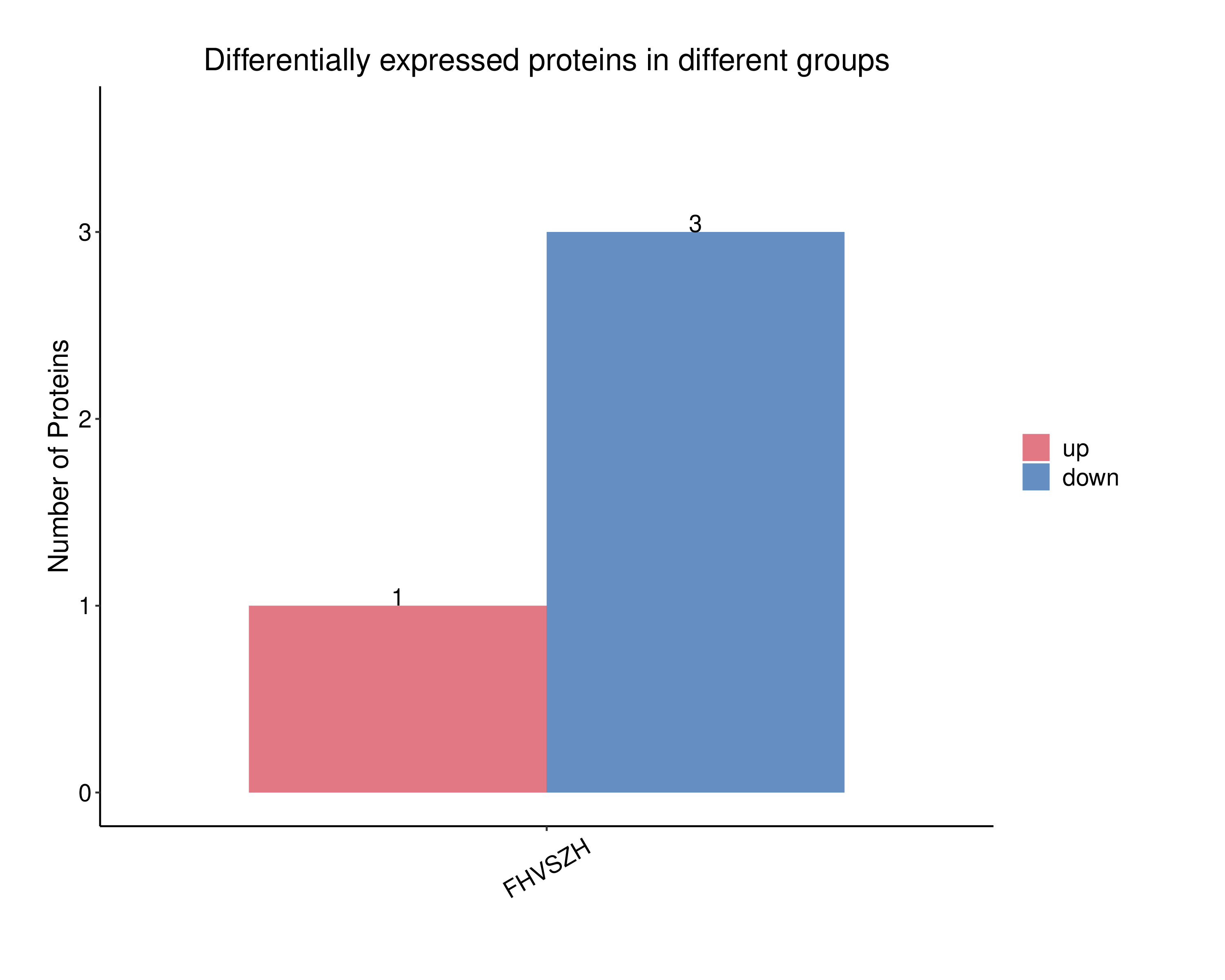

Supplement: Supplementary file 1 [file DataSheet1.zip › summary of proteomics/summary/04.Diff_analysis/COND1/COND1_diff_regulation.png]

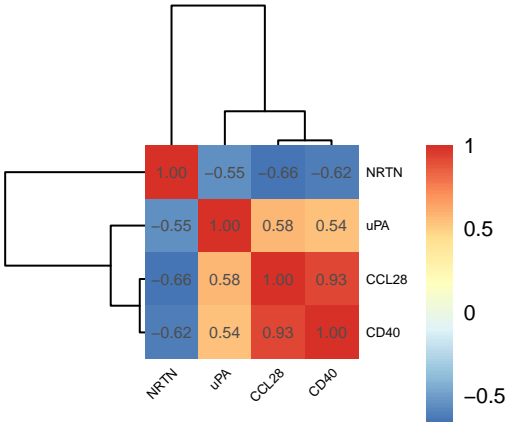

Supplement: Supplementary file 1 [file DataSheet1.zip › summary of proteomics/summary/04.Diff_analysis/COND1/FHVSZH/Diff_protein_cor/FHVSZH_cor_heatmap.pdf]

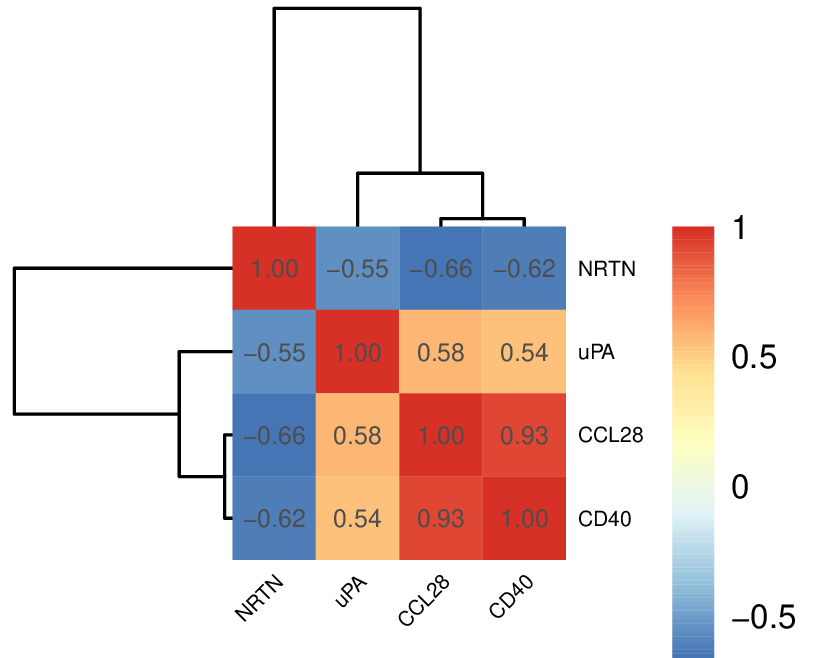

Supplement: Supplementary file 1 [file DataSheet1.zip › summary of proteomics/summary/04.Diff_analysis/COND1/FHVSZH/Diff_protein_cor/FHVSZH_cor_heatmap.png]

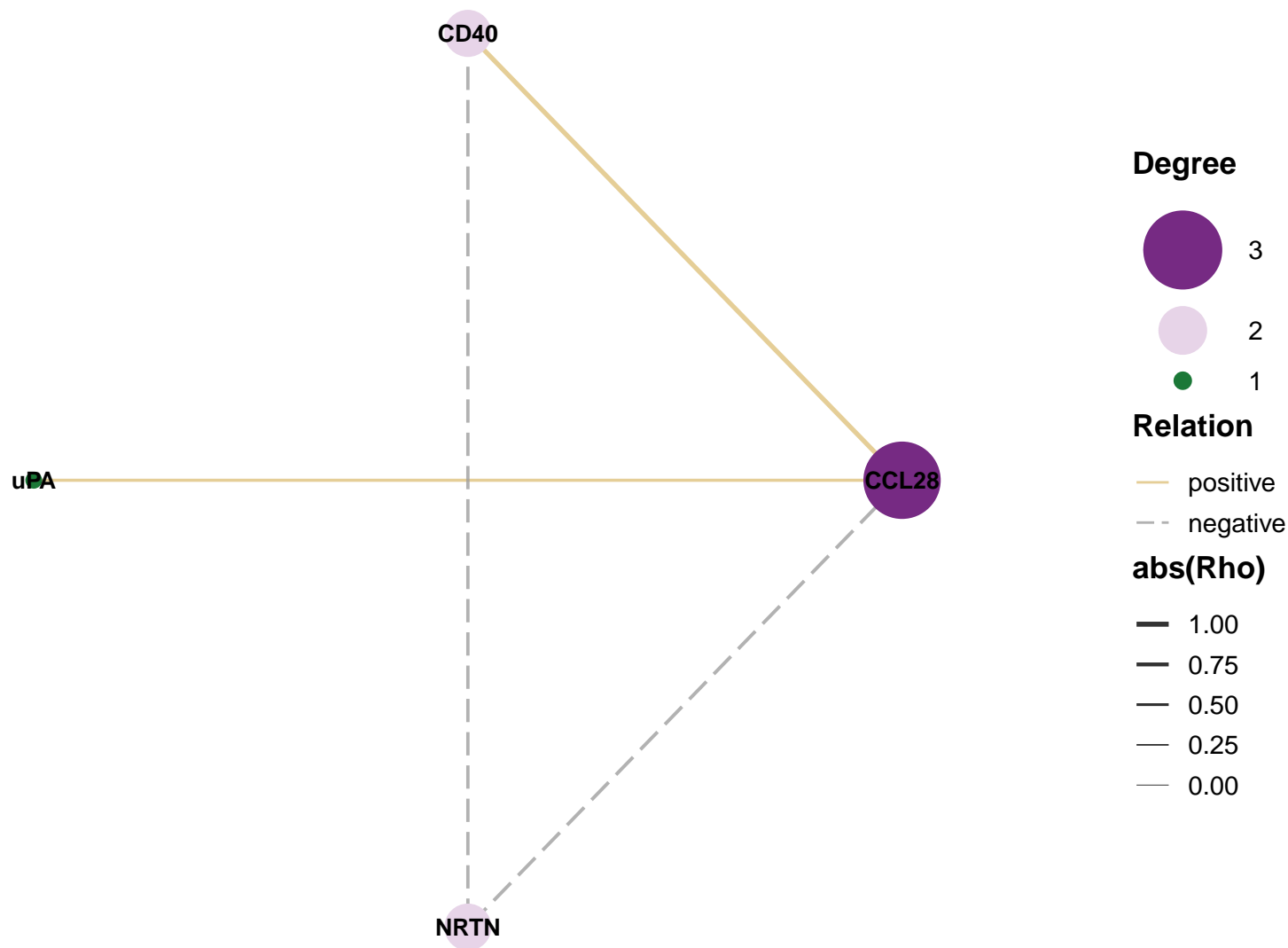

Supplement: Supplementary file 1 [file DataSheet1.zip › summary of proteomics/summary/04.Diff_analysis/COND1/FHVSZH/Diff_protein_cor/FHVSZH_cor_protein_heatmap.pdf]

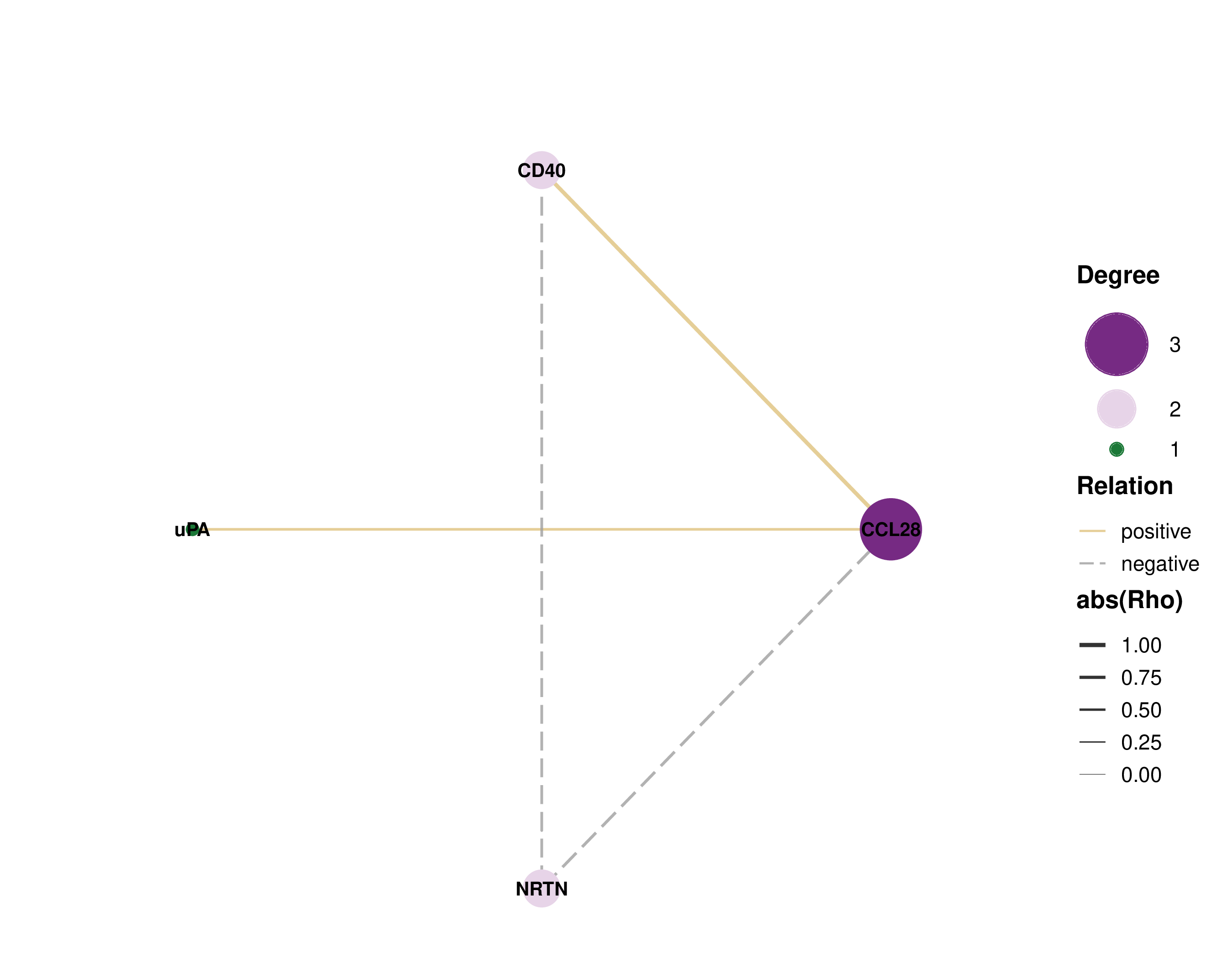

Supplement: Supplementary file 1 [file DataSheet1.zip › summary of proteomics/summary/04.Diff_analysis/COND1/FHVSZH/Diff_protein_cor/FHVSZH_cor_protein_heatmap.png]

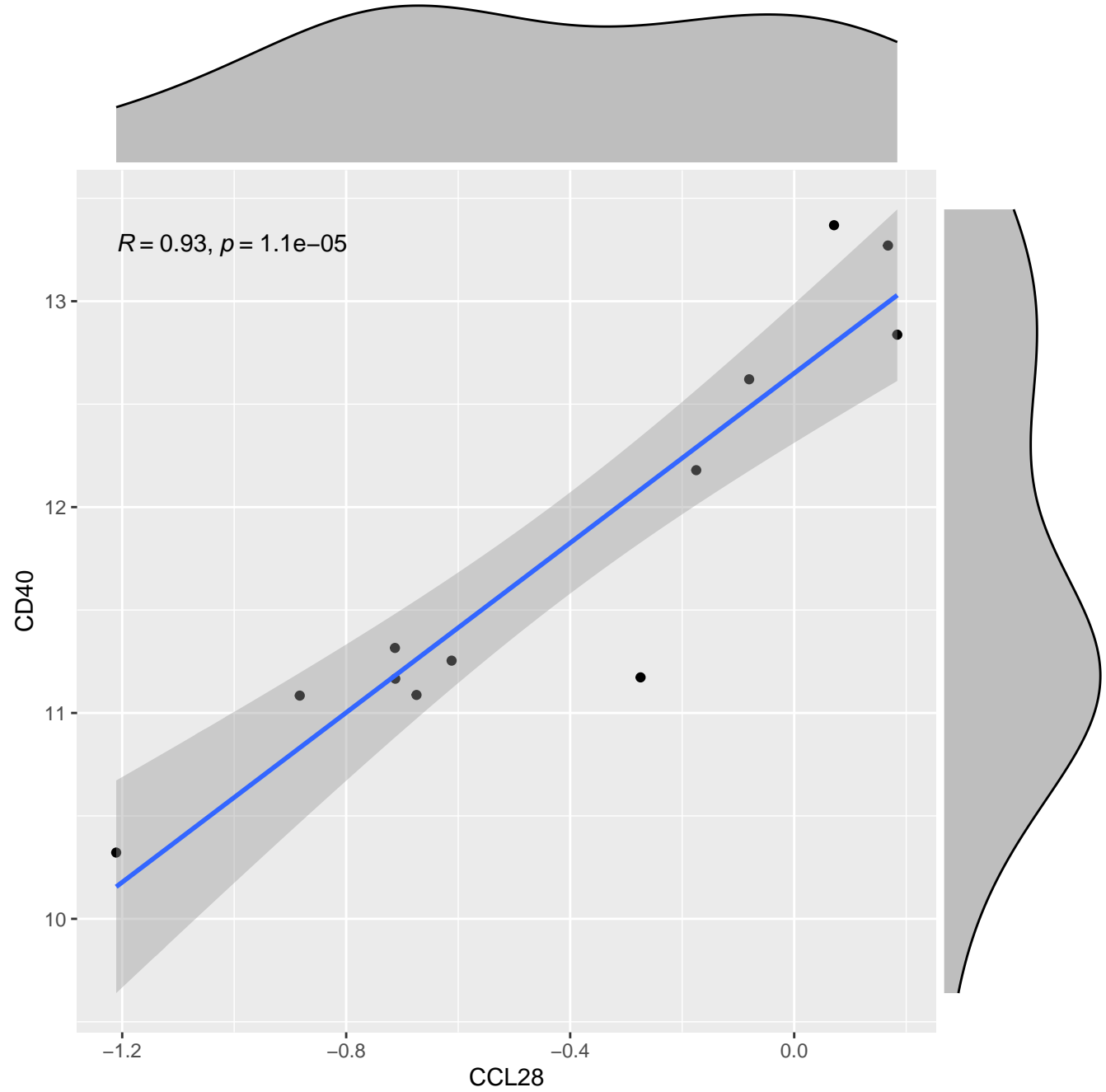

Supplement: Supplementary file 1 [file DataSheet1.zip › summary of proteomics/summary/04.Diff_analysis/COND1/FHVSZH/Diff_protein_cor/FHVSZH_max_pro_point.pdf]

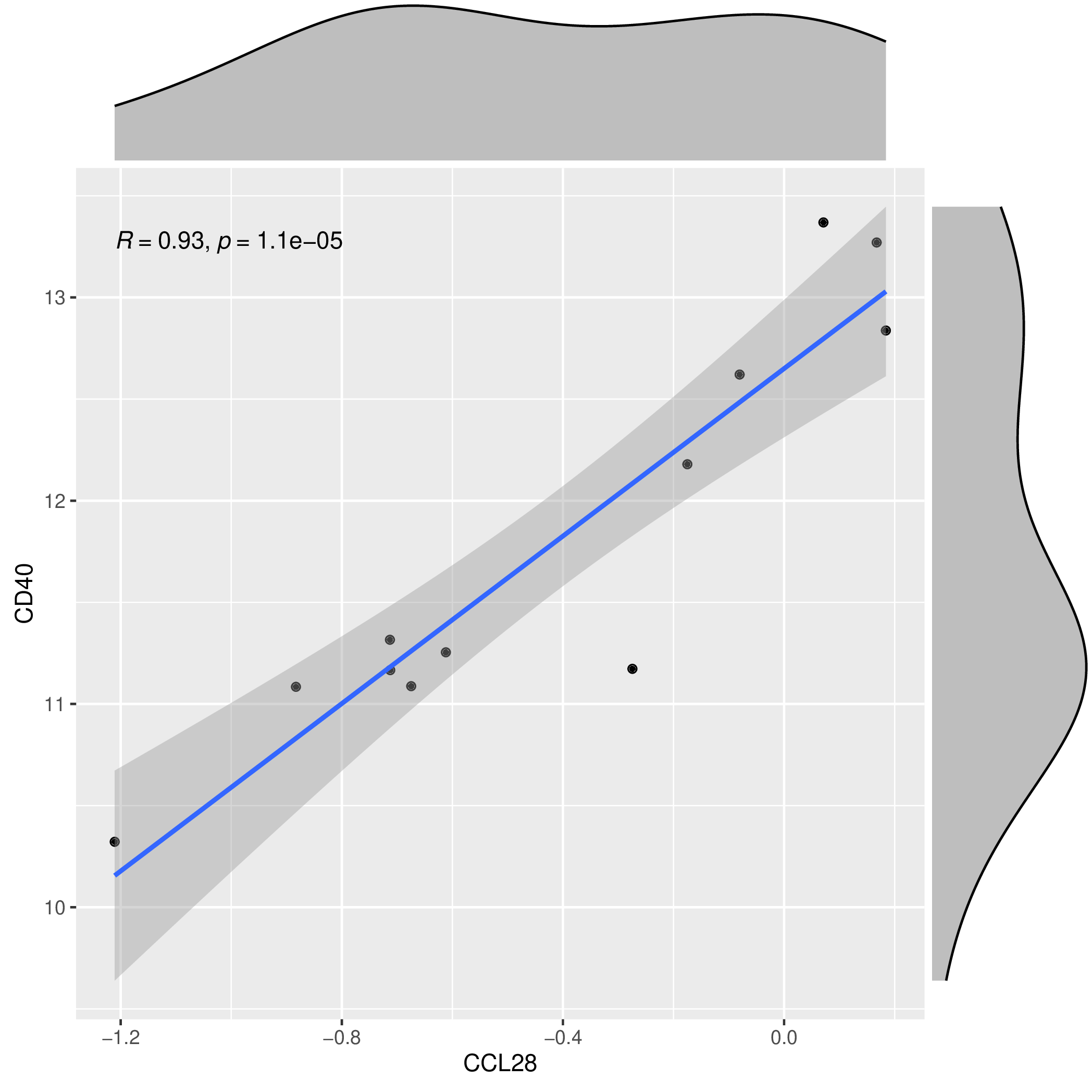

Supplement: Supplementary file 1 [file DataSheet1.zip › summary of proteomics/summary/04.Diff_analysis/COND1/FHVSZH/Diff_protein_cor/FHVSZH_max_pro_point.png]

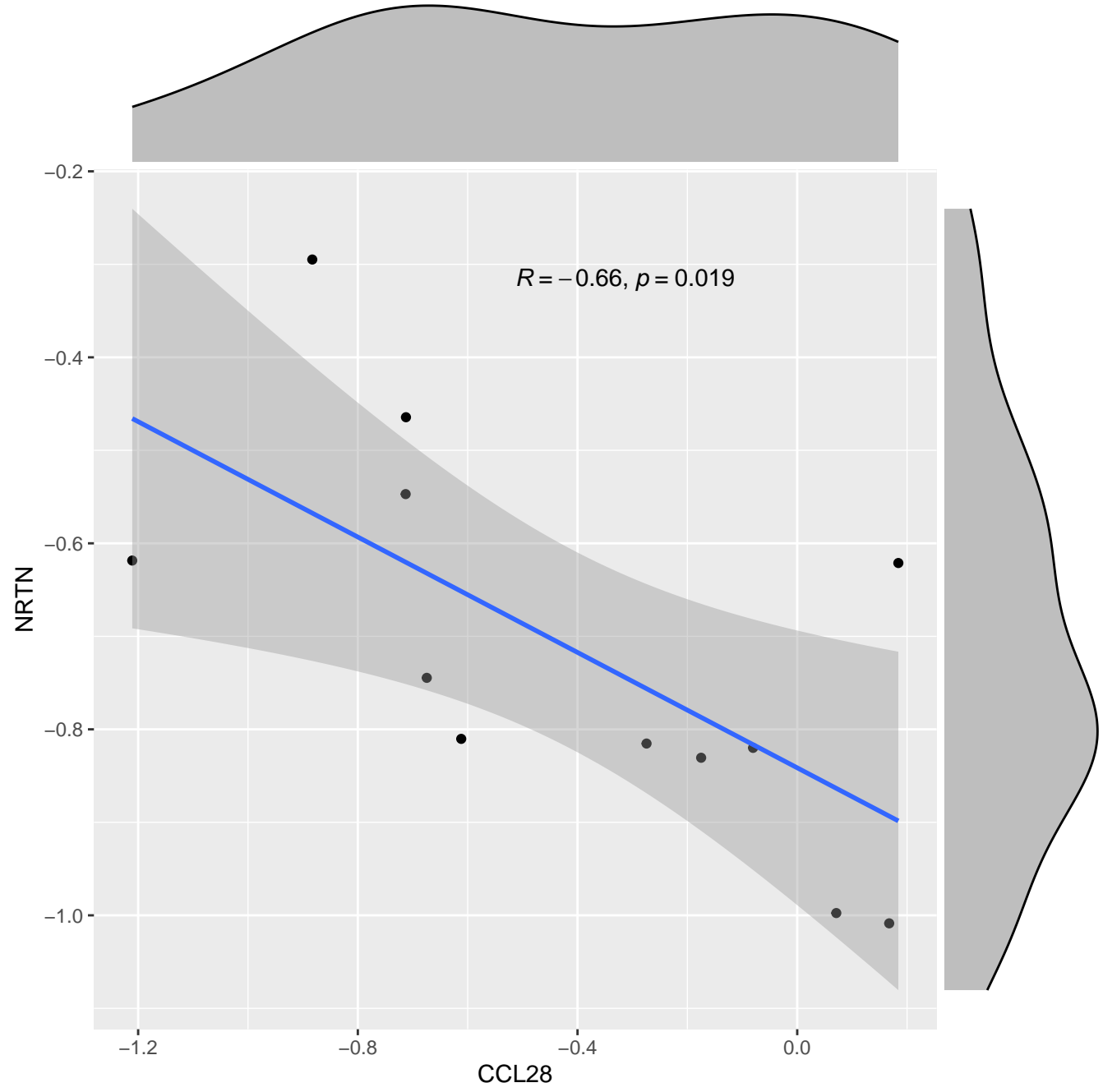

Supplement: Supplementary file 1 [file DataSheet1.zip › summary of proteomics/summary/04.Diff_analysis/COND1/FHVSZH/Diff_protein_cor/FHVSZH_min_pro_point.pdf]

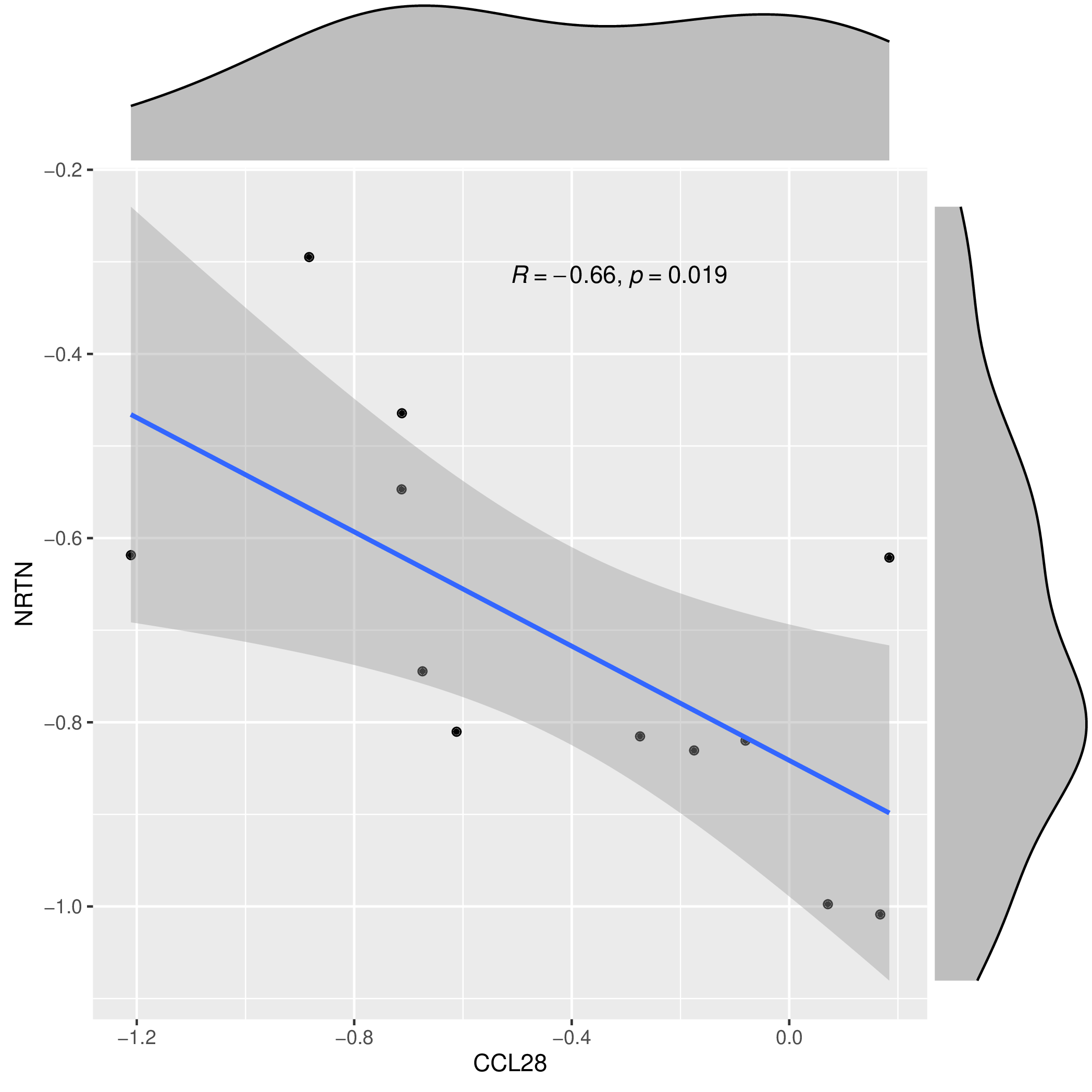

Supplement: Supplementary file 1 [file DataSheet1.zip › summary of proteomics/summary/04.Diff_analysis/COND1/FHVSZH/Diff_protein_cor/FHVSZH_min_pro_point.png]

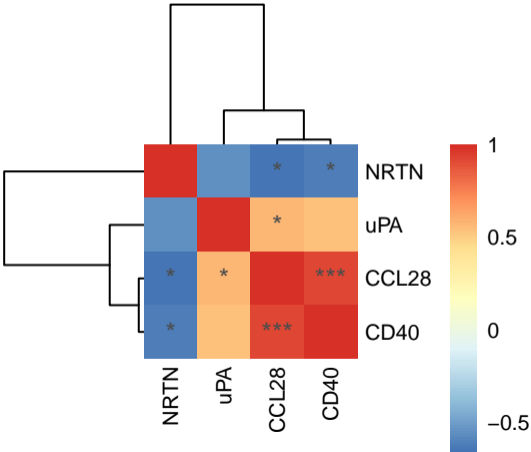

Supplement: Supplementary file 1 [file DataSheet1.zip › summary of proteomics/summary/04.Diff_analysis/COND1/FHVSZH/Diff_protein_cor/FHVSZH_p_heatmap.pdf]

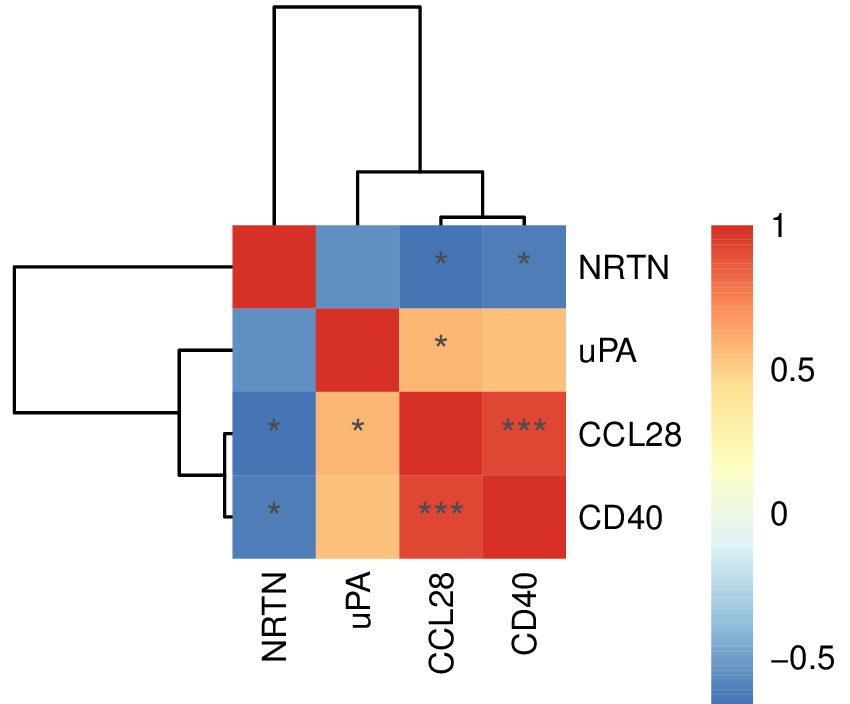

Supplement: Supplementary file 1 [file DataSheet1.zip › summary of proteomics/summary/04.Diff_analysis/COND1/FHVSZH/Diff_protein_cor/FHVSZH_p_heatmap.png]

Top 20 of DO Enrichment

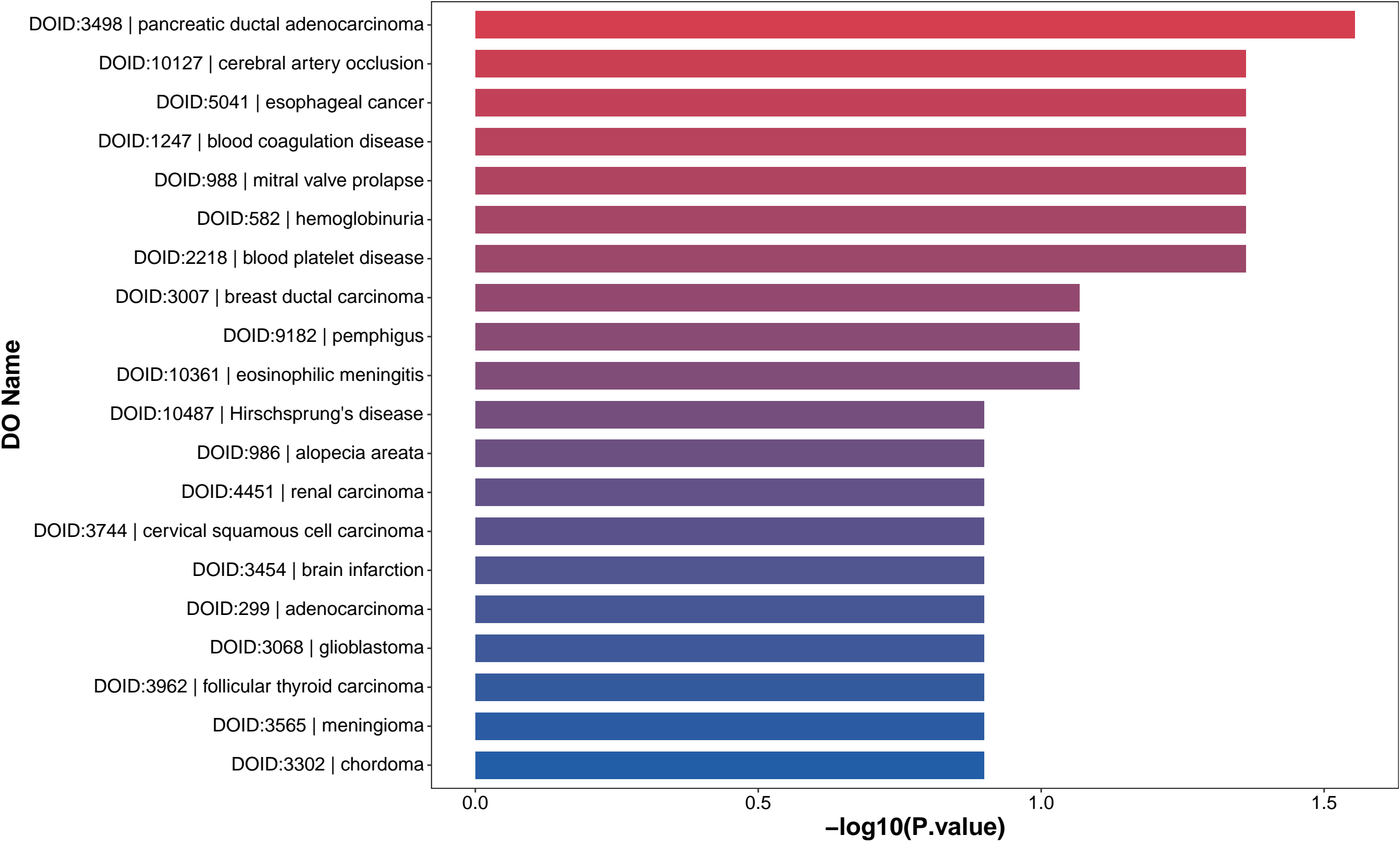

Supplement: Supplementary file 1 [file DataSheet1.zip › summary of proteomics/summary/04.Diff_analysis/COND1/FHVSZH/Enrichment/DO/COND1.FHVSZH.DO_Enrichment.P.pdf]

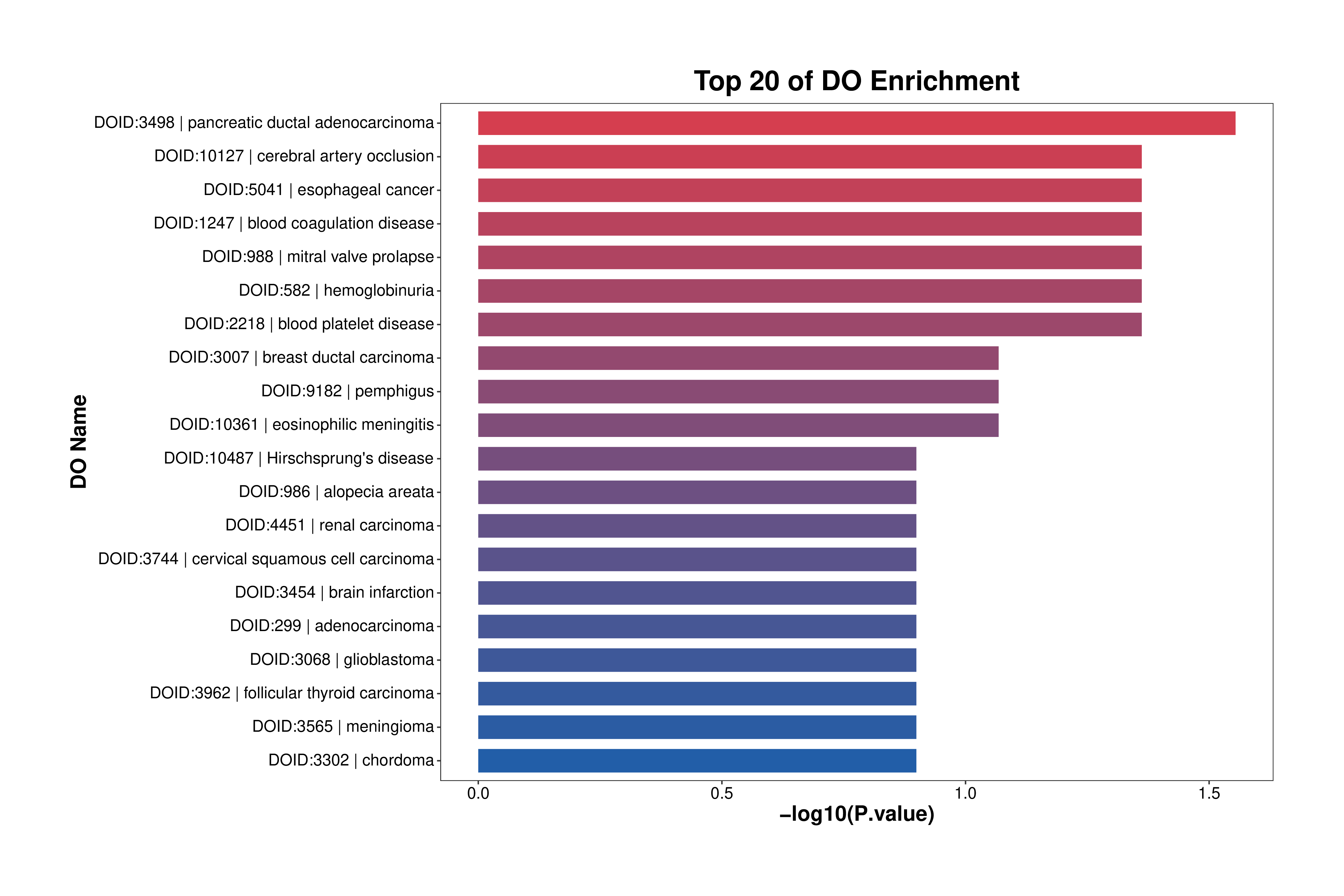

Supplement: Supplementary file 1 [file DataSheet1.zip › summary of proteomics/summary/04.Diff_analysis/COND1/FHVSZH/Enrichment/DO/COND1.FHVSZH.DO_Enrichment.P.png]

Top 20 of DO Enrichment

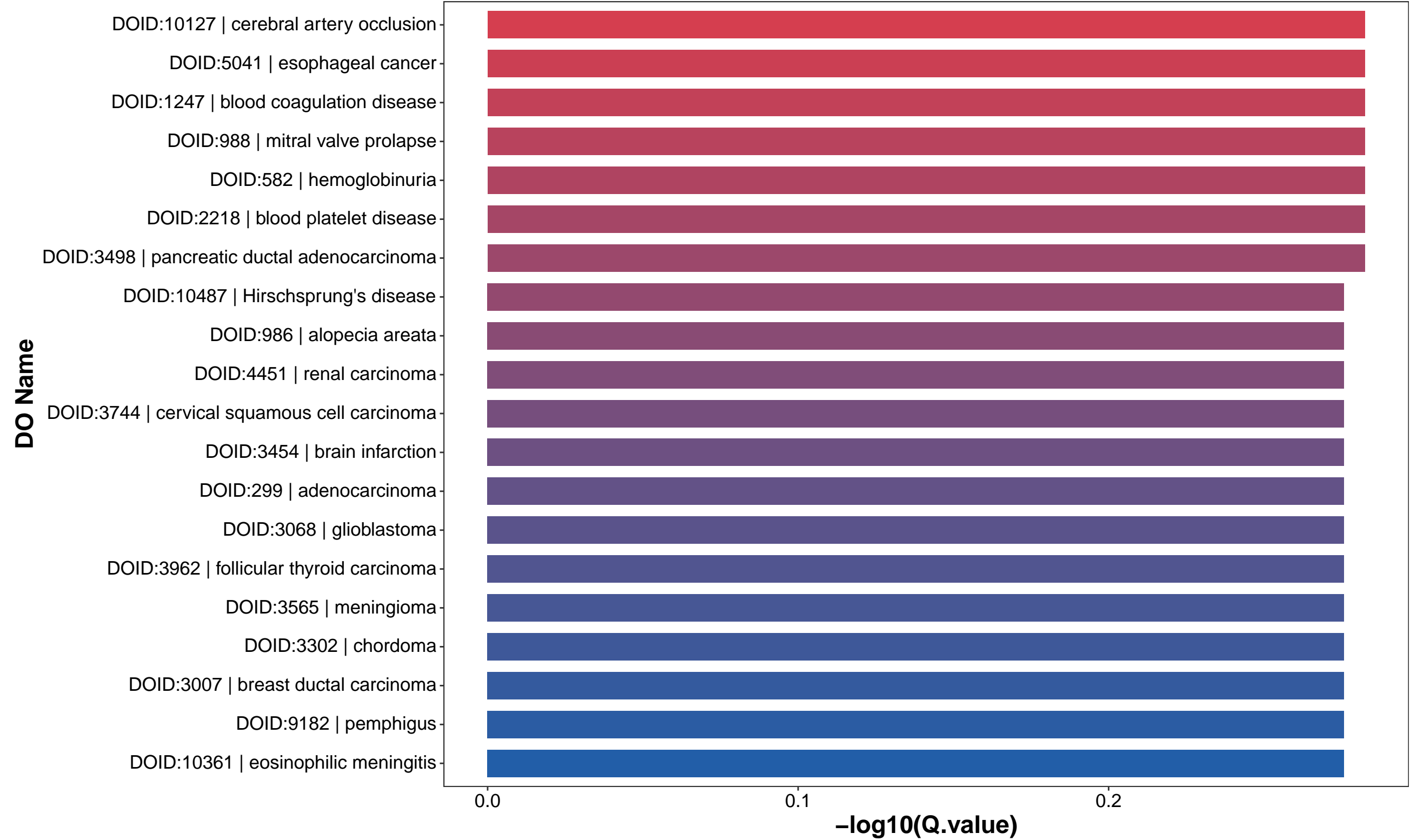

Supplement: Supplementary file 1 [file DataSheet1.zip › summary of proteomics/summary/04.Diff_analysis/COND1/FHVSZH/Enrichment/DO/COND1.FHVSZH.DO_Enrichment.Q.pdf]

# DO Enrichment ScatterPlot

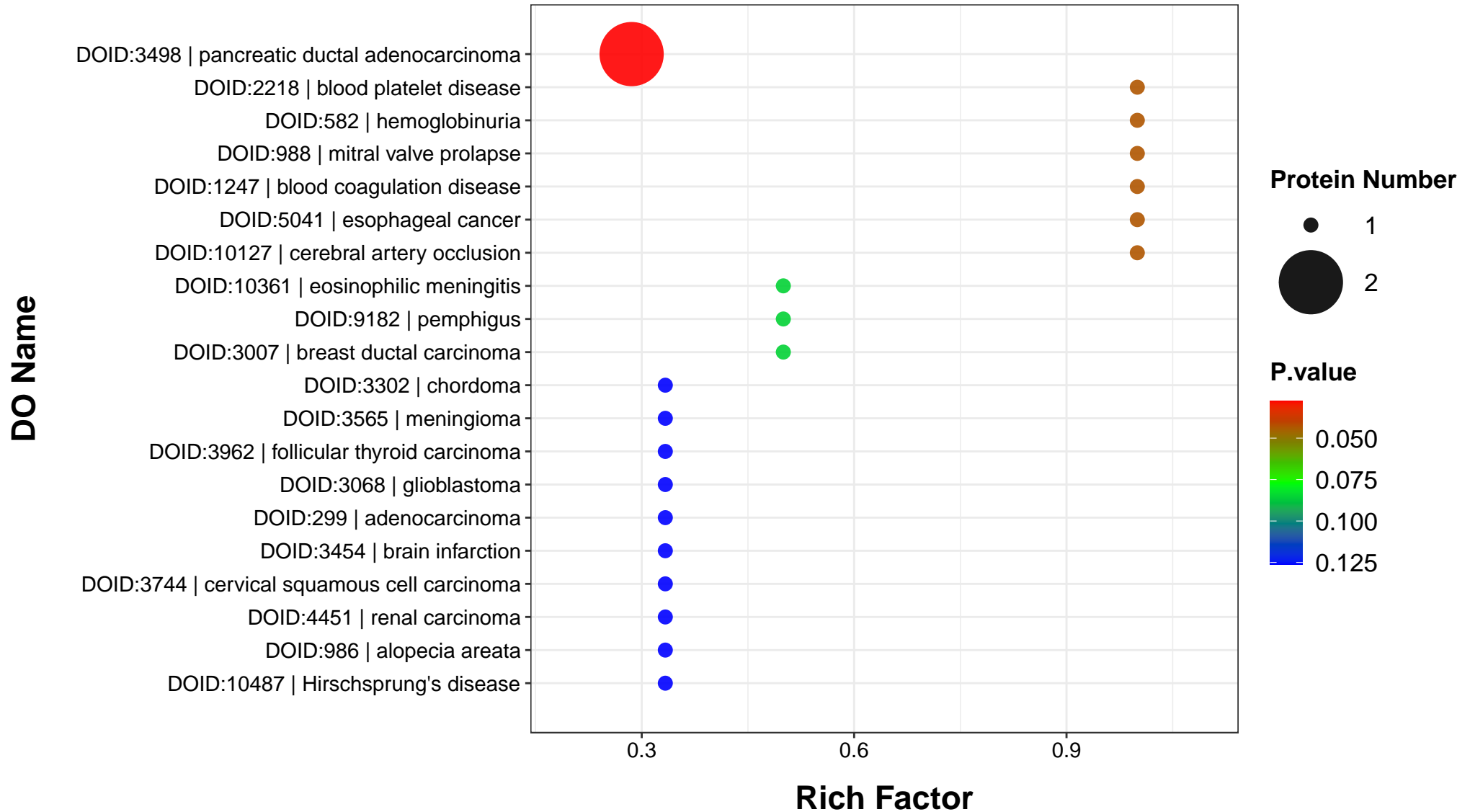

Supplement: Supplementary file 1 [file DataSheet1.zip › summary of proteomics/summary/04.Diff_analysis/COND1/FHVSZH/Enrichment/DO/COND1.FHVSZH.DO_scatter.pdf]

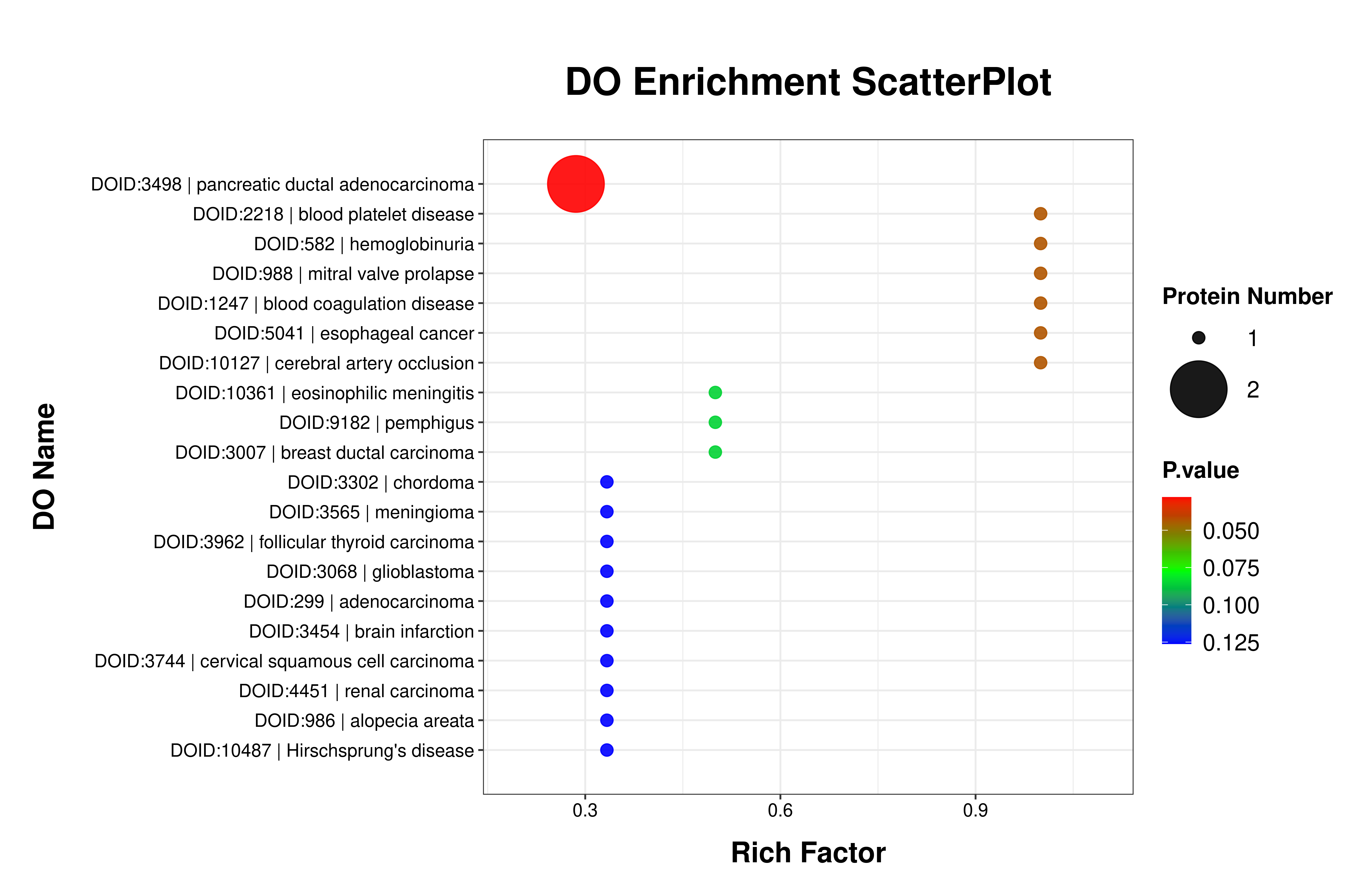

Supplement: Supplementary file 1 [file DataSheet1.zip › summary of proteomics/summary/04.Diff_analysis/COND1/FHVSZH/Enrichment/DO/COND1.FHVSZH.DO_scatter.png]

# GO Enrichment BarPlot

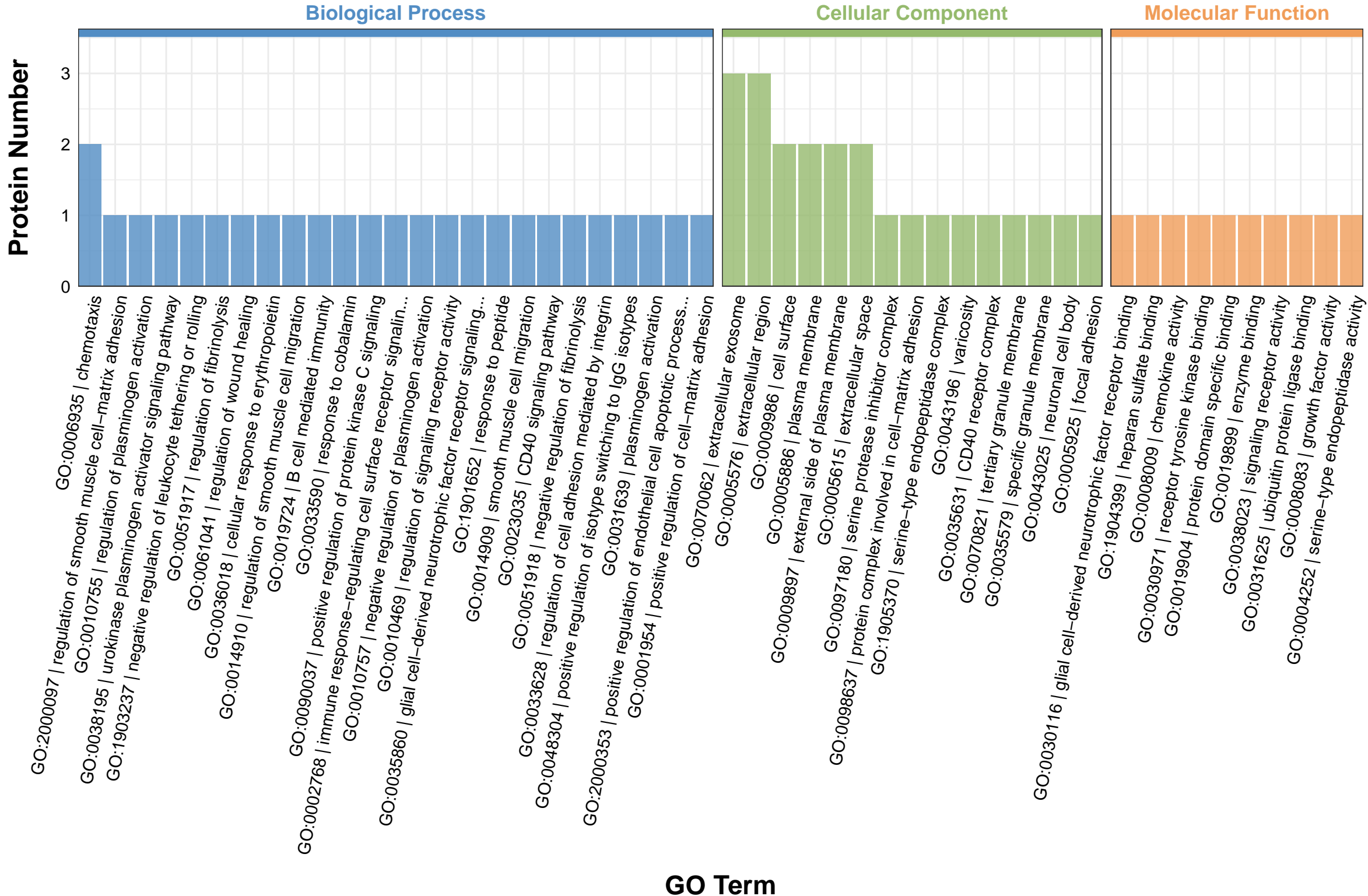

Supplement: Supplementary file 1 [file DataSheet1.zip › summary of proteomics/summary/04.Diff_analysis/COND1/FHVSZH/Enrichment/GO/result.FHVSZH.GO_BarPlot_GeneNumber.pdf]

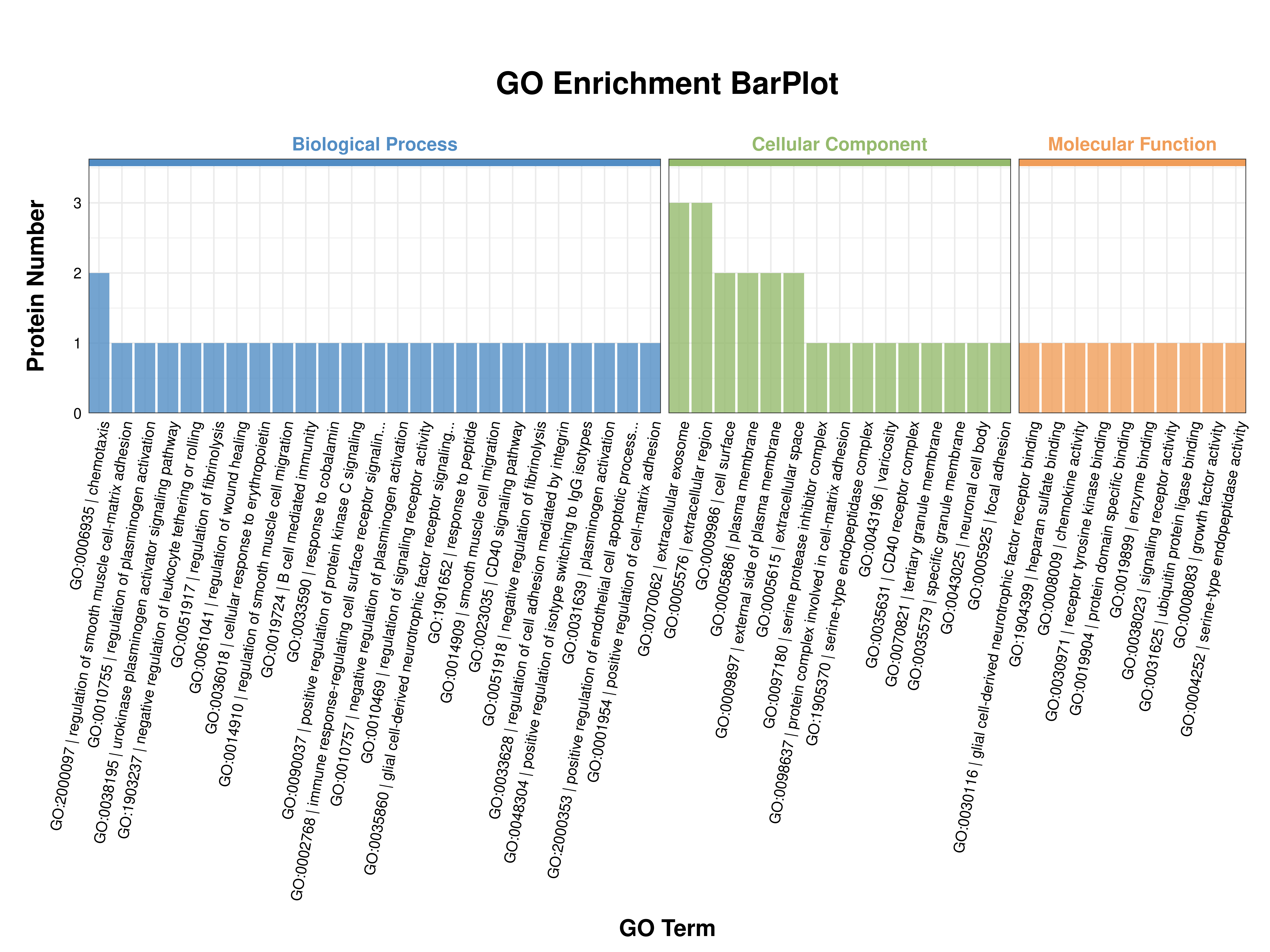

Supplement: Supplementary file 1 [file DataSheet1.zip › summary of proteomics/summary/04.Diff_analysis/COND1/FHVSZH/Enrichment/GO/result.FHVSZH.GO_BarPlot_GeneNumber.png]

# GO Enrichment BarPlot

GO Term

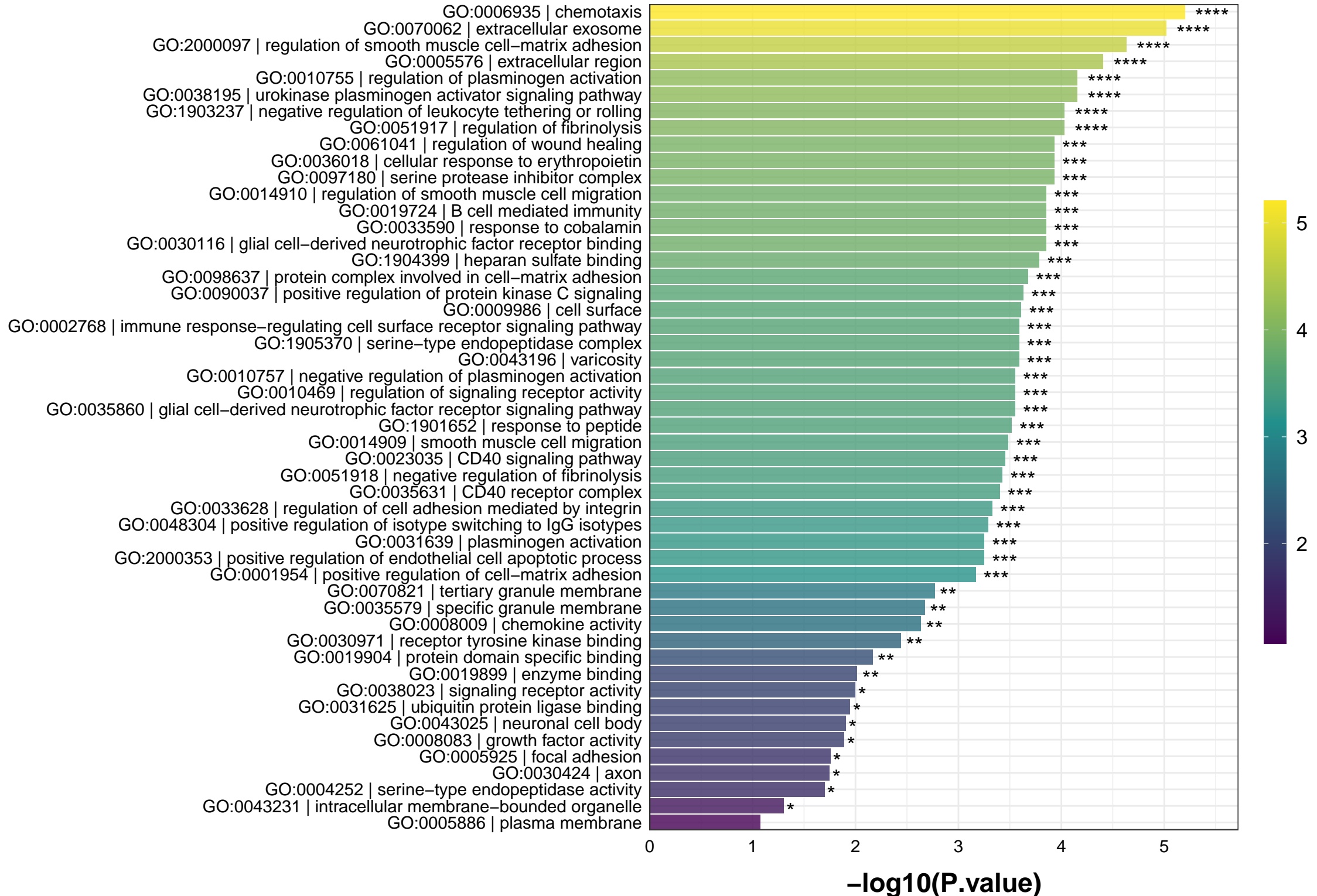

Supplement: Supplementary file 1 [file DataSheet1.zip › summary of proteomics/summary/04.Diff_analysis/COND1/FHVSZH/Enrichment/GO/result.FHVSZH.GO_BarPlot_Sig.P.pdf]

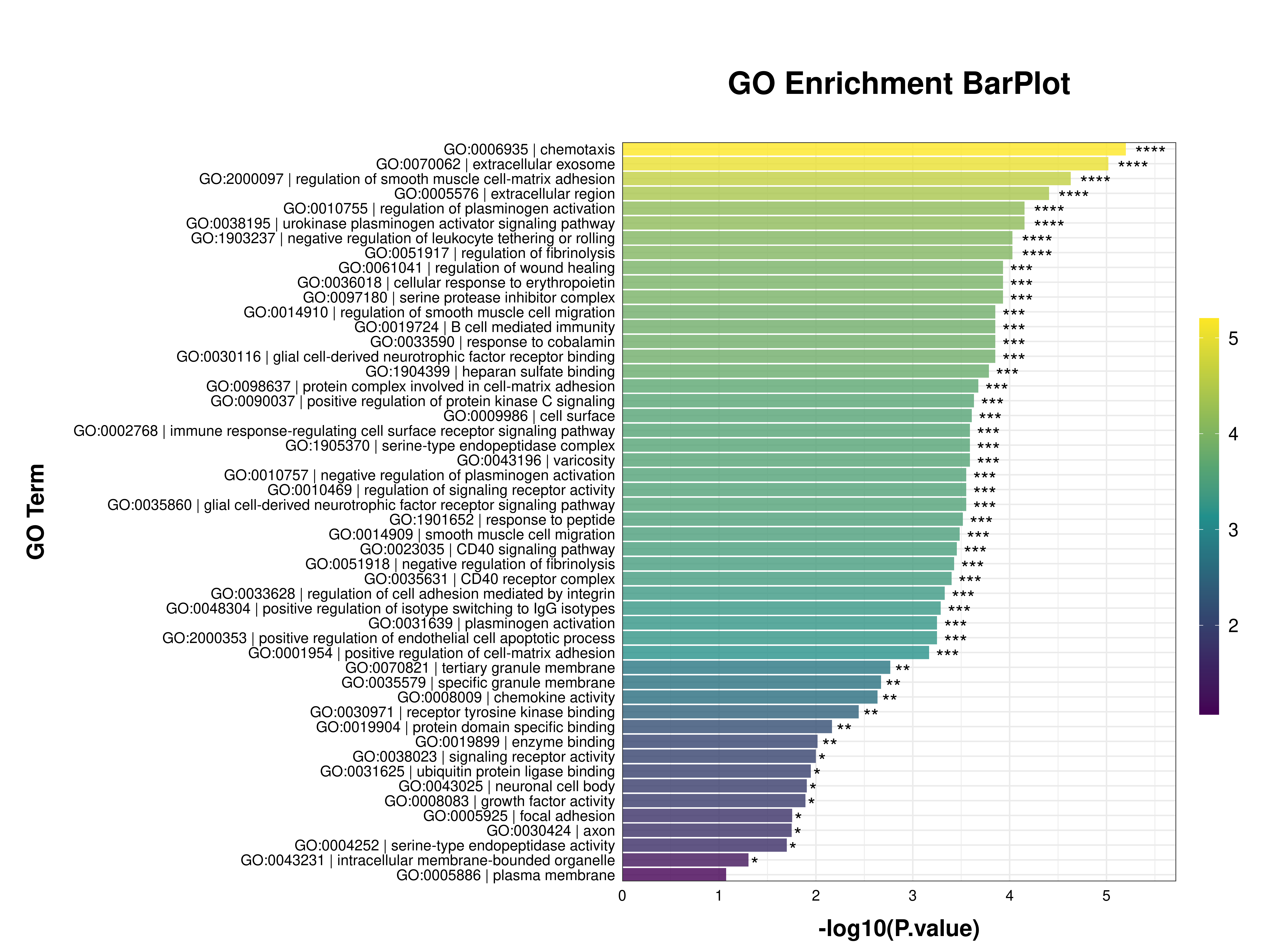

Supplement: Supplementary file 1 [file DataSheet1.zip › summary of proteomics/summary/04.Diff_analysis/COND1/FHVSZH/Enrichment/GO/result.FHVSZH.GO_BarPlot_Sig.P.png]

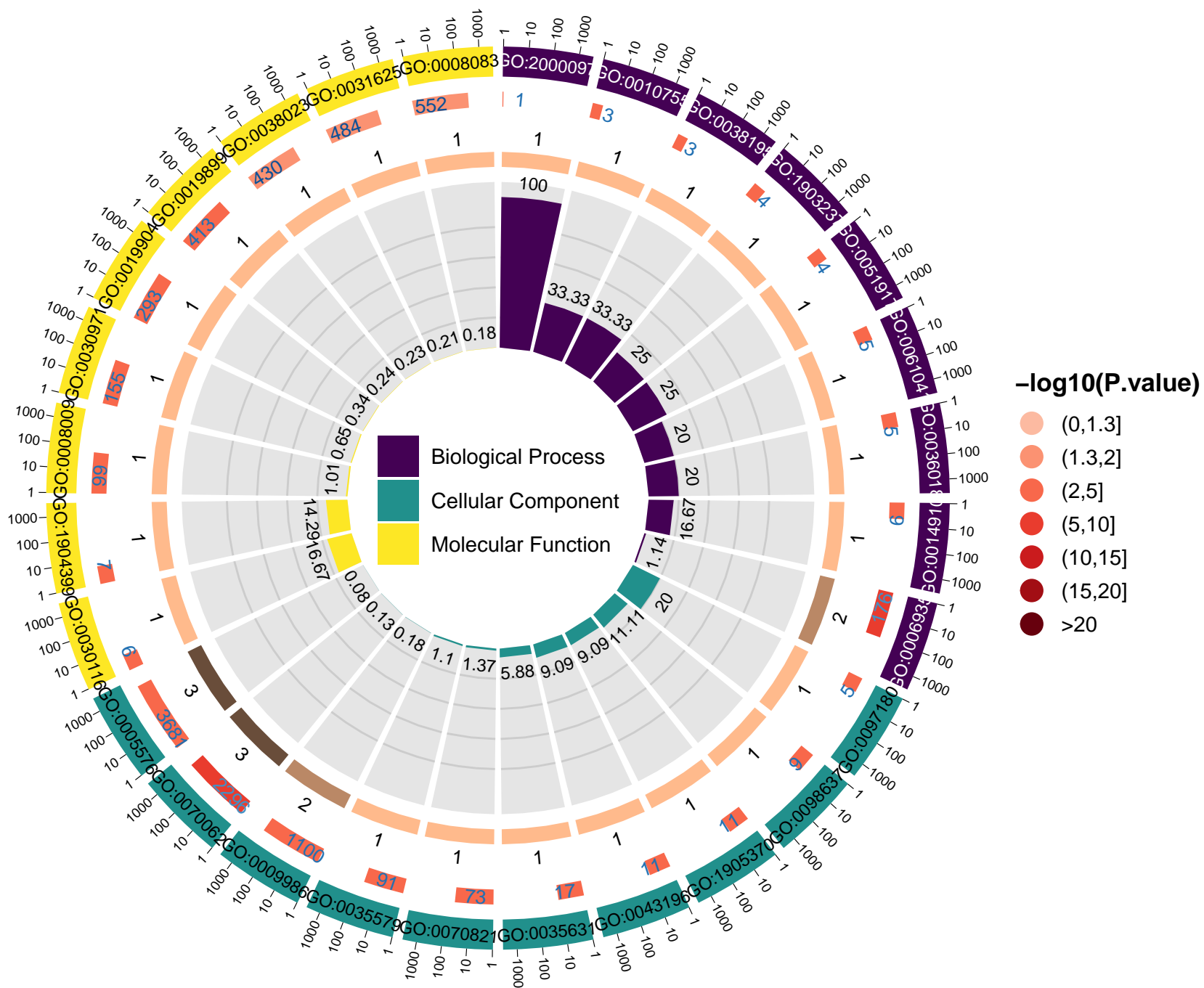

Supplement: Supplementary file 1 [file DataSheet1.zip › summary of proteomics/summary/04.Diff_analysis/COND1/FHVSZH/Enrichment/GO/result.FHVSZH.GO_LoopCircos.P.pdf]

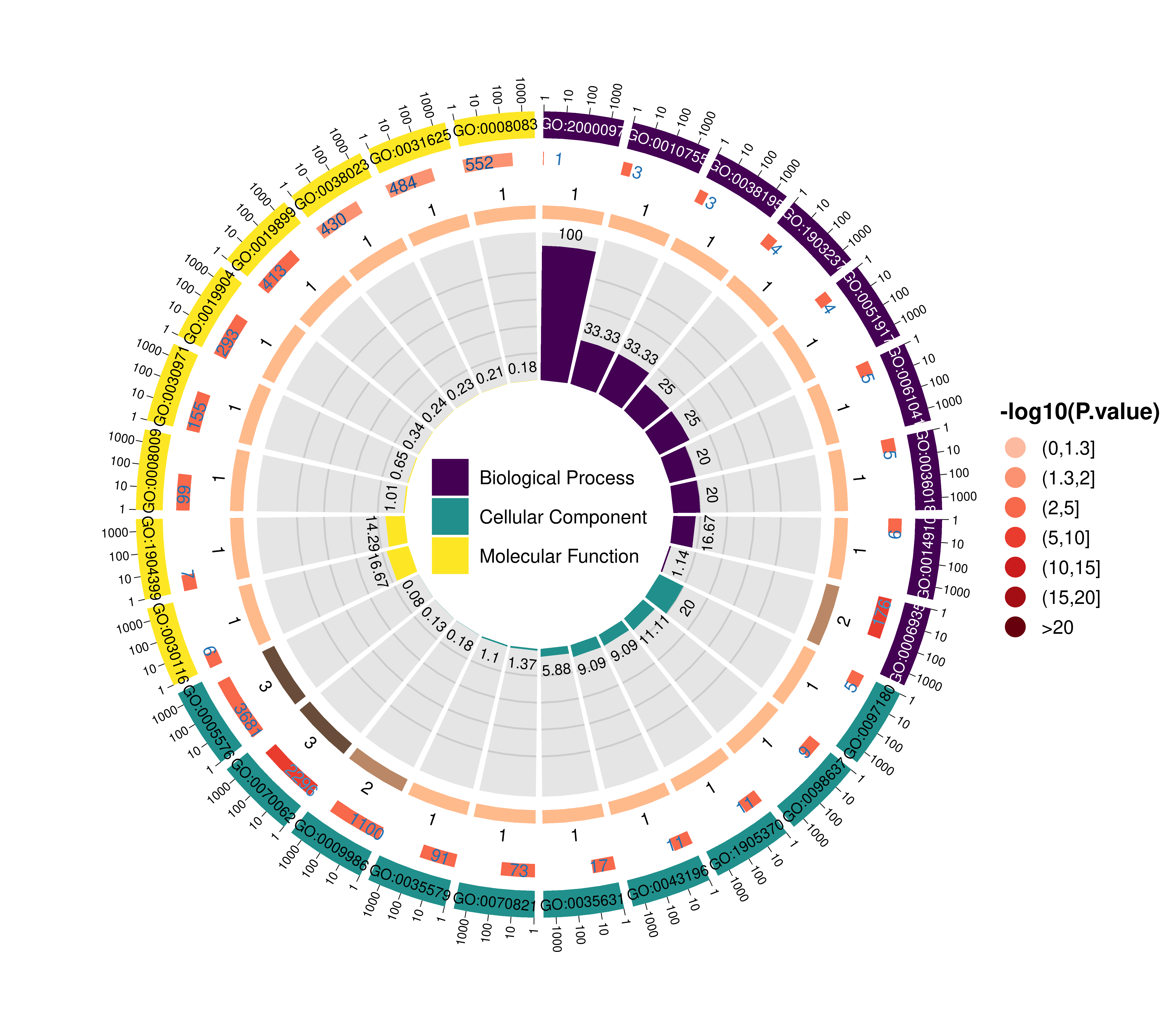

Supplement: Supplementary file 1 [file DataSheet1.zip › summary of proteomics/summary/04.Diff_analysis/COND1/FHVSZH/Enrichment/GO/result.FHVSZH.GO_LoopCircos.P.png]

# GO Enrichment ScatterPlot

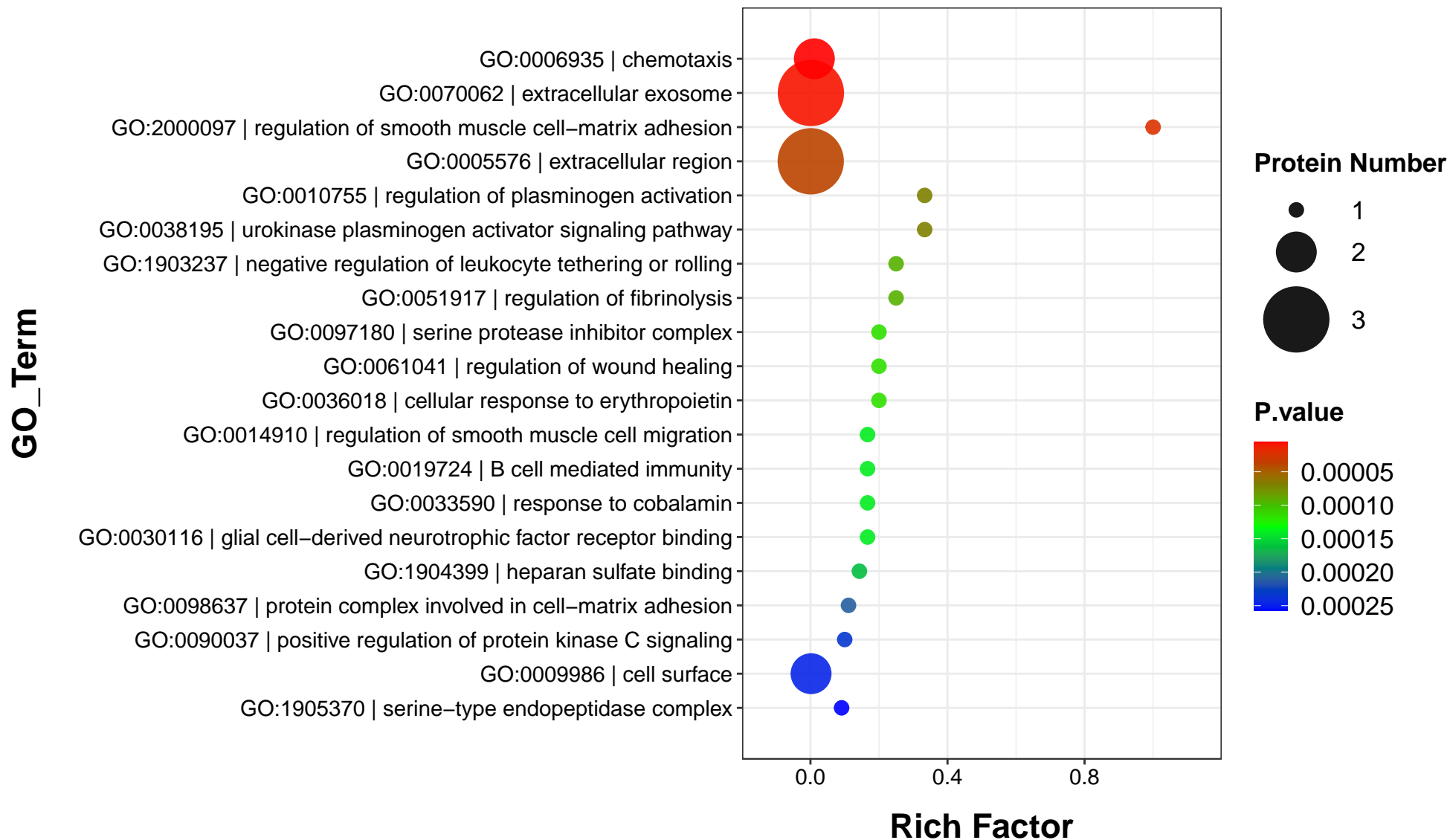

Supplement: Supplementary file 1 [file DataSheet1.zip › summary of proteomics/summary/04.Diff_analysis/COND1/FHVSZH/Enrichment/GO/result.FHVSZH.GO_ScatterPlot.P.pdf]

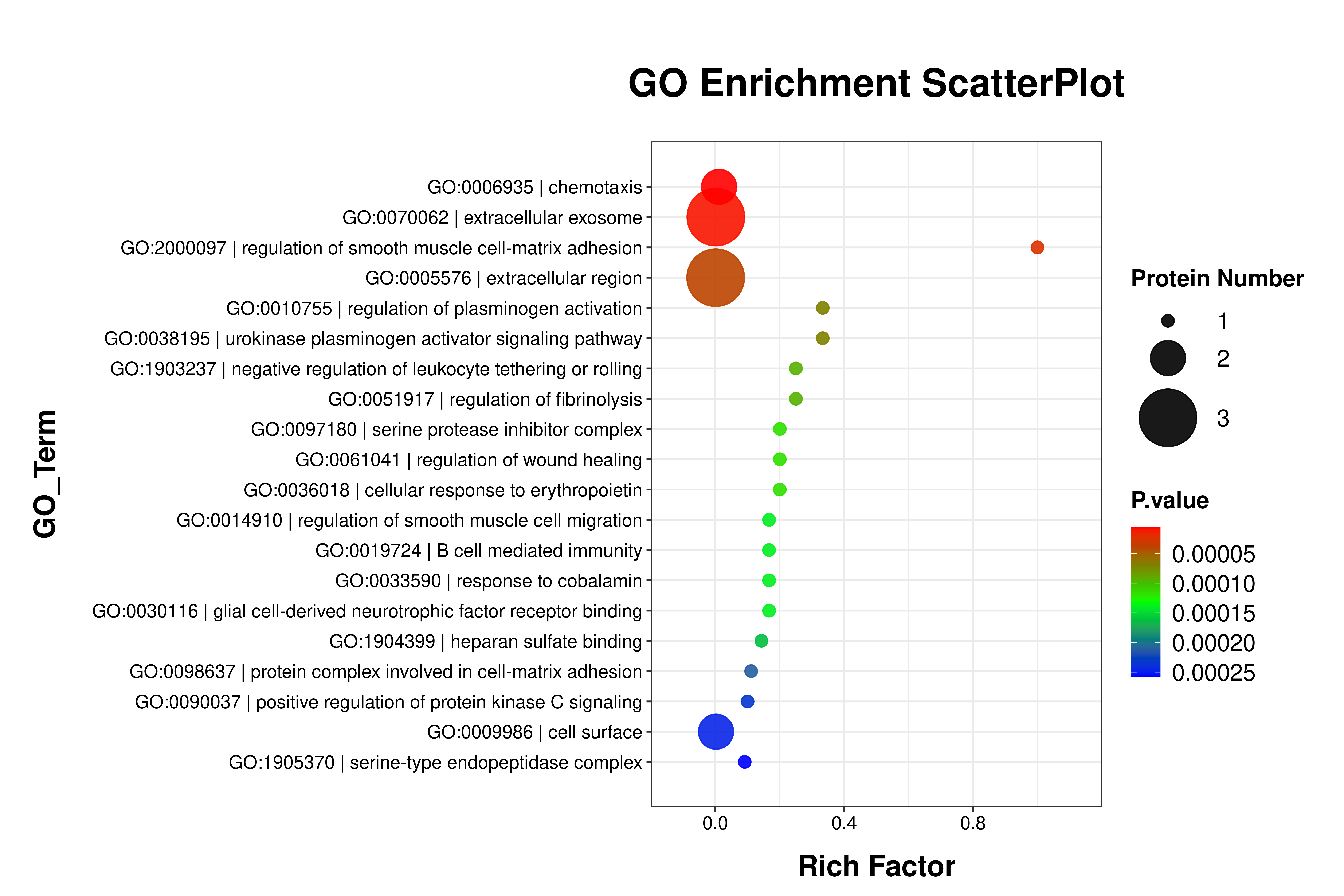

Supplement: Supplementary file 1 [file DataSheet1.zip › summary of proteomics/summary/04.Diff_analysis/COND1/FHVSZH/Enrichment/GO/result.FHVSZH.GO_ScatterPlot.P.png]

# Top 20 of InterPro Enrichment

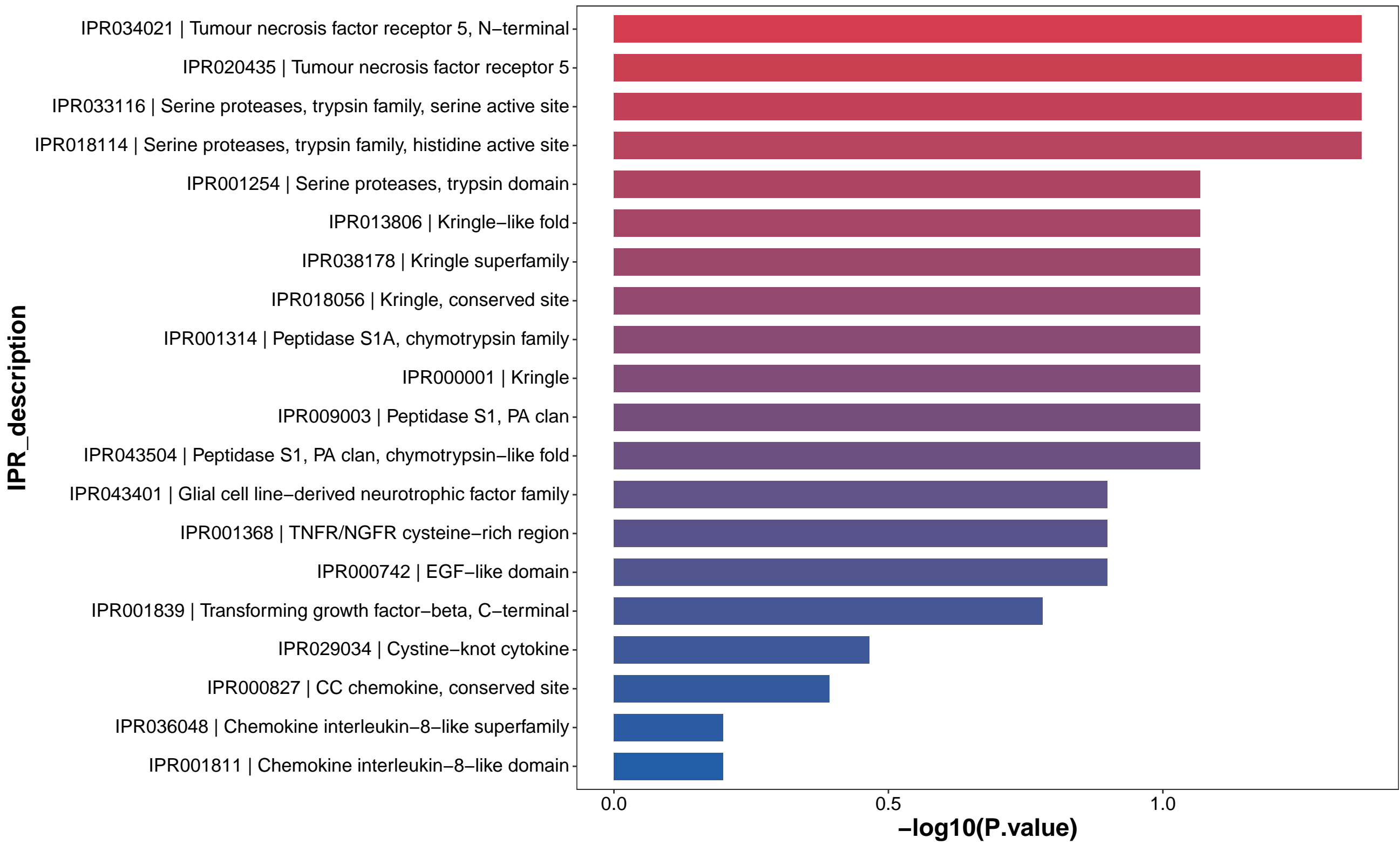

Supplement: Supplementary file 1 [file DataSheet1.zip › summary of proteomics/summary/04.Diff_analysis/COND1/FHVSZH/Enrichment/InterPro/COND1.FHVSZH.InterPro_Enrichment.P.pdf]

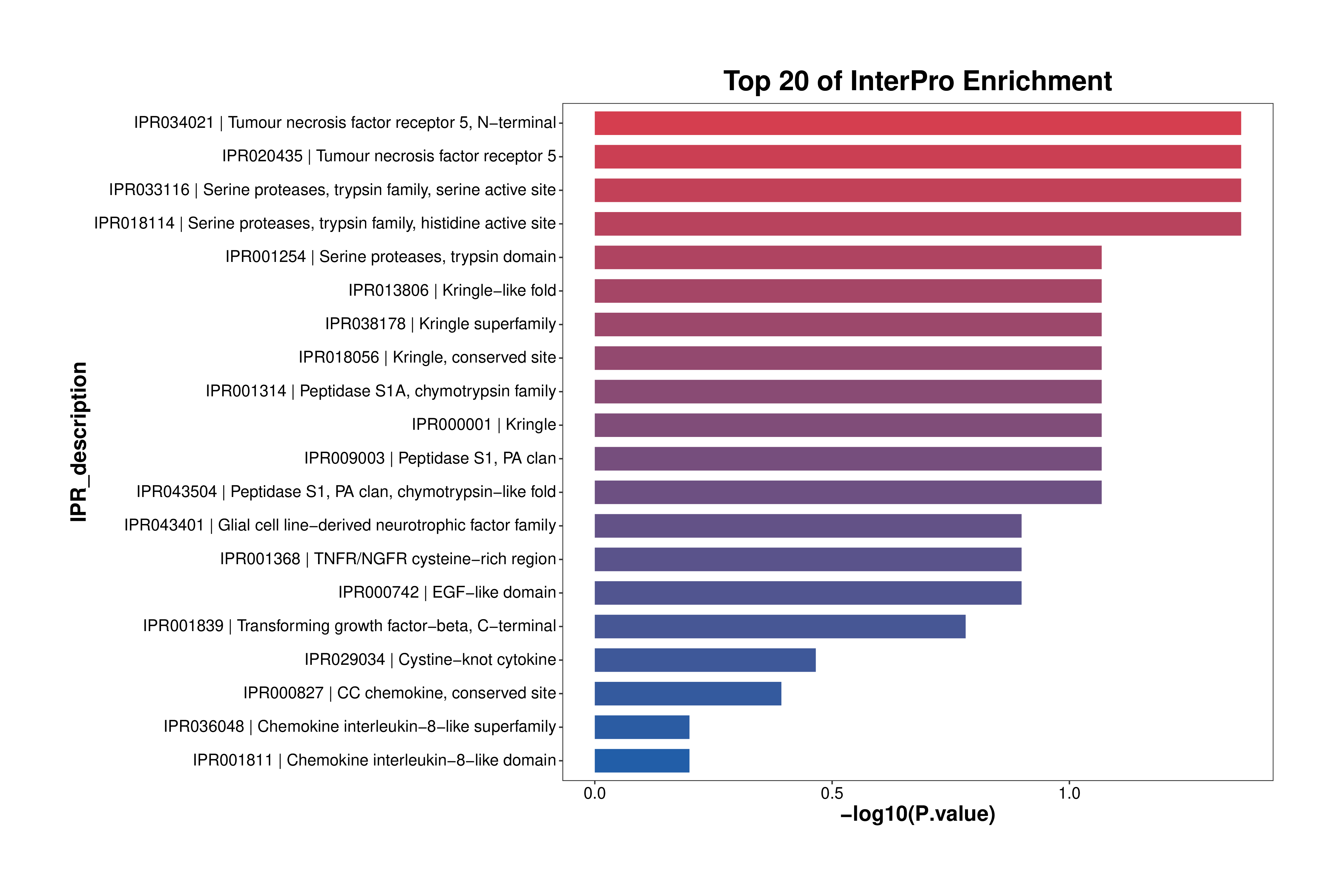

Supplement: Supplementary file 1 [file DataSheet1.zip › summary of proteomics/summary/04.Diff_analysis/COND1/FHVSZH/Enrichment/InterPro/COND1.FHVSZH.InterPro_Enrichment.P.png]

# Top 20 of InterPro Enrichment

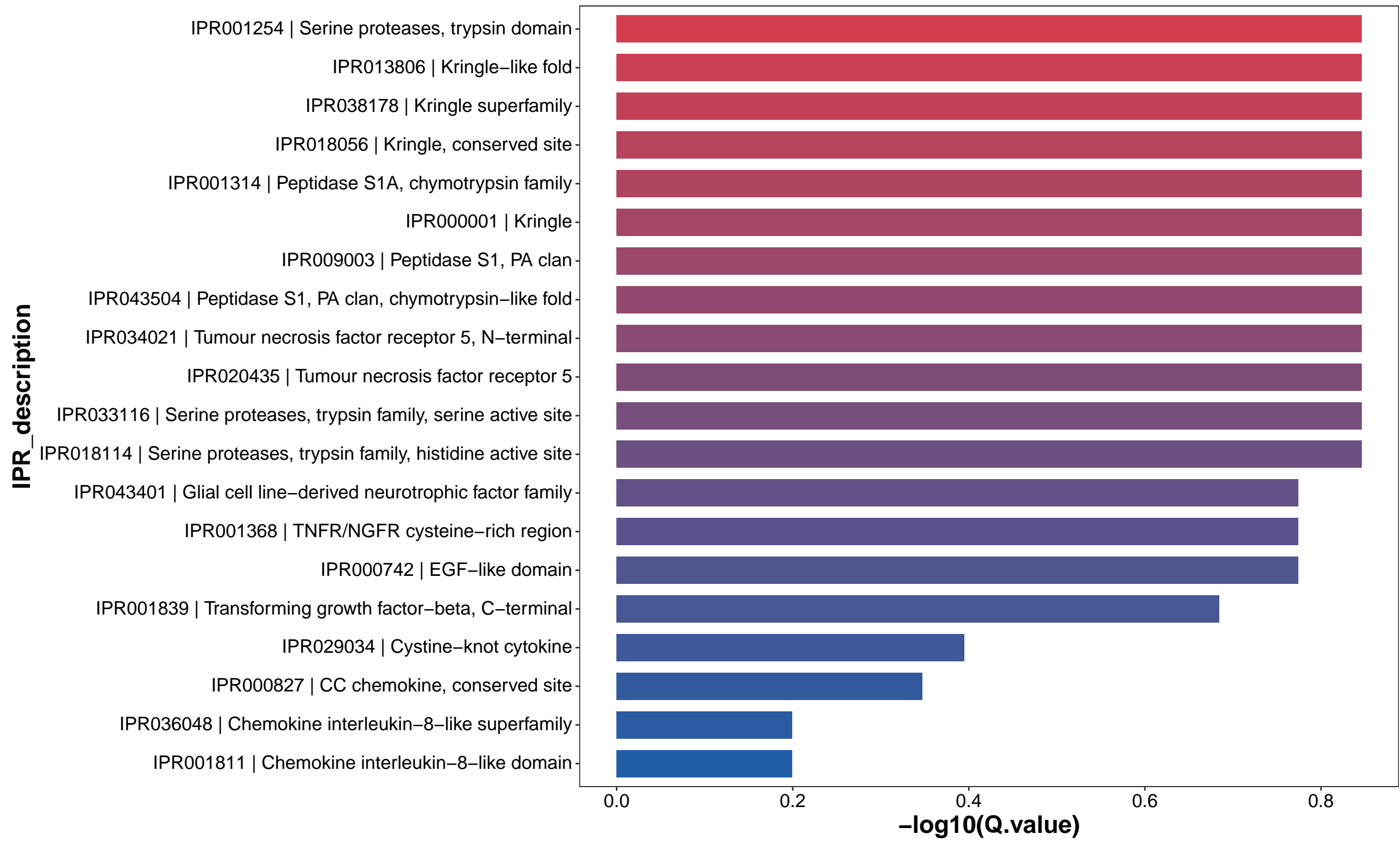

Supplement: Supplementary file 1 [file DataSheet1.zip › summary of proteomics/summary/04.Diff_analysis/COND1/FHVSZH/Enrichment/InterPro/COND1.FHVSZH.InterPro_Enrichment.Q.pdf]

# InterPro Enrichment ScatterPlot

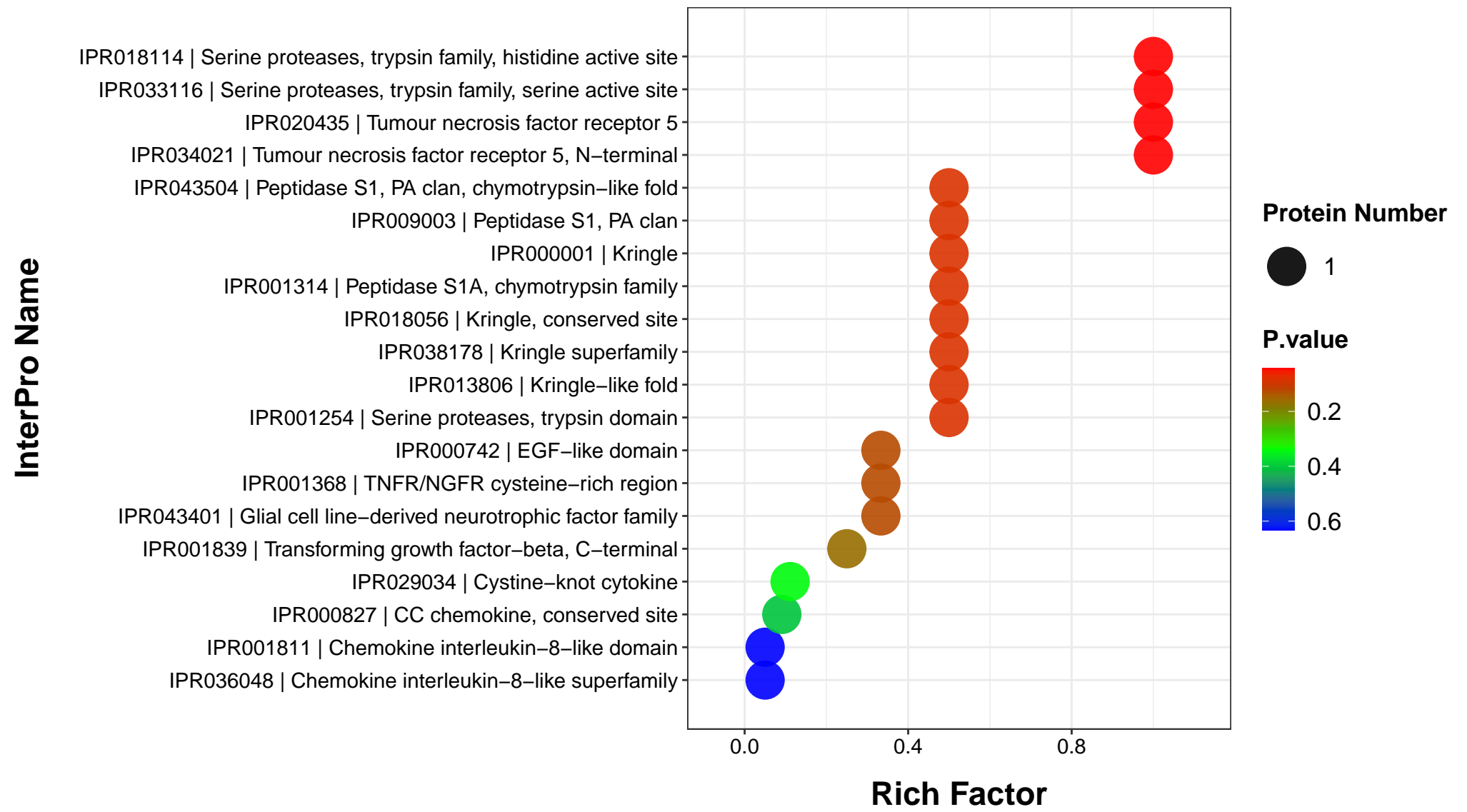

Supplement: Supplementary file 1 [file DataSheet1.zip › summary of proteomics/summary/04.Diff_analysis/COND1/FHVSZH/Enrichment/InterPro/COND1.FHVSZH.InterPro_scatter.pdf]

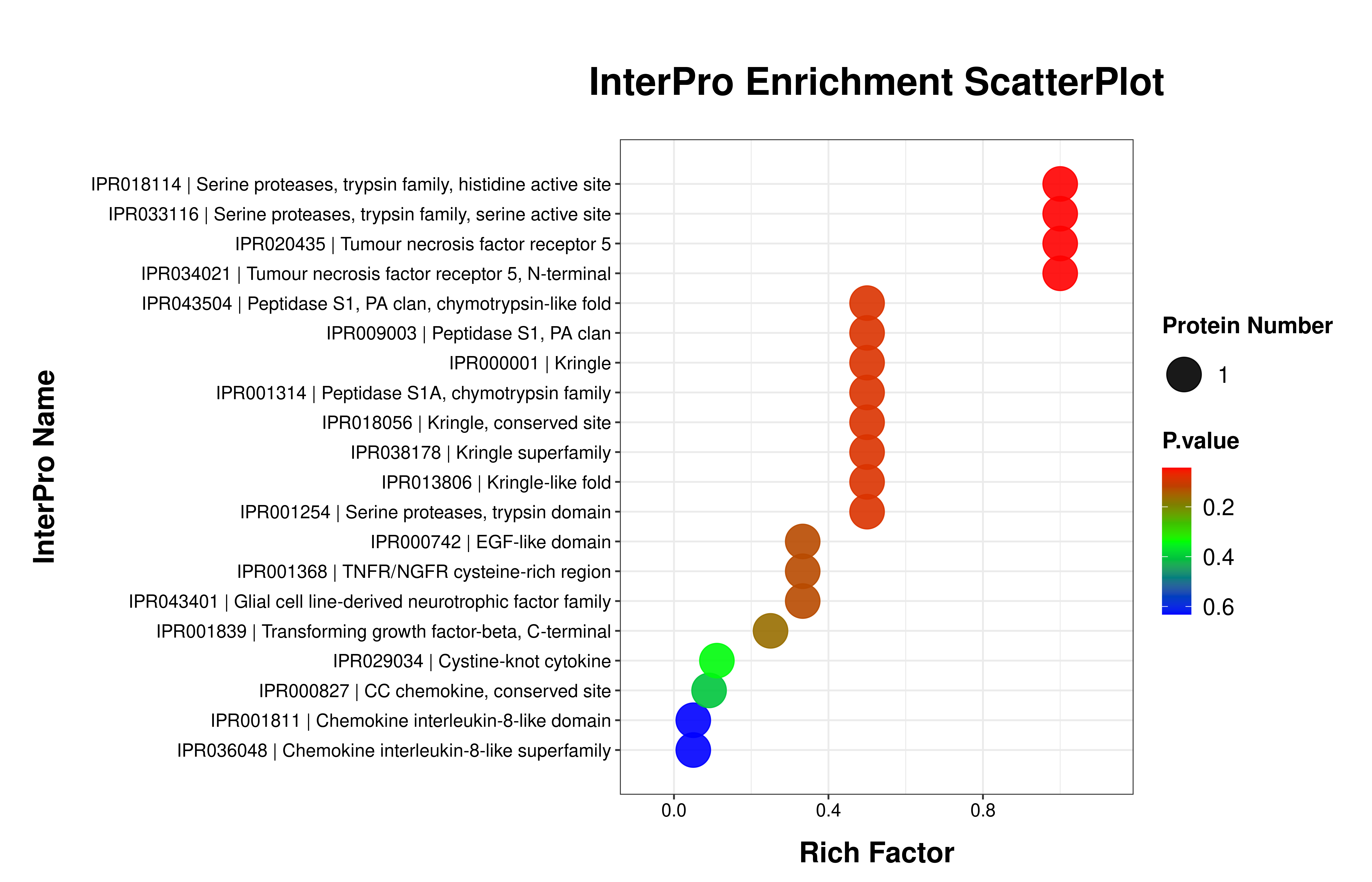

Supplement: Supplementary file 1 [file DataSheet1.zip › summary of proteomics/summary/04.Diff_analysis/COND1/FHVSZH/Enrichment/InterPro/COND1.FHVSZH.InterPro_scatter.png]

# KEGG Enrichment BarPlot

Pathway Name

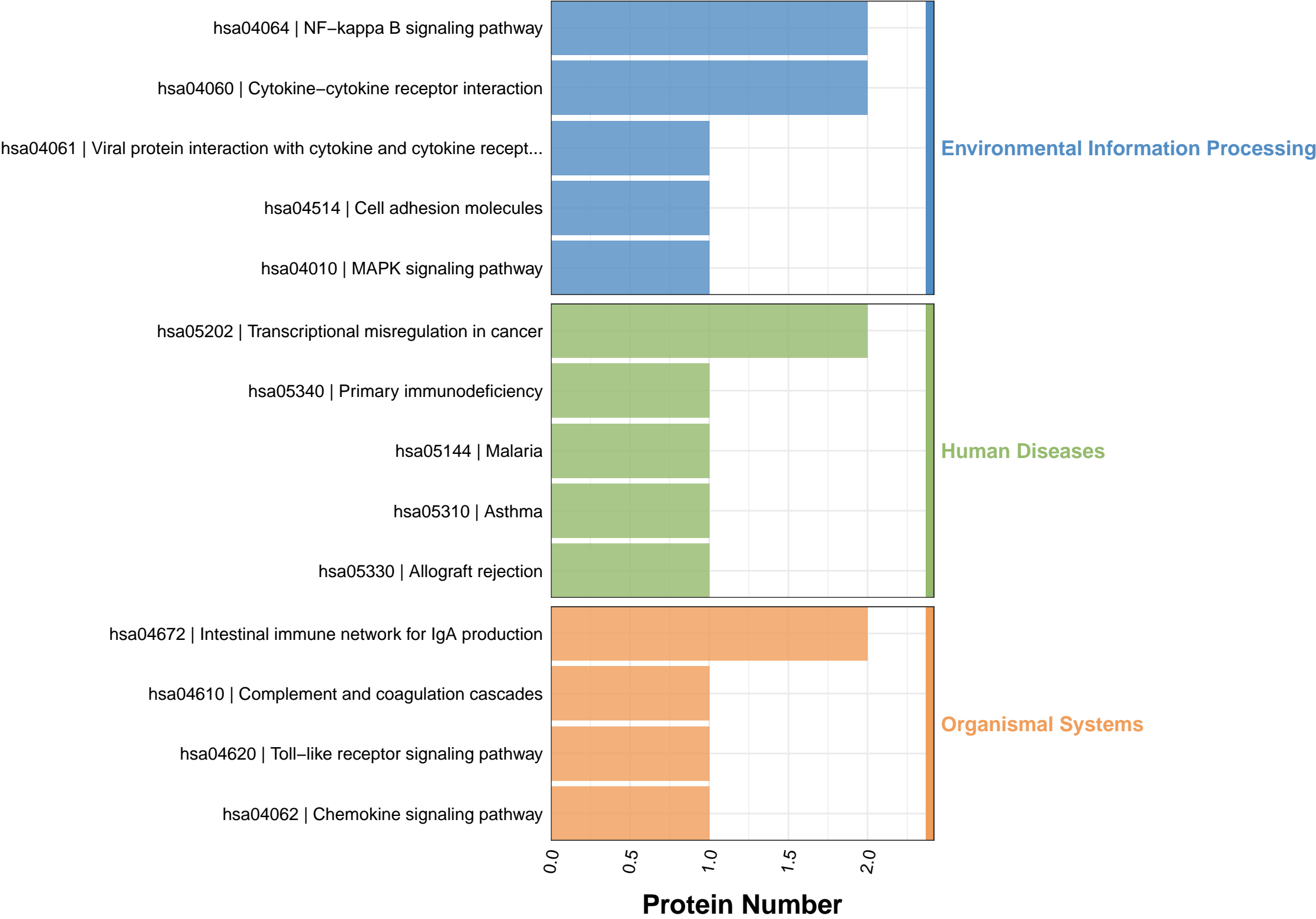

Supplement: Supplementary file 1 [file DataSheet1.zip › summary of proteomics/summary/04.Diff_analysis/COND1/FHVSZH/Enrichment/KEGG/result.FHVSZH.KEGG_BarPlot_GeneNumber.pdf]

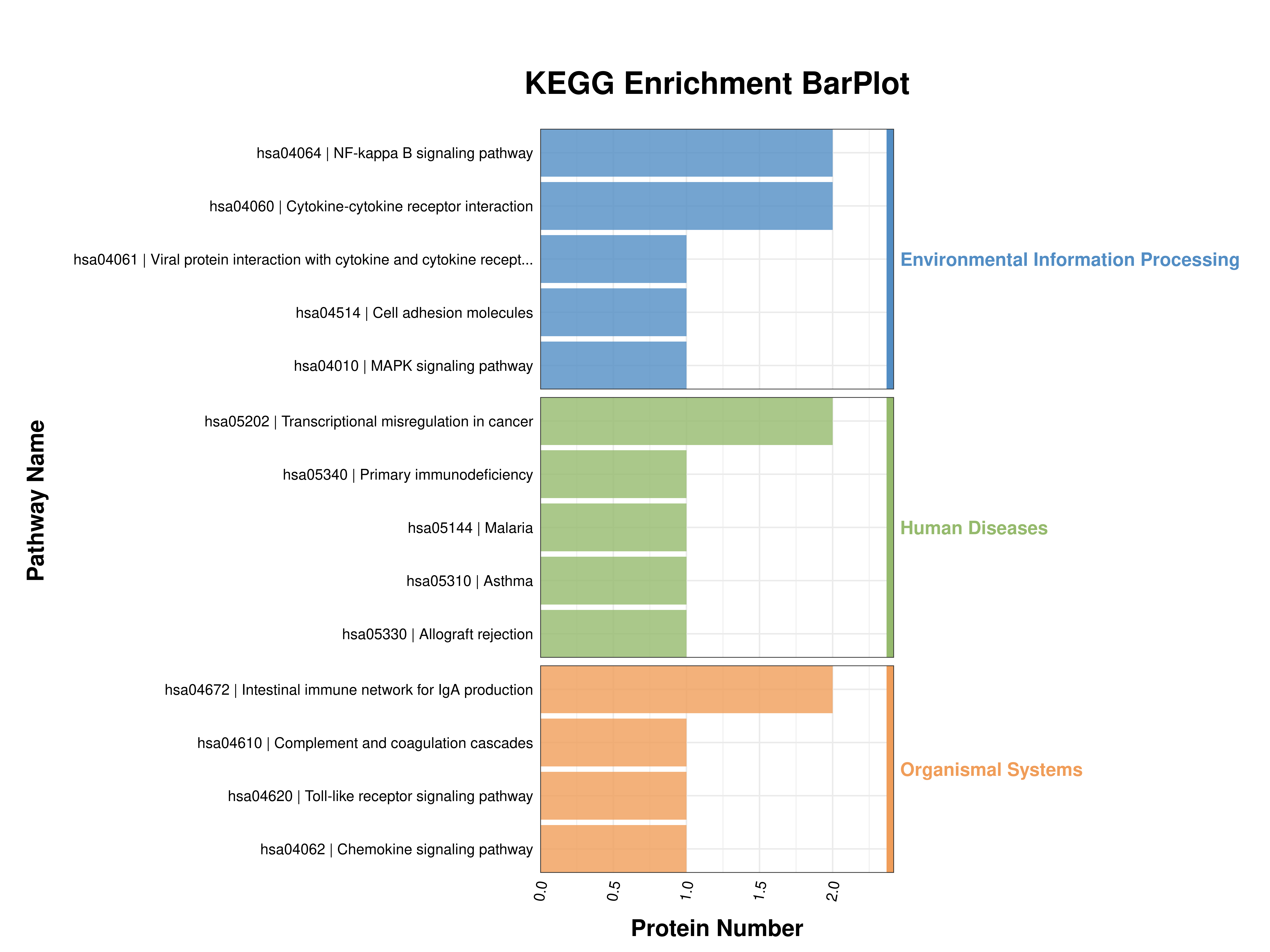

Supplement: Supplementary file 1 [file DataSheet1.zip › summary of proteomics/summary/04.Diff_analysis/COND1/FHVSZH/Enrichment/KEGG/result.FHVSZH.KEGG_BarPlot_GeneNumber.png]

# KEGG Enrichment BarPlot

Pathway Name

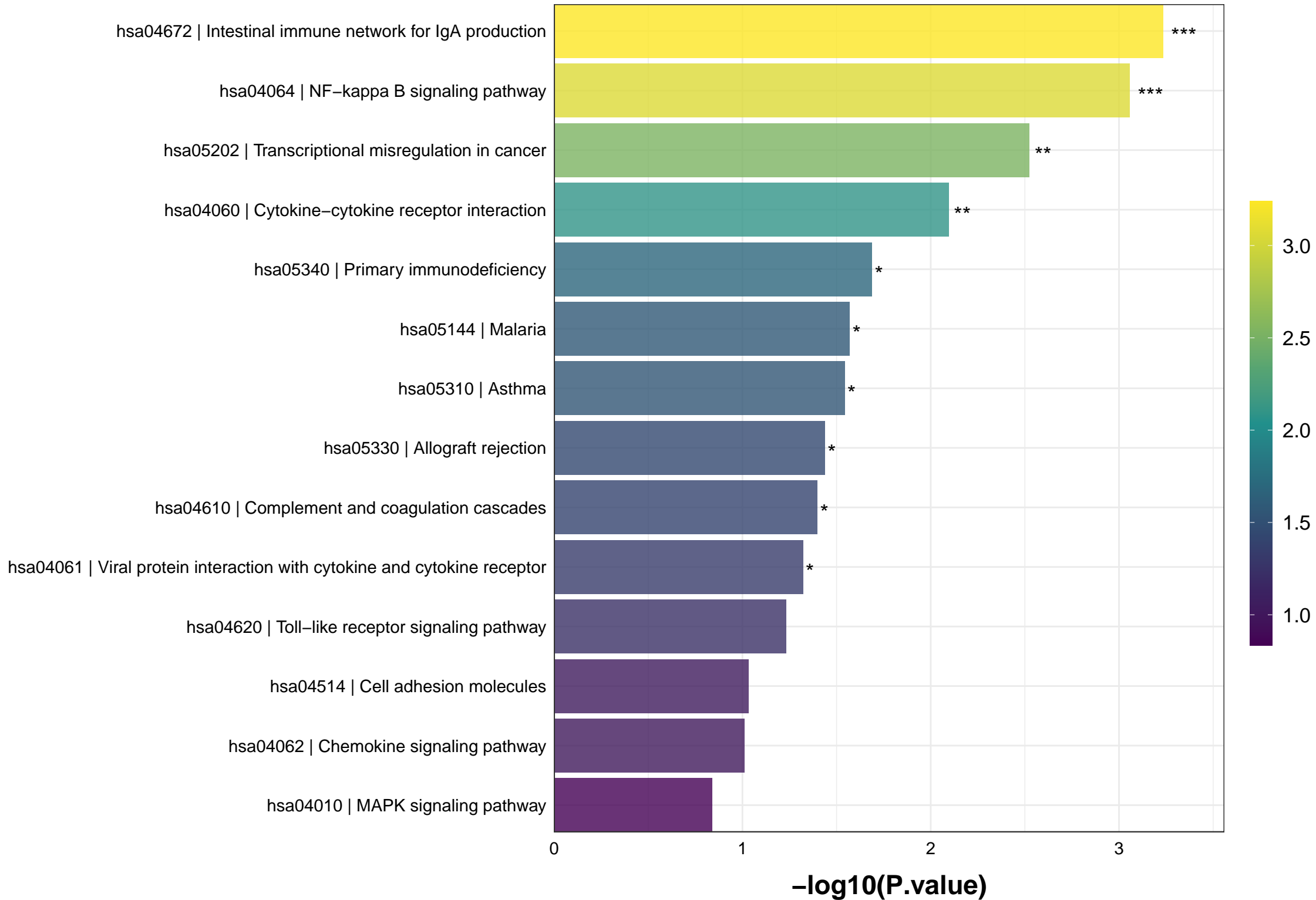

Supplement: Supplementary file 1 [file DataSheet1.zip › summary of proteomics/summary/04.Diff_analysis/COND1/FHVSZH/Enrichment/KEGG/result.FHVSZH.KEGG_BarPlot_Sig.P.pdf]

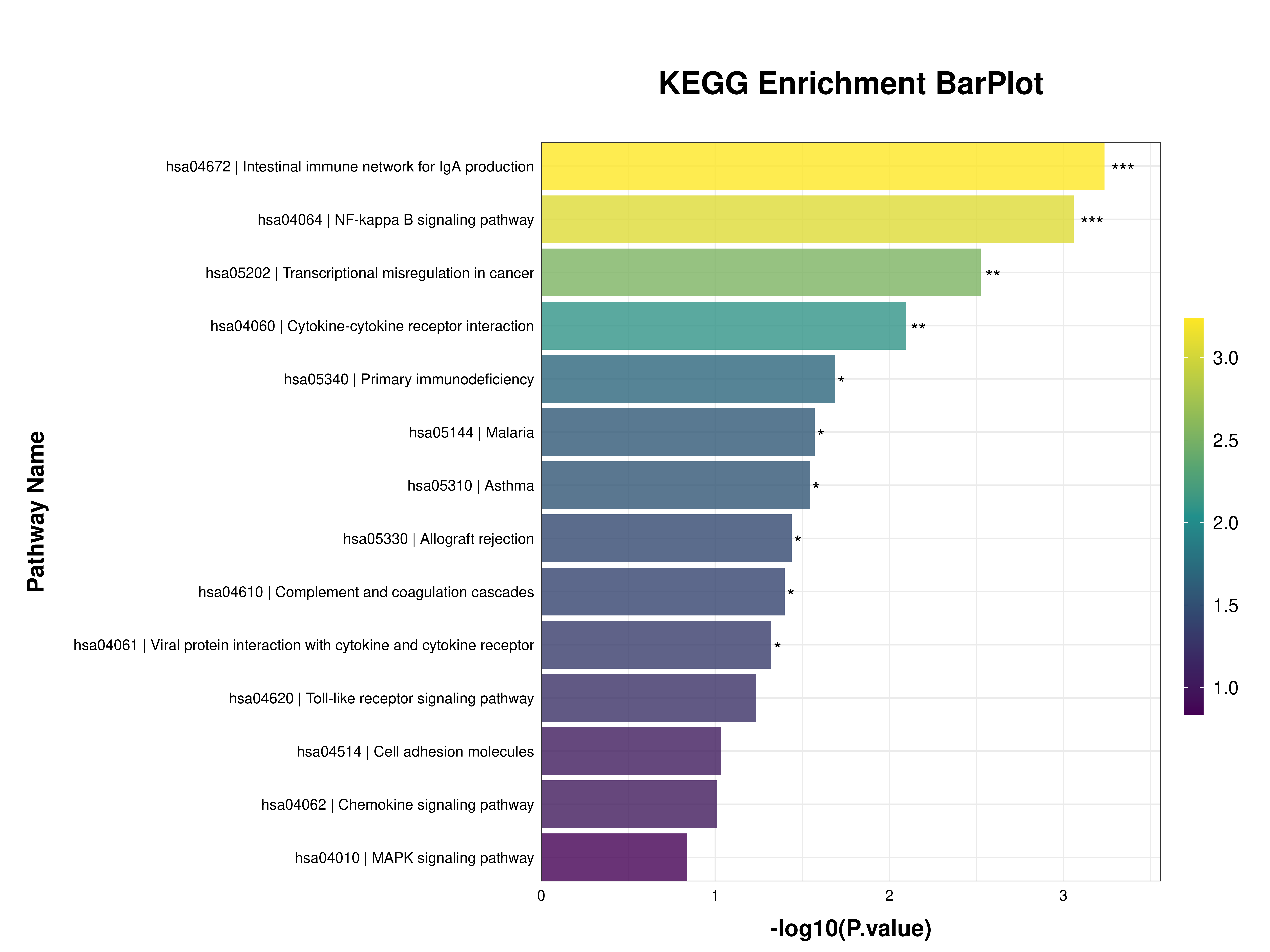

Supplement: Supplementary file 1 [file DataSheet1.zip › summary of proteomics/summary/04.Diff_analysis/COND1/FHVSZH/Enrichment/KEGG/result.FHVSZH.KEGG_BarPlot_Sig.P.png]

# KEGG Enrichment ScatterPlot

Pathway

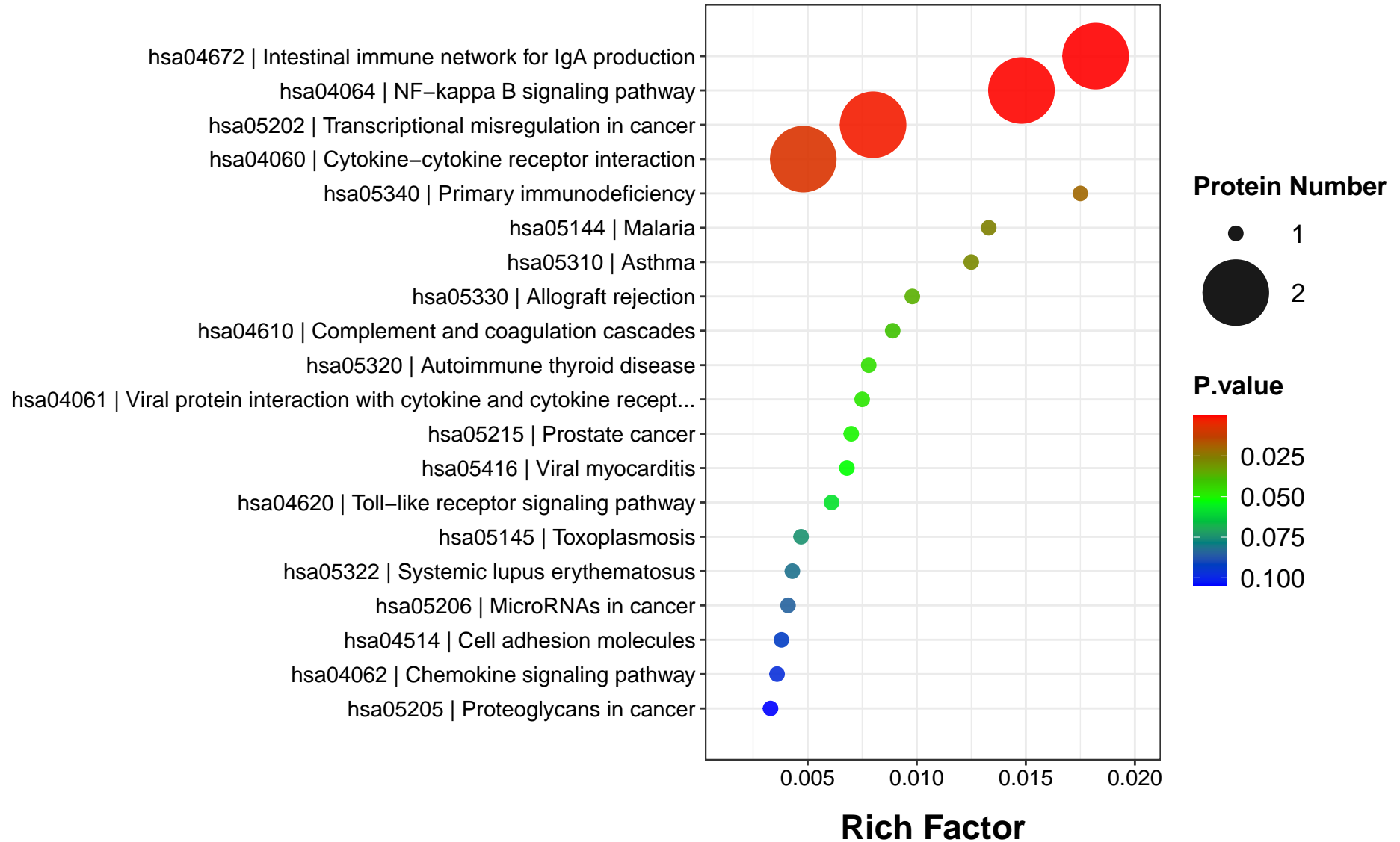

Supplement: Supplementary file 1 [file DataSheet1.zip › summary of proteomics/summary/04.Diff_analysis/COND1/FHVSZH/Enrichment/KEGG/result.FHVSZH.KEGG_ScatterPlot.P.pdf]

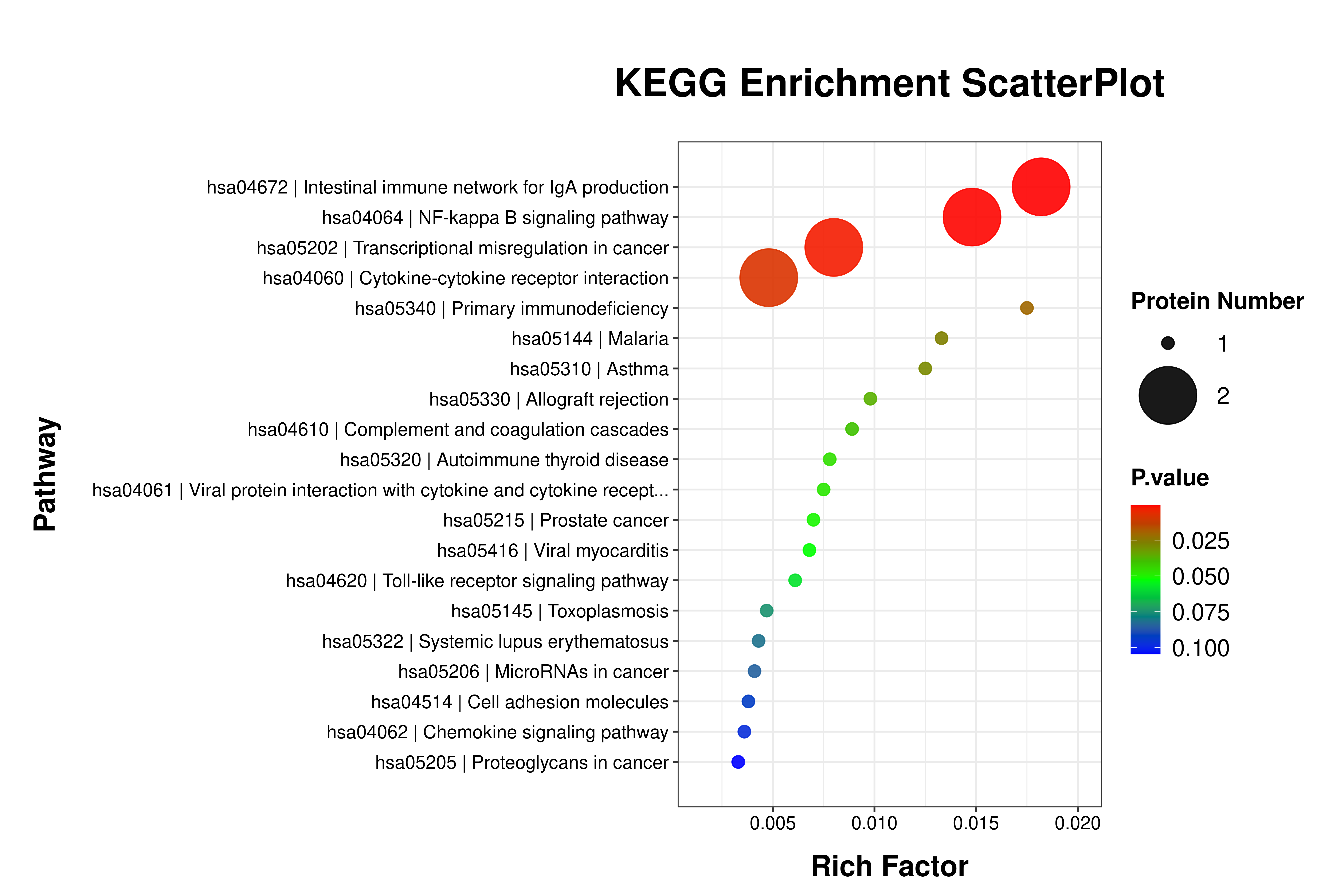

Supplement: Supplementary file 1 [file DataSheet1.zip › summary of proteomics/summary/04.Diff_analysis/COND1/FHVSZH/Enrichment/KEGG/result.FHVSZH.KEGG_ScatterPlot.P.png]

# KEGG Enrichment BubblePlot

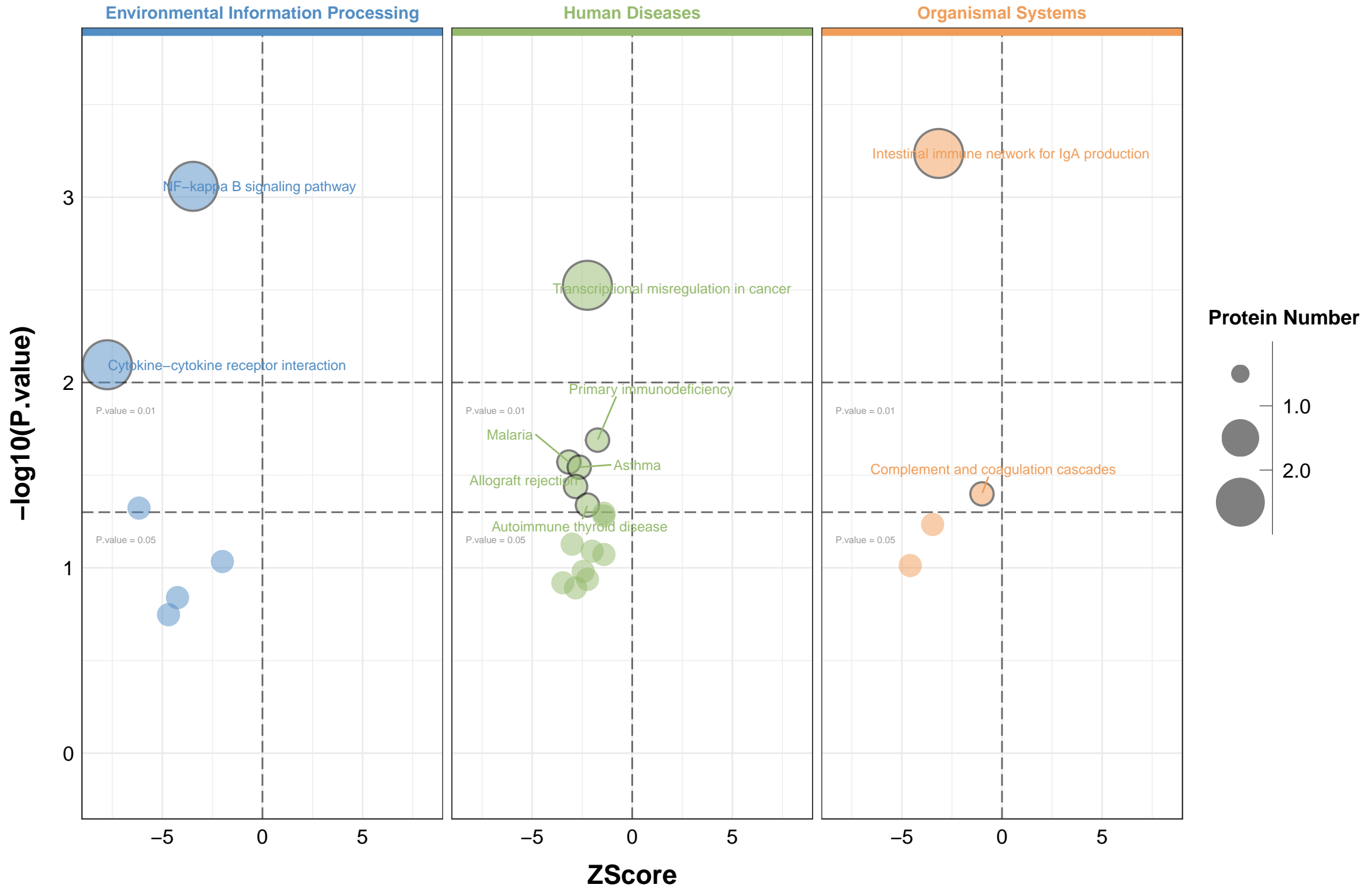

Supplement: Supplementary file 1 [file DataSheet1.zip › summary of proteomics/summary/04.Diff_analysis/COND1/FHVSZH/Enrichment/KEGG/result.FHVSZH.KEGG_ZScore_BubblePlot.P.pdf]

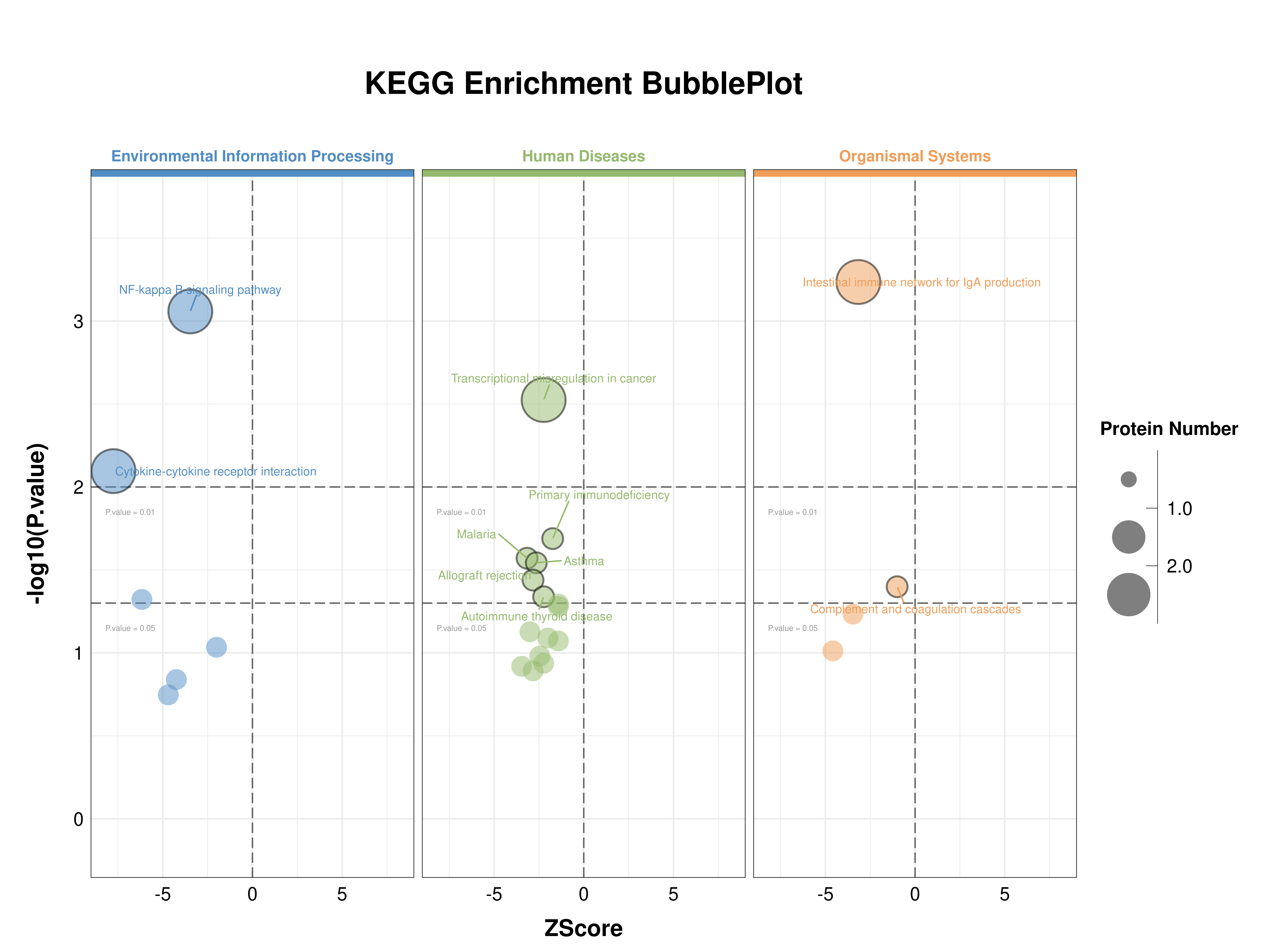

Supplement: Supplementary file 1 [file DataSheet1.zip › summary of proteomics/summary/04.Diff_analysis/COND1/FHVSZH/Enrichment/KEGG/result.FHVSZH.KEGG_ZScore_BubblePlot.P.png]

# Top 20 of Reactome Enrichment

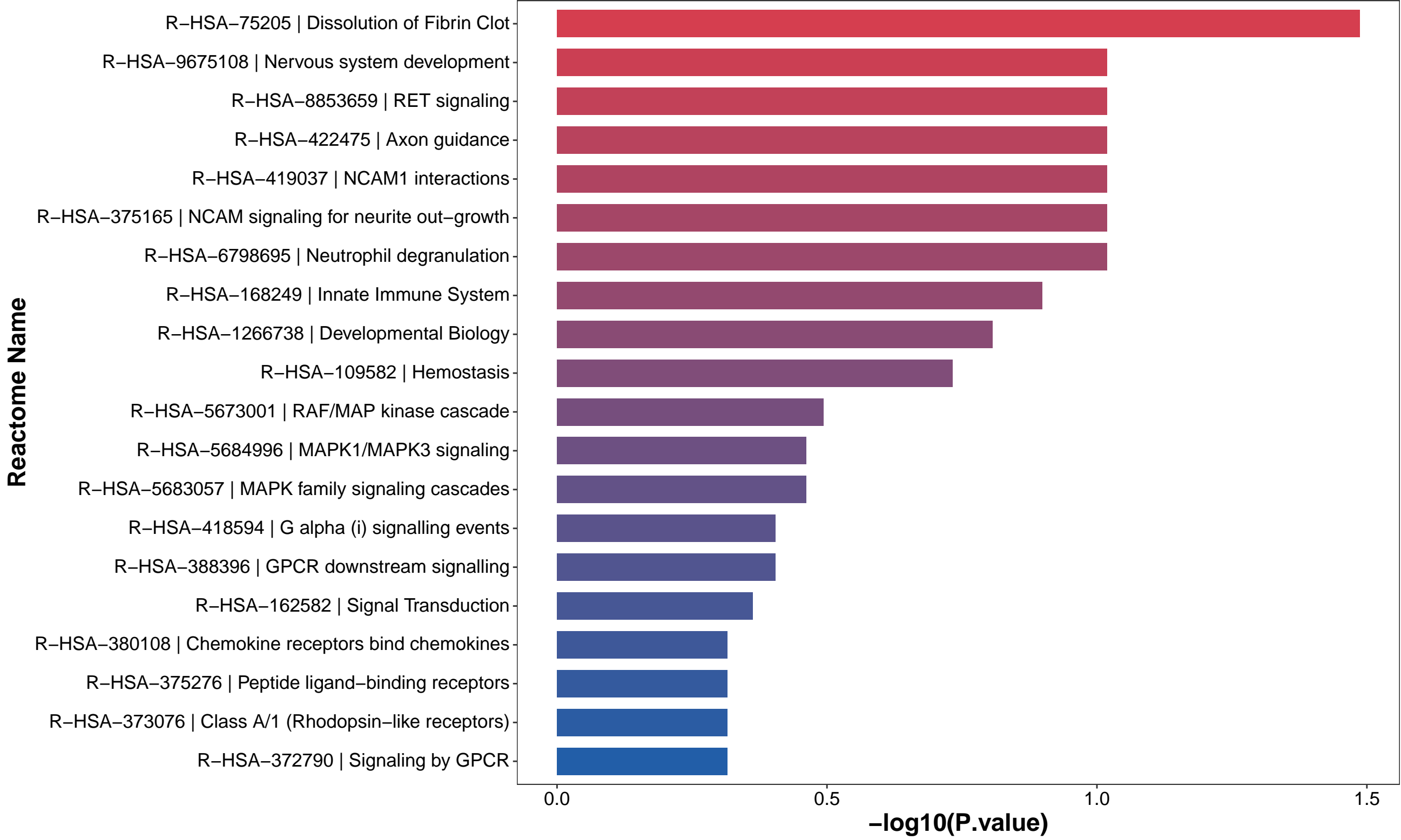

Supplement: Supplementary file 1 [file DataSheet1.zip › summary of proteomics/summary/04.Diff_analysis/COND1/FHVSZH/Enrichment/REACTOME/COND1.FHVSZH.Reactome_Enrichment.P.pdf]

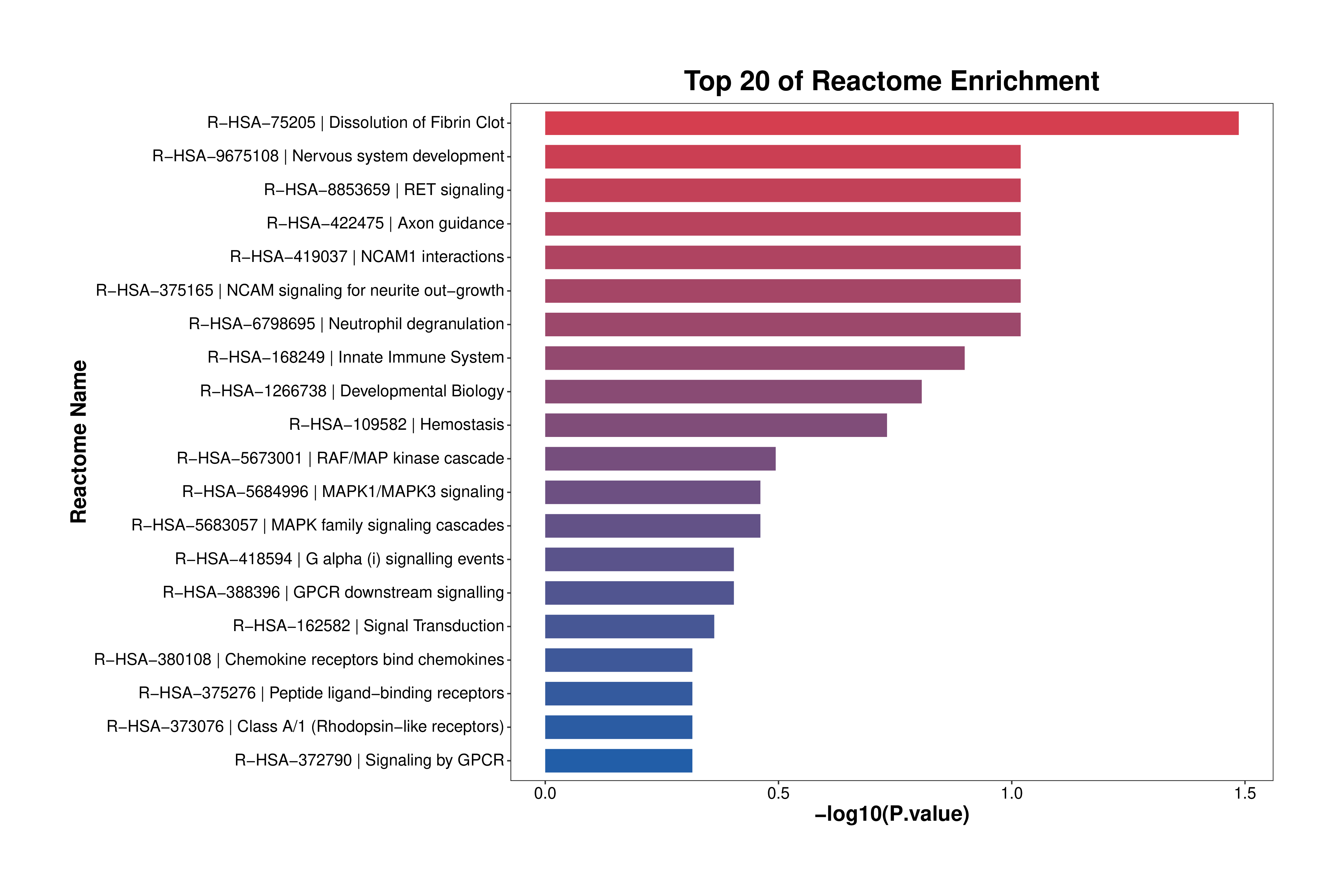

Supplement: Supplementary file 1 [file DataSheet1.zip › summary of proteomics/summary/04.Diff_analysis/COND1/FHVSZH/Enrichment/REACTOME/COND1.FHVSZH.Reactome_Enrichment.P.png]

# Top 20 of Reactome Enrichment

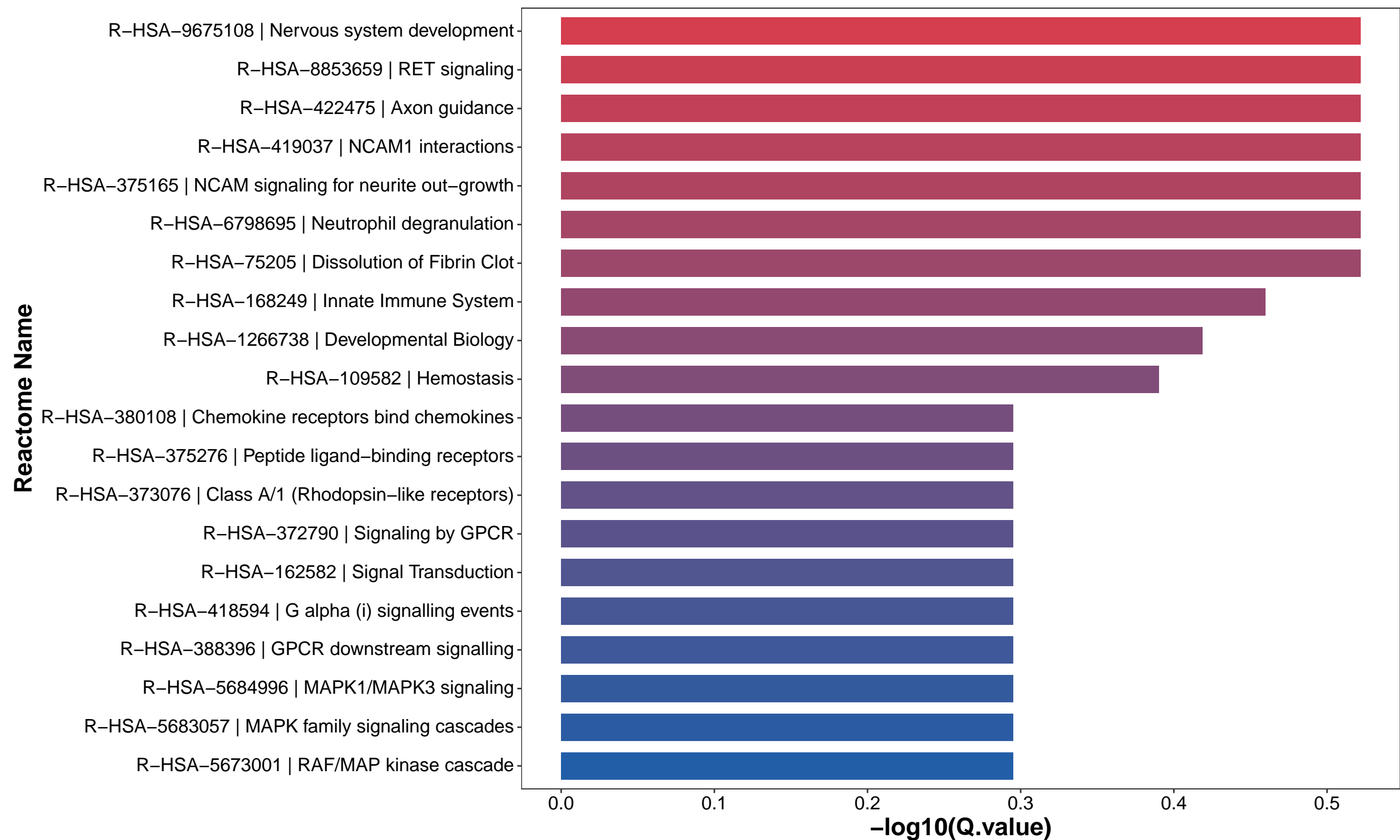

Supplement: Supplementary file 1 [file DataSheet1.zip › summary of proteomics/summary/04.Diff_analysis/COND1/FHVSZH/Enrichment/REACTOME/COND1.FHVSZH.Reactome_Enrichment.Q.pdf]

# Reactome Enrichment ScatterPlot

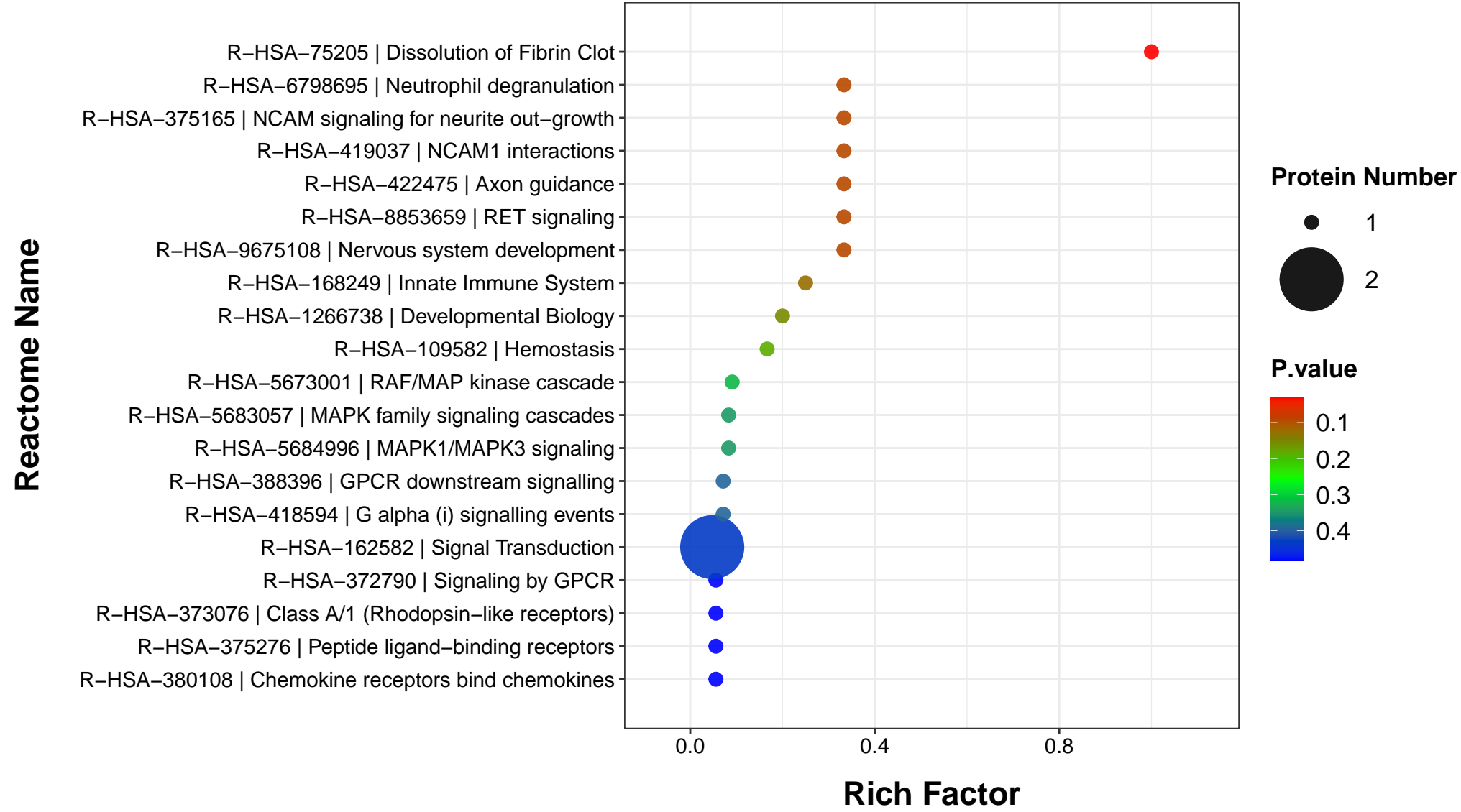

Supplement: Supplementary file 1 [file DataSheet1.zip › summary of proteomics/summary/04.Diff_analysis/COND1/FHVSZH/Enrichment/REACTOME/COND1.FHVSZH.Reactome_scatter.pdf]

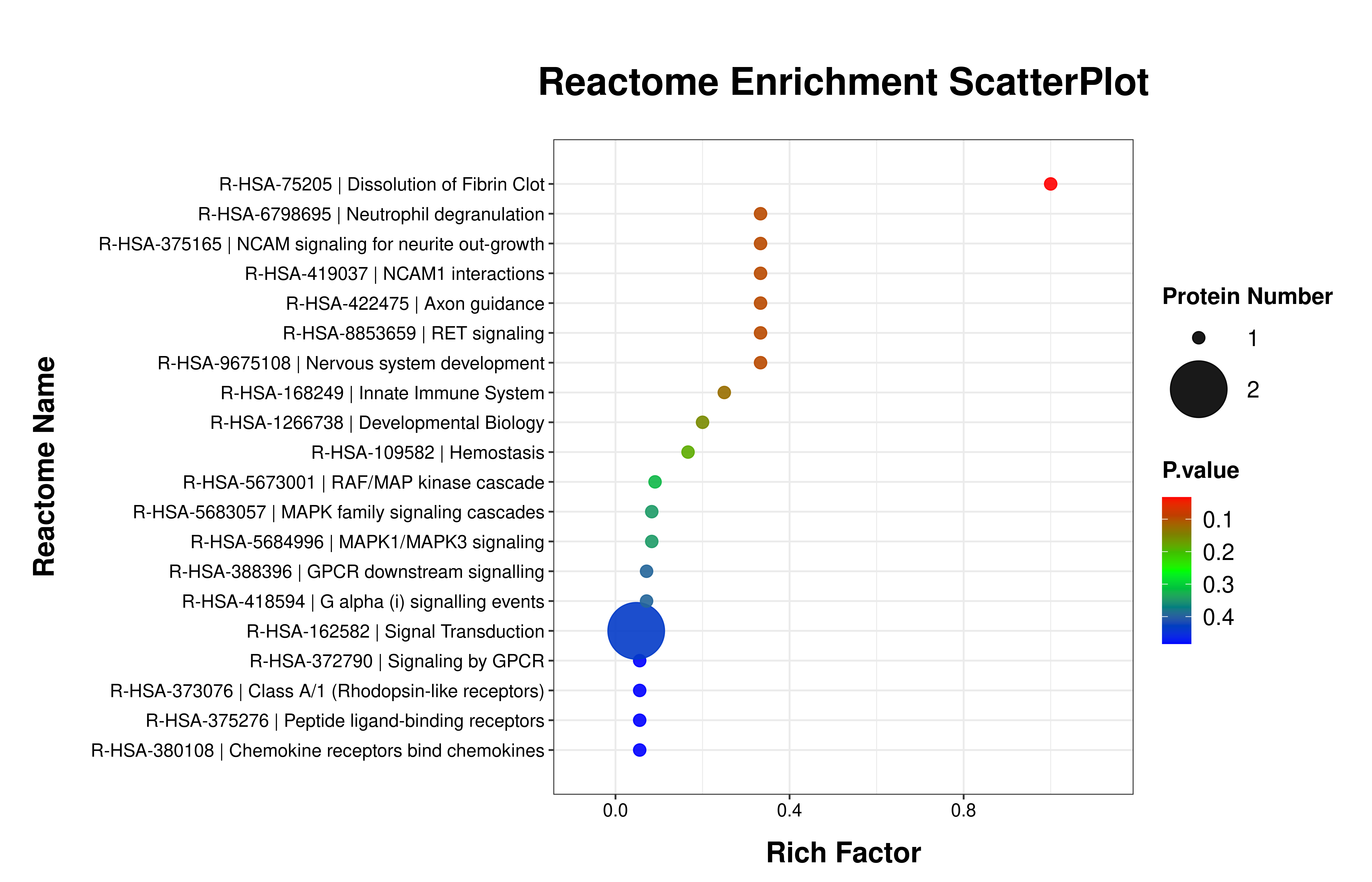

Supplement: Supplementary file 1 [file DataSheet1.zip › summary of proteomics/summary/04.Diff_analysis/COND1/FHVSZH/Enrichment/REACTOME/COND1.FHVSZH.Reactome_scatter.png]

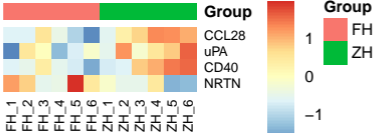

Supplement: Supplementary file 1 [file DataSheet1.zip › summary of proteomics/summary/04.Diff_analysis/COND1/FHVSZH/FHVSZH_heatmap.pdf]

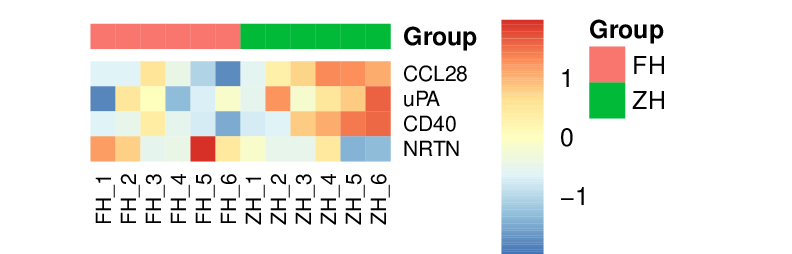

Supplement: Supplementary file 1 [file DataSheet1.zip › summary of proteomics/summary/04.Diff_analysis/COND1/FHVSZH/FHVSZH_heatmap.png]

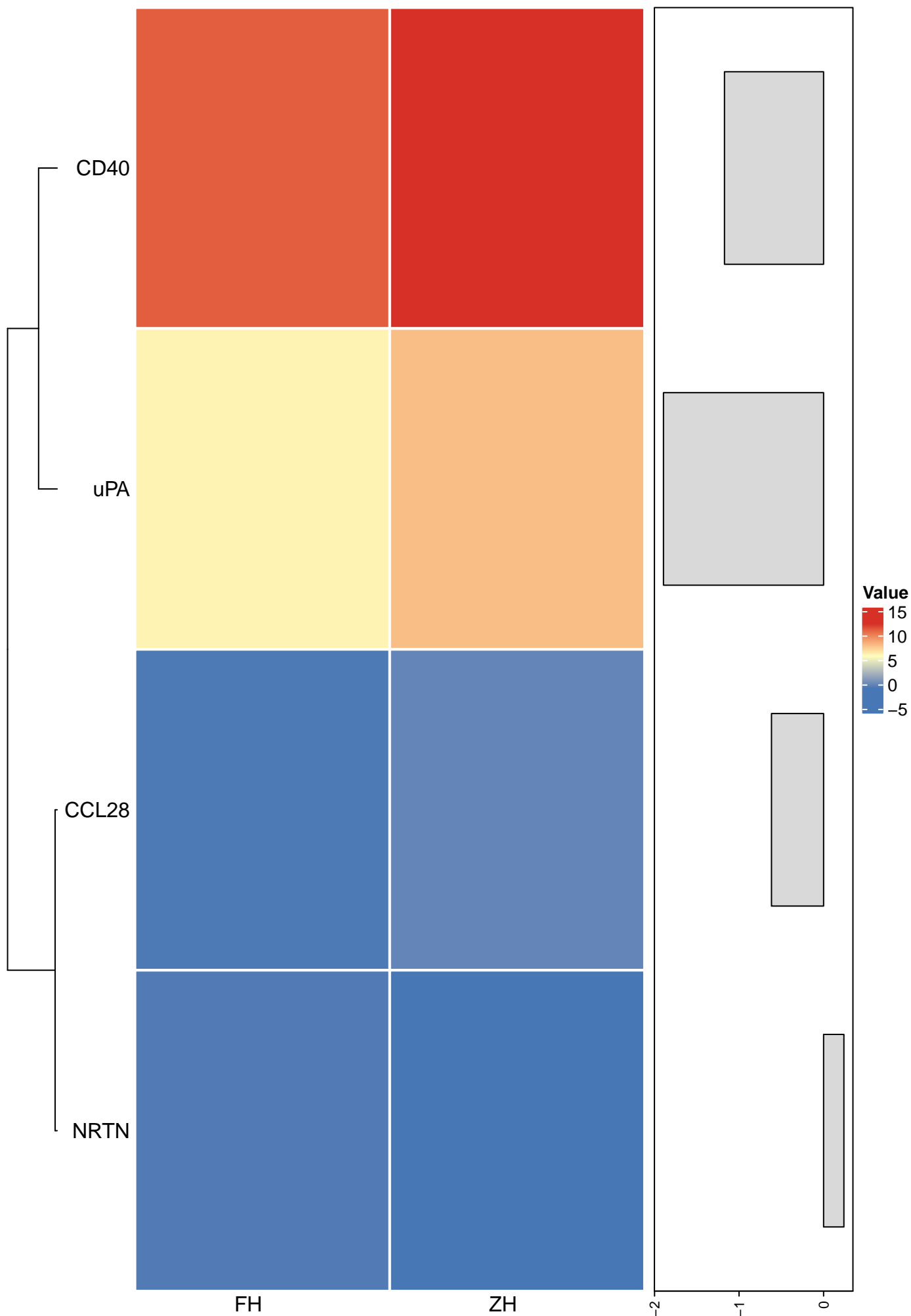

Supplement: Supplementary file 1 [file DataSheet1.zip › summary of proteomics/summary/04.Diff_analysis/COND1/FHVSZH/FHVSZH_mean_heatmap.pdf]

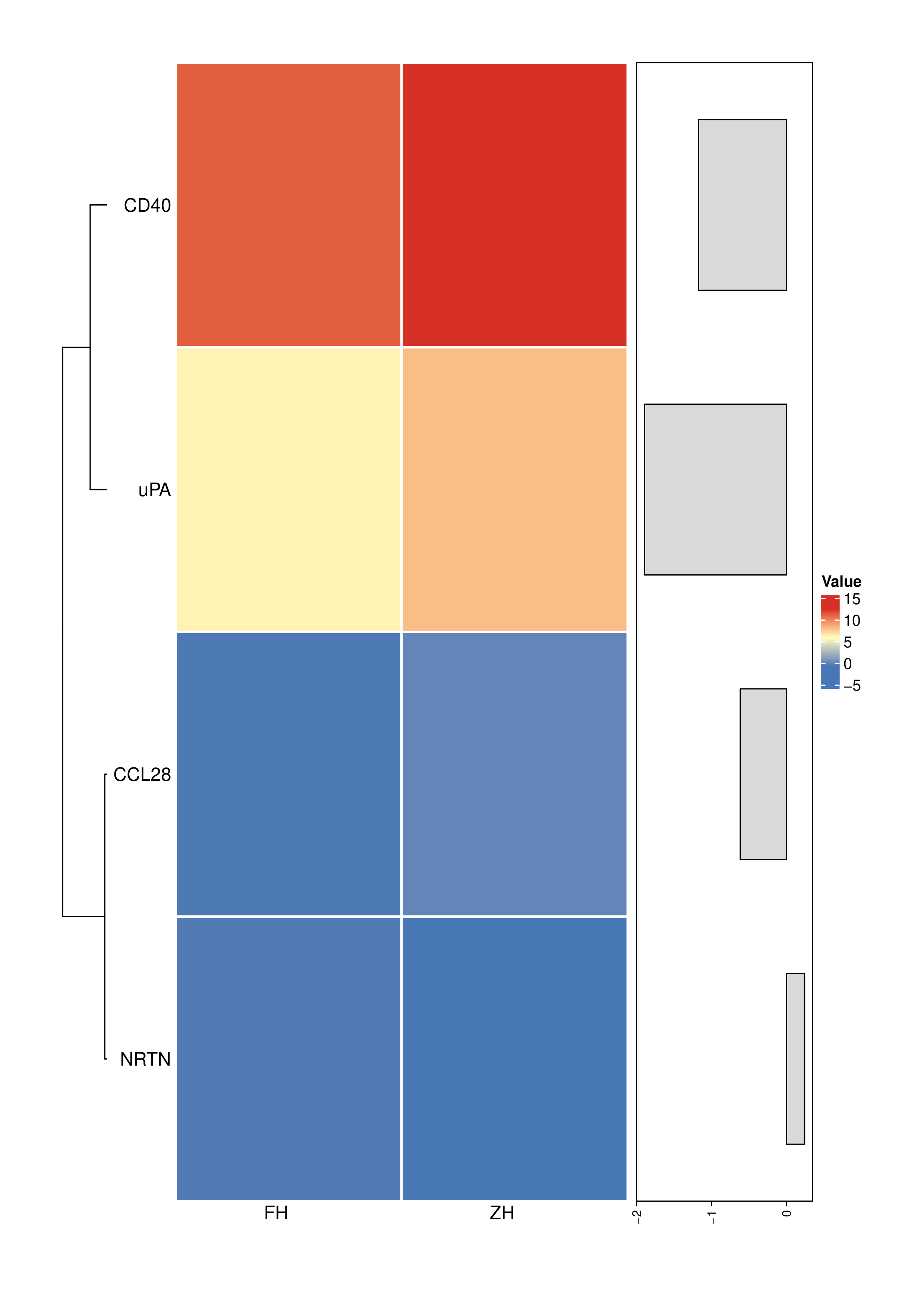

Supplement: Supplementary file 1 [file DataSheet1.zip › summary of proteomics/summary/04.Diff_analysis/COND1/FHVSZH/FHVSZH_mean_heatmap.png]

# Advanced Volcano Plot

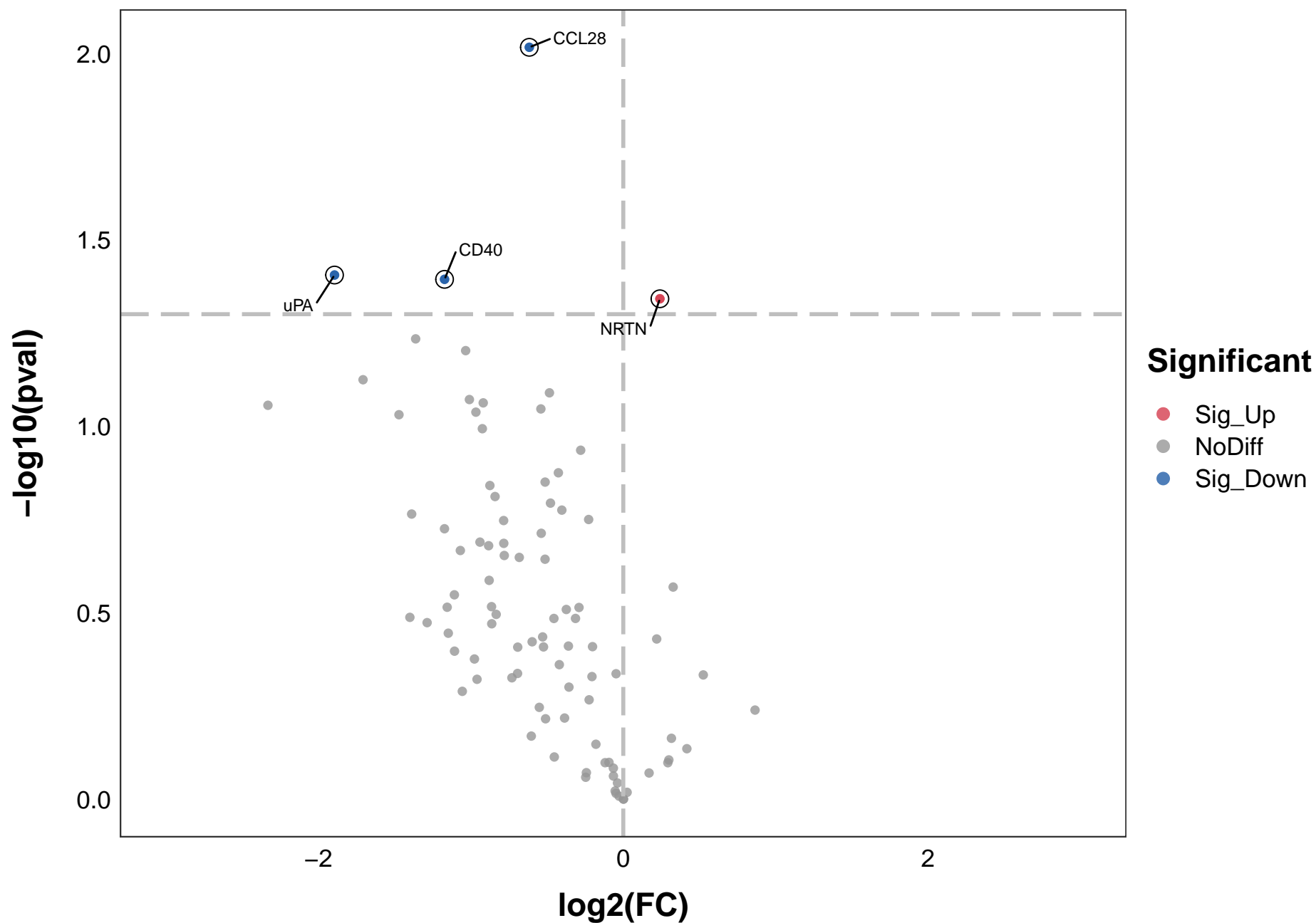

Supplement: Supplementary file 1 [file DataSheet1.zip › summary of proteomics/summary/04.Diff_analysis/COND1/FHVSZH/FHVSZH_volcano.pdf]

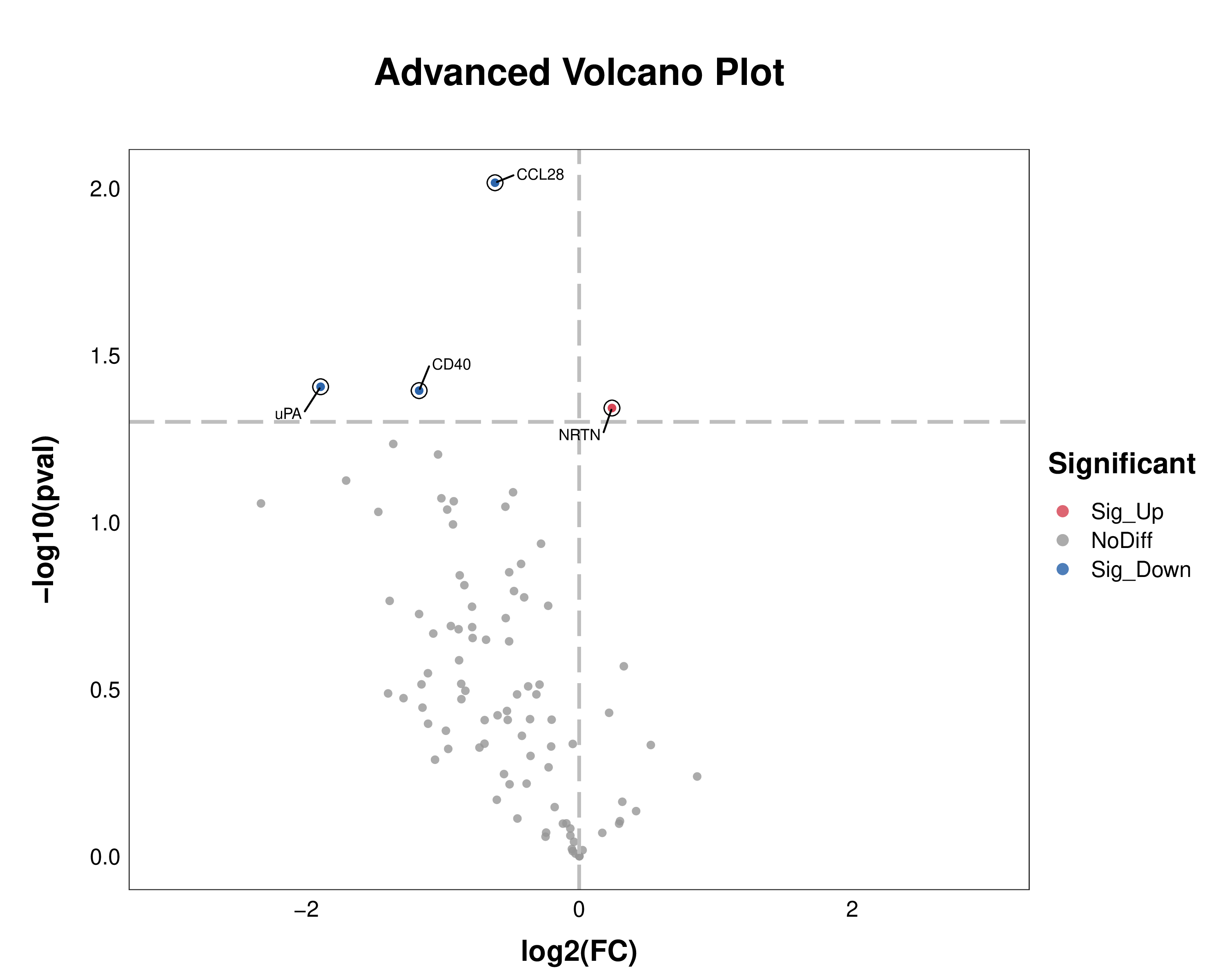

Supplement: Supplementary file 1 [file DataSheet1.zip › summary of proteomics/summary/04.Diff_analysis/COND1/FHVSZH/FHVSZH_volcano.png]

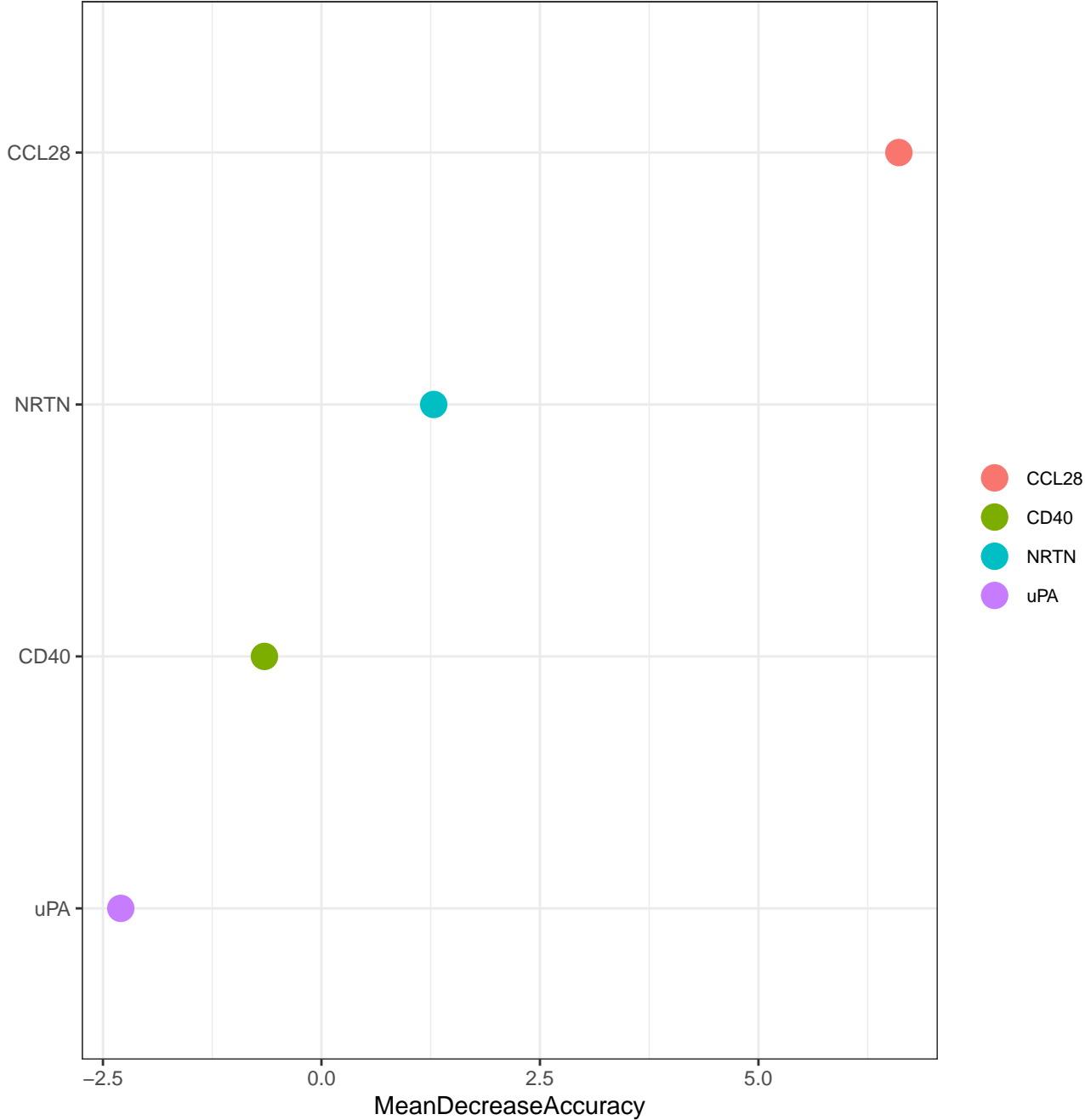

Supplement: Supplementary file 1 [file DataSheet1.zip › summary of proteomics/summary/04.Diff_analysis/COND1/FHVSZH/RandomForest/FHVSZH_randomforest_MeanDecreaseAccuracy.pdf]

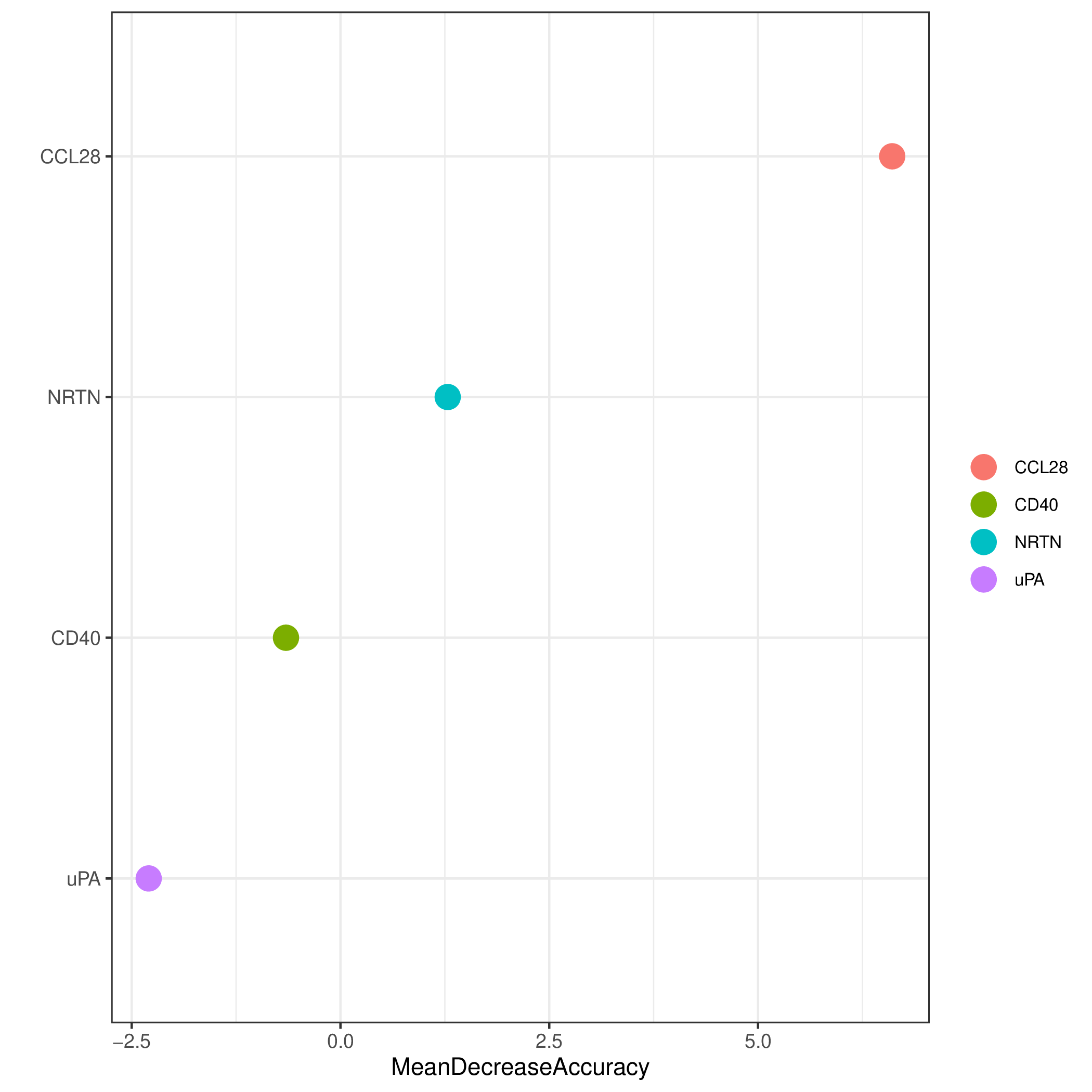

Supplement: Supplementary file 1 [file DataSheet1.zip › summary of proteomics/summary/04.Diff_analysis/COND1/FHVSZH/RandomForest/FHVSZH_randomforest_MeanDecreaseAccuracy.png]

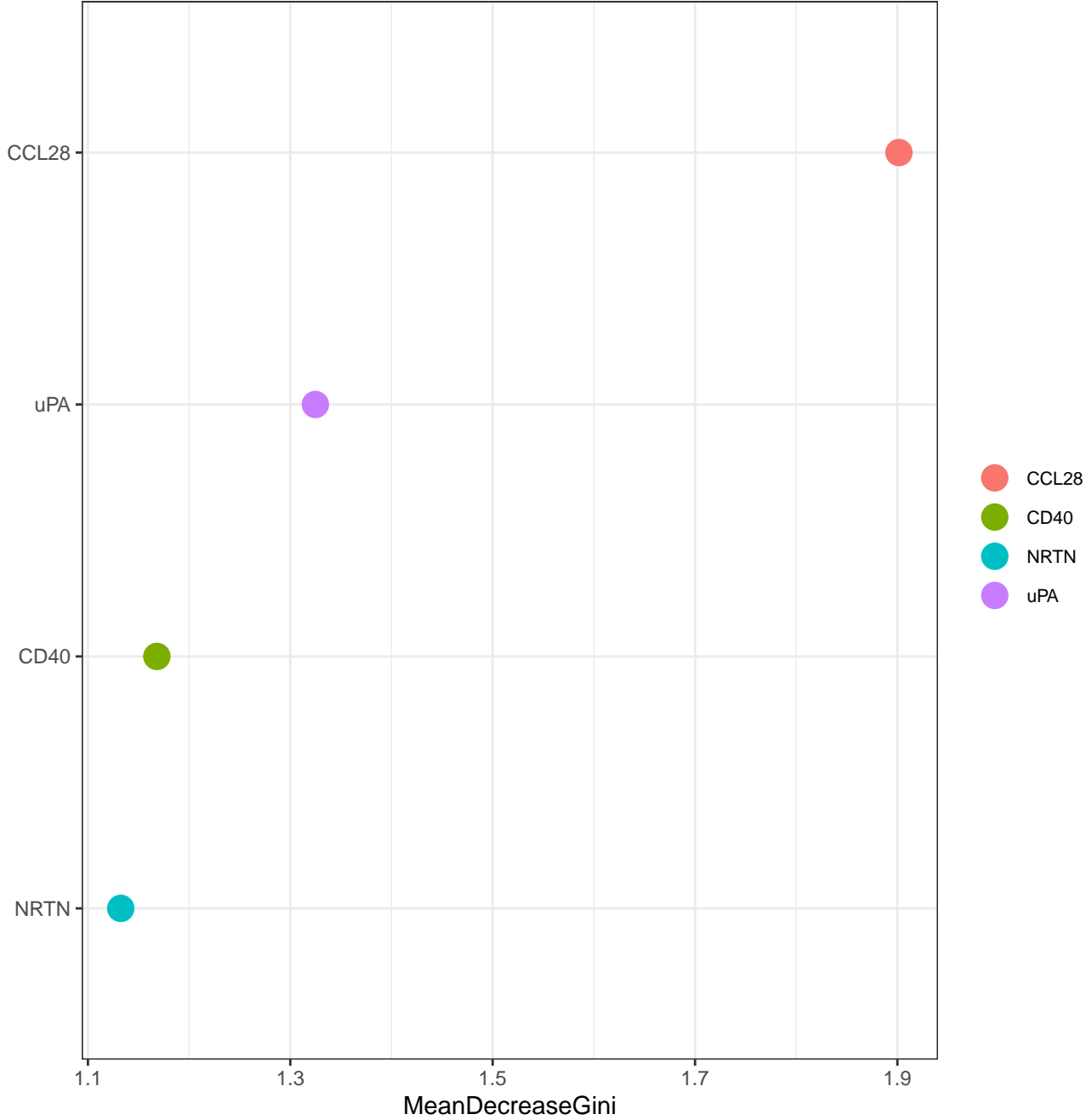

Supplement: Supplementary file 1 [file DataSheet1.zip › summary of proteomics/summary/04.Diff_analysis/COND1/FHVSZH/RandomForest/FHVSZH_randomforest_MeanDecreaseGini.pdf]

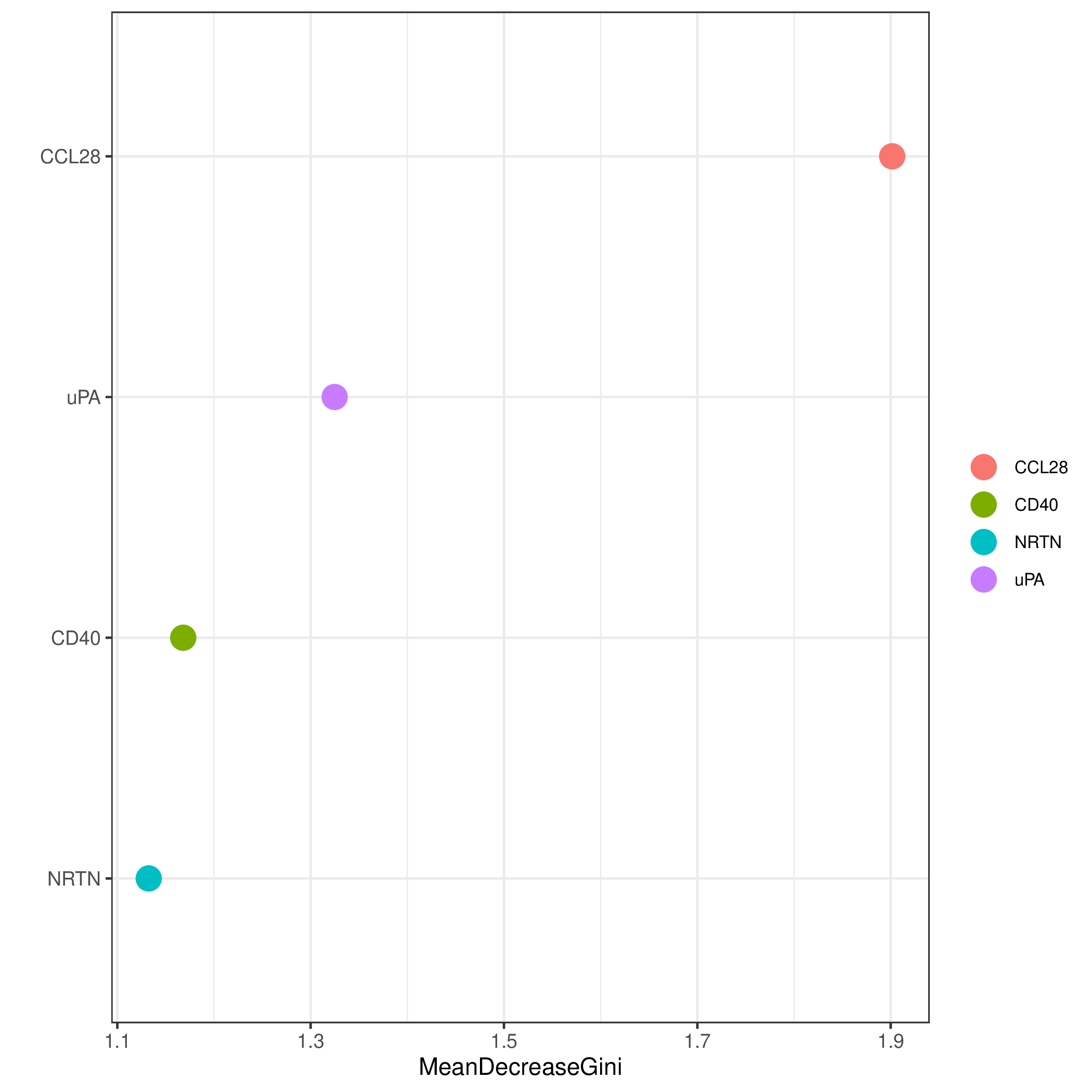

Supplement: Supplementary file 1 [file DataSheet1.zip › summary of proteomics/summary/04.Diff_analysis/COND1/FHVSZH/RandomForest/FHVSZH_randomforest_MeanDecreaseGini.png]

**ROC curve for Logistic Regression model**

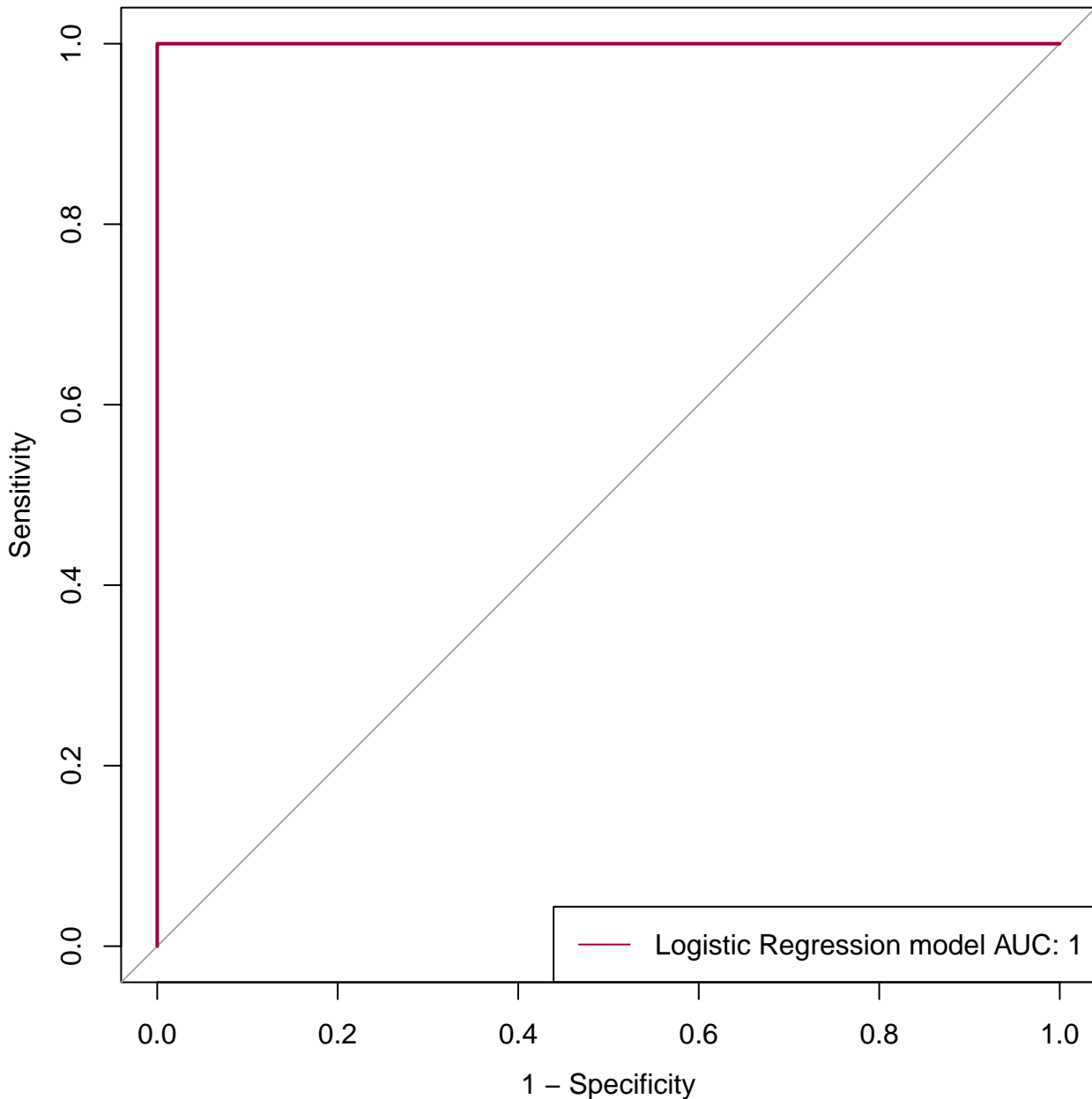

Supplement: Supplementary file 1 [file DataSheet1.zip › summary of proteomics/summary/04.Diff_analysis/COND1/FHVSZH/ROC_logistic.pdf]

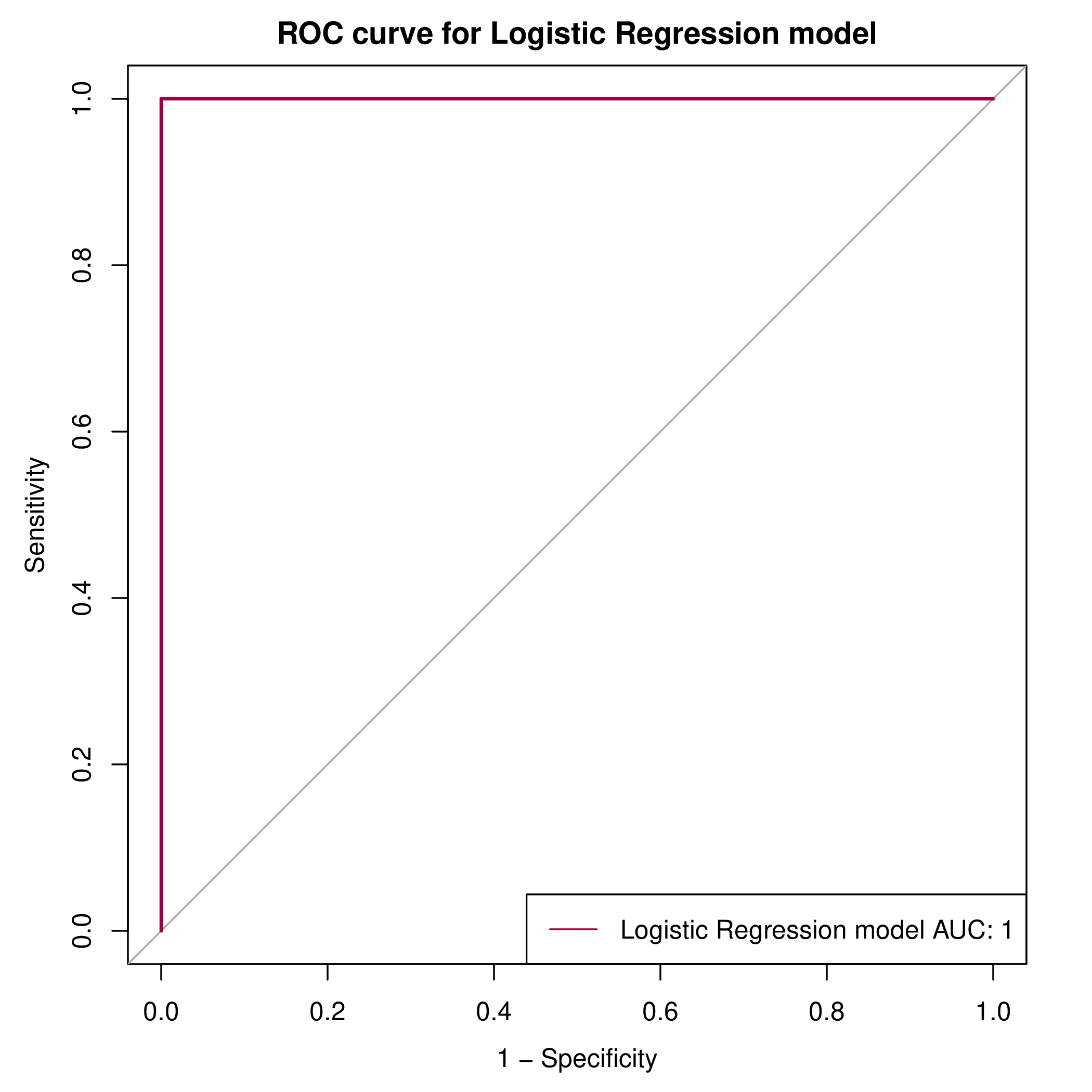

Supplement: Supplementary file 1 [file DataSheet1.zip › summary of proteomics/summary/04.Diff_analysis/COND1/FHVSZH/ROC_logistic.png]

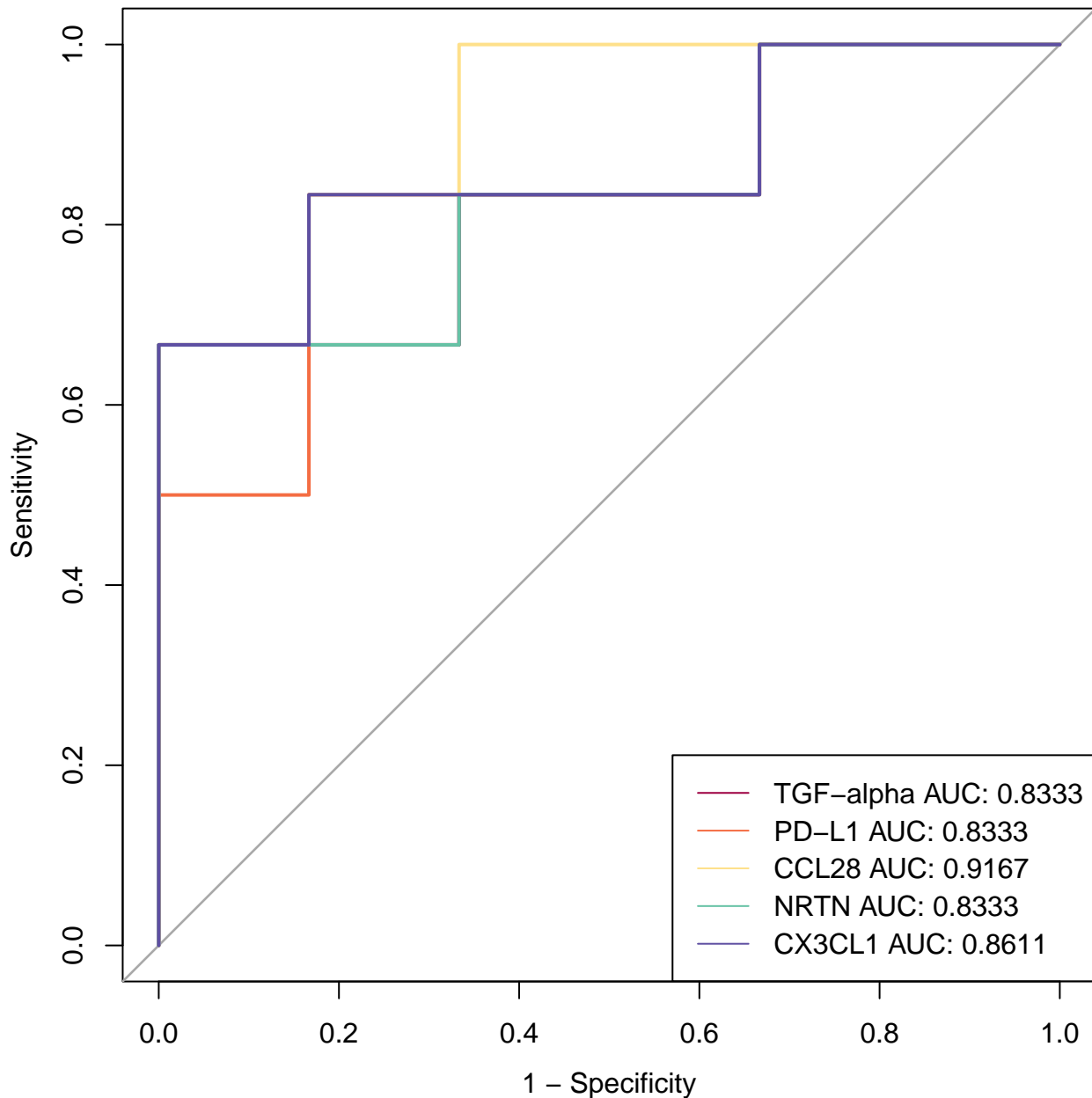

Supplement: Supplementary file 1 [file DataSheet1.zip › summary of proteomics/summary/04.Diff_analysis/COND1/FHVSZH/ROC_top5.pdf]

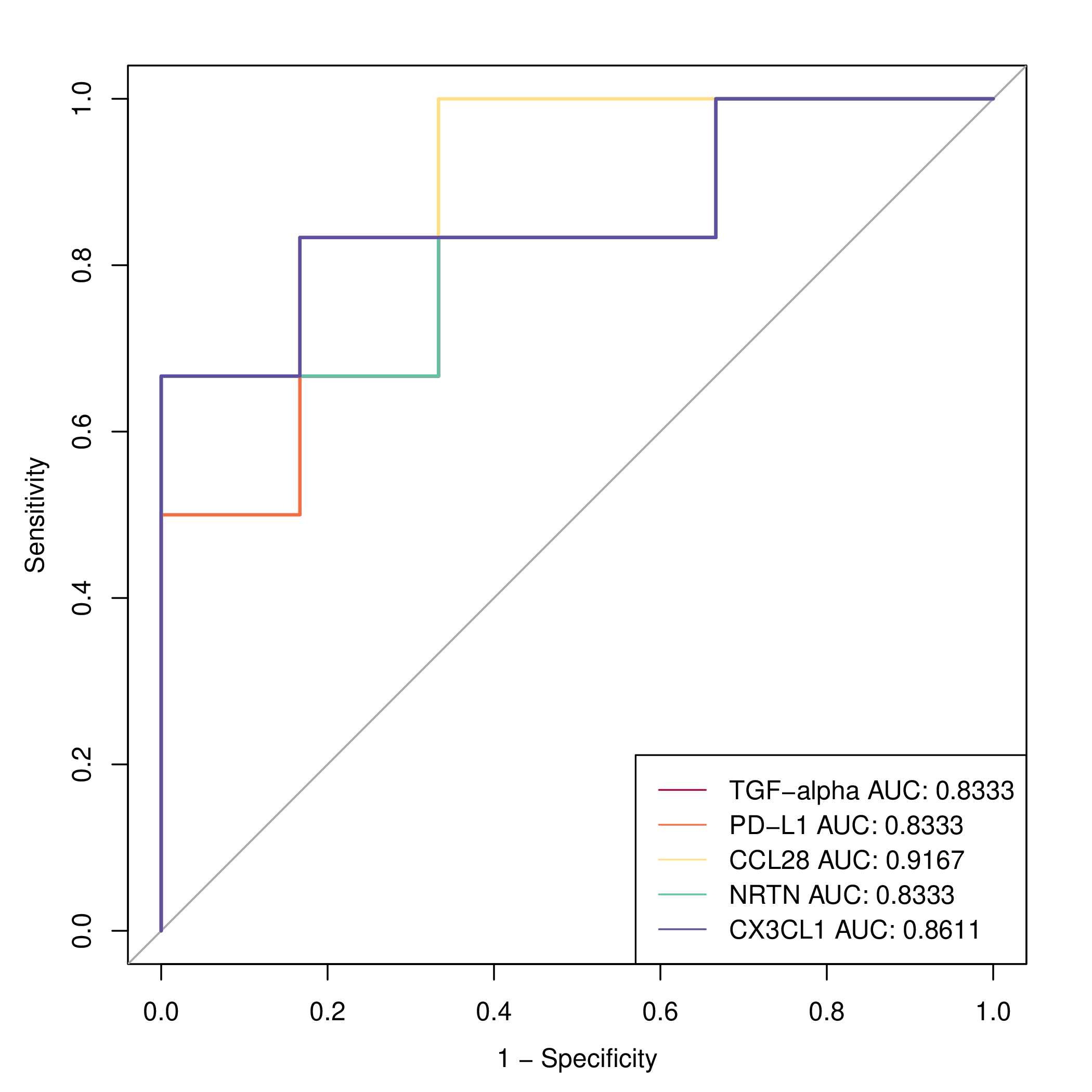

Supplement: Supplementary file 1 [file DataSheet1.zip › summary of proteomics/summary/04.Diff_analysis/COND1/FHVSZH/ROC_top5.png]

# All Significant Proteins' Expression

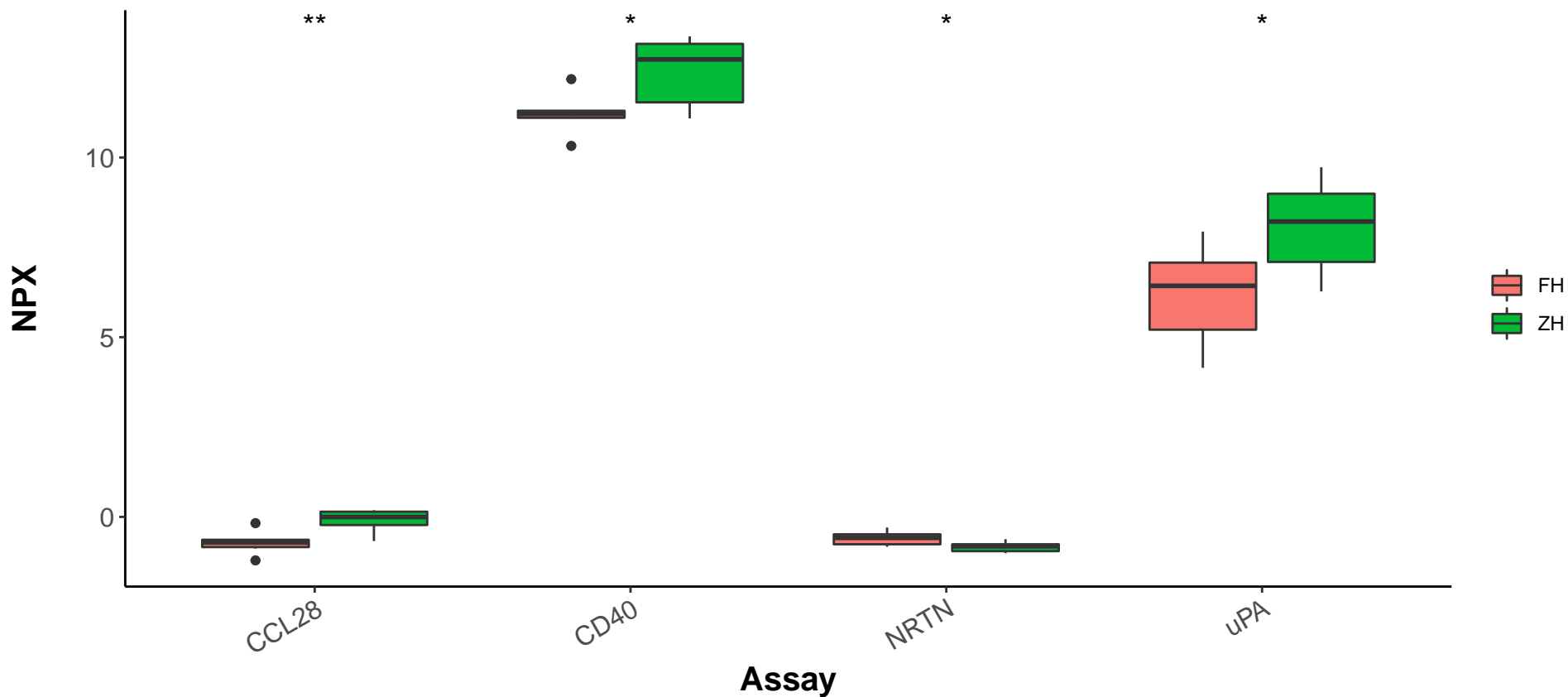

Supplement: Supplementary file 1 [file DataSheet1.zip › summary of proteomics/summary/04.Diff_analysis/COND1/FHVSZH/Top_boxplot/All_FHVSZH_Significant_Pro.pdf]

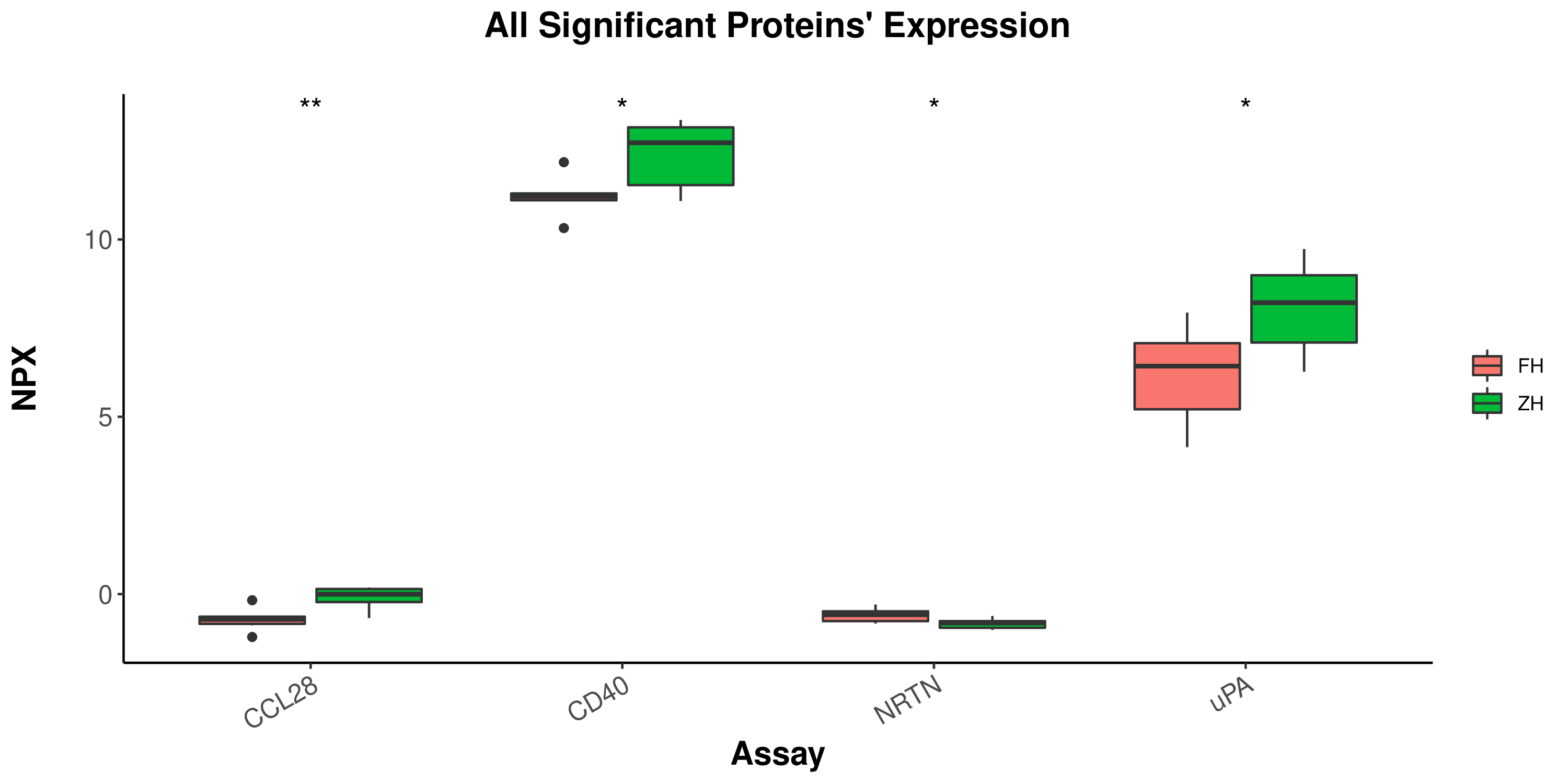

Supplement: Supplementary file 1 [file DataSheet1.zip › summary of proteomics/summary/04.Diff_analysis/COND1/FHVSZH/Top_boxplot/All_FHVSZH_Significant_Pro.png]

# CCL28 Expression Level

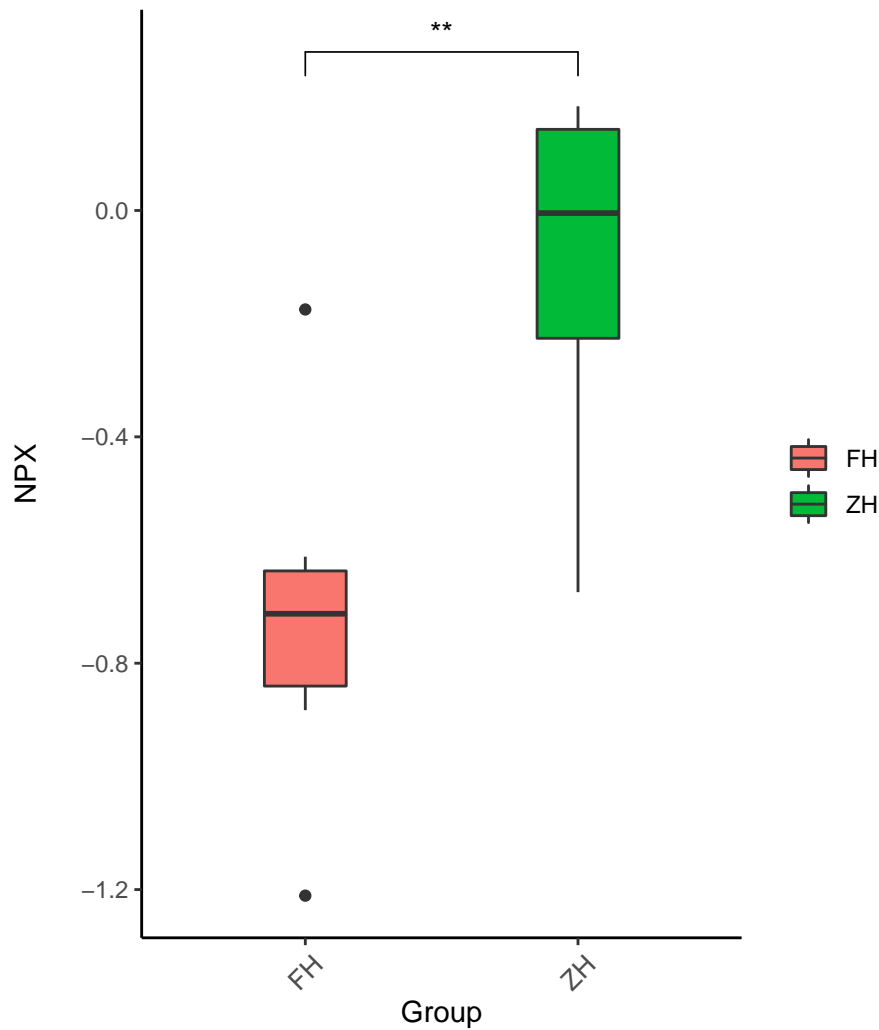

Supplement: Supplementary file 1 [file DataSheet1.zip › summary of proteomics/summary/04.Diff_analysis/COND1/FHVSZH/Top_boxplot/CCL28_boxplot.pdf]

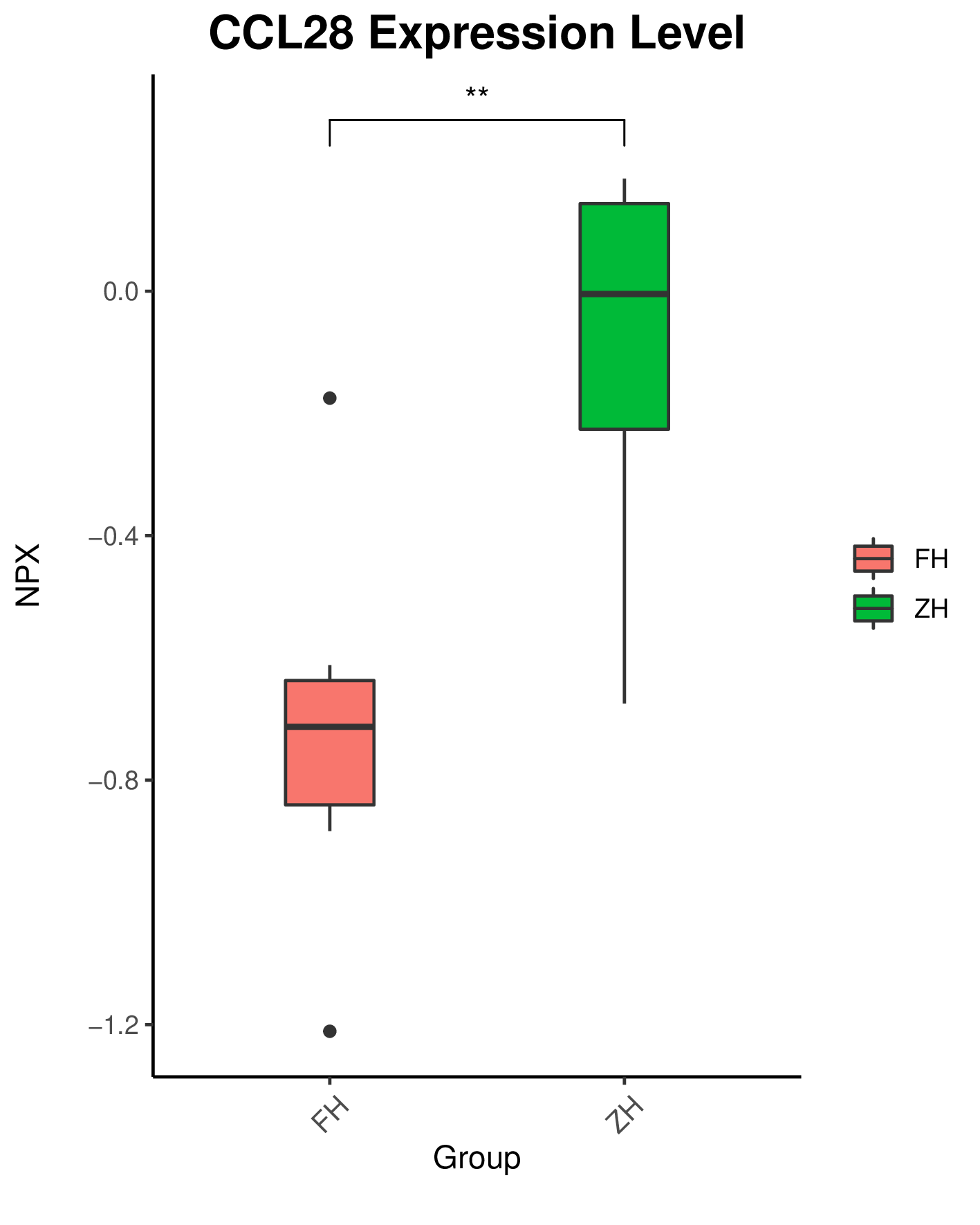

Supplement: Supplementary file 1 [file DataSheet1.zip › summary of proteomics/summary/04.Diff_analysis/COND1/FHVSZH/Top_boxplot/CCL28_boxplot.png]

# CD40 Expression Level

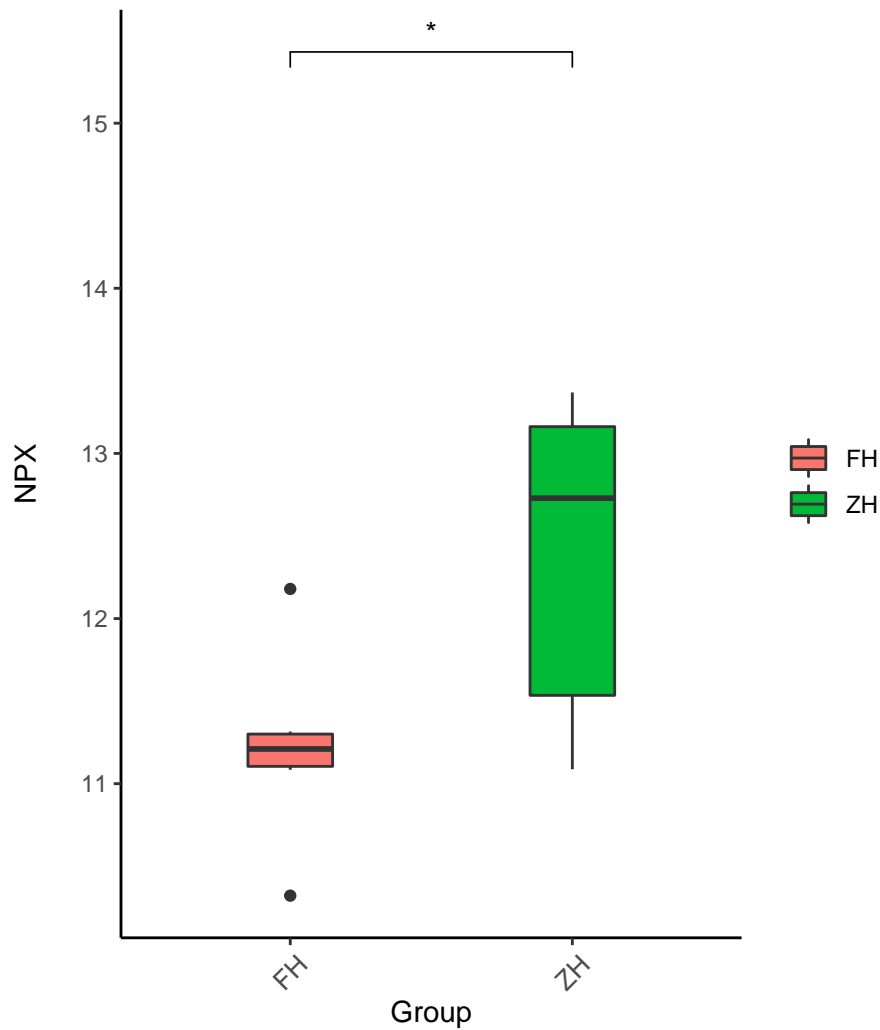

Supplement: Supplementary file 1 [file DataSheet1.zip › summary of proteomics/summary/04.Diff_analysis/COND1/FHVSZH/Top_boxplot/CD40_boxplot.pdf]

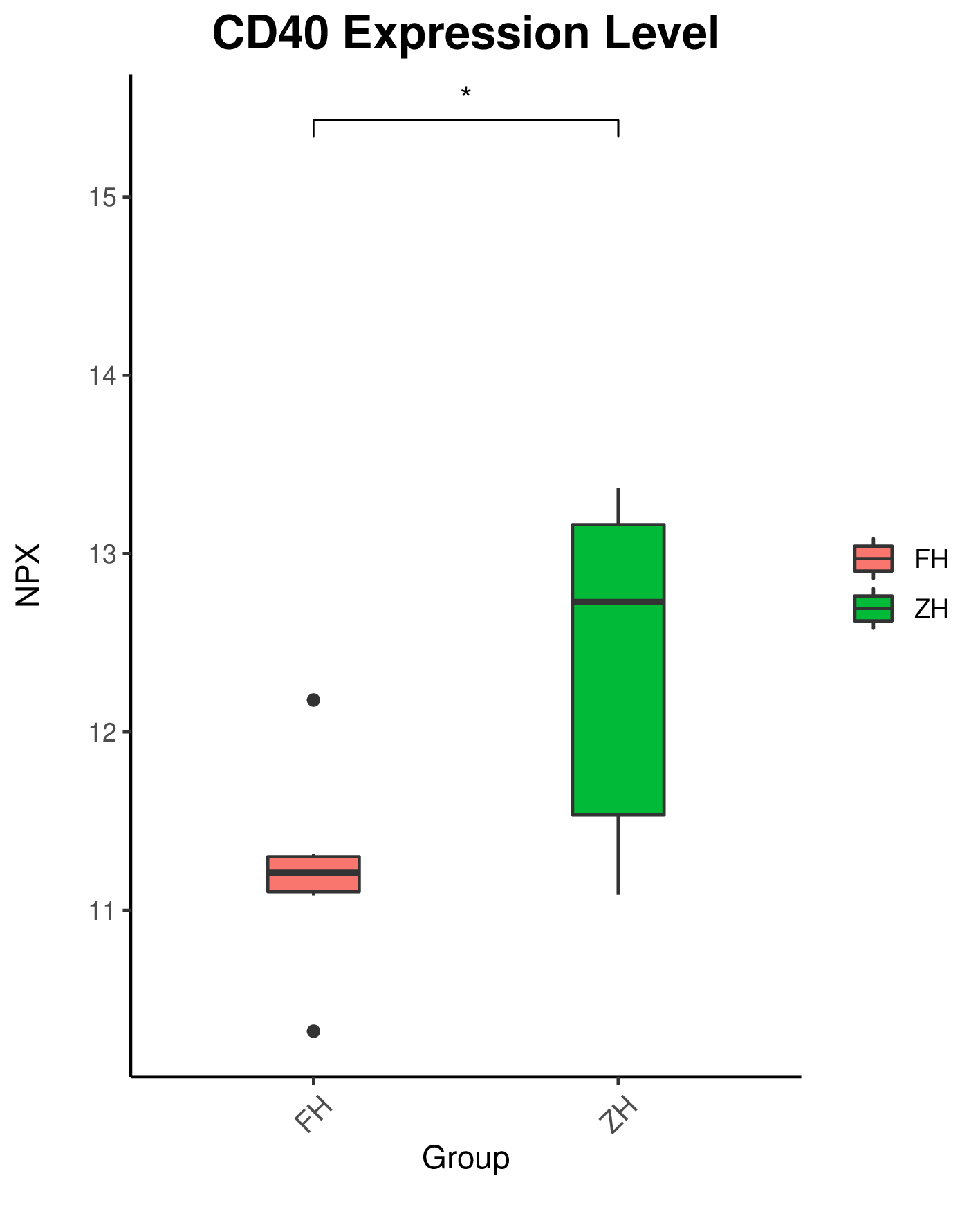

Supplement: Supplementary file 1 [file DataSheet1.zip › summary of proteomics/summary/04.Diff_analysis/COND1/FHVSZH/Top_boxplot/CD40_boxplot.png]

# NRTN Expression Level

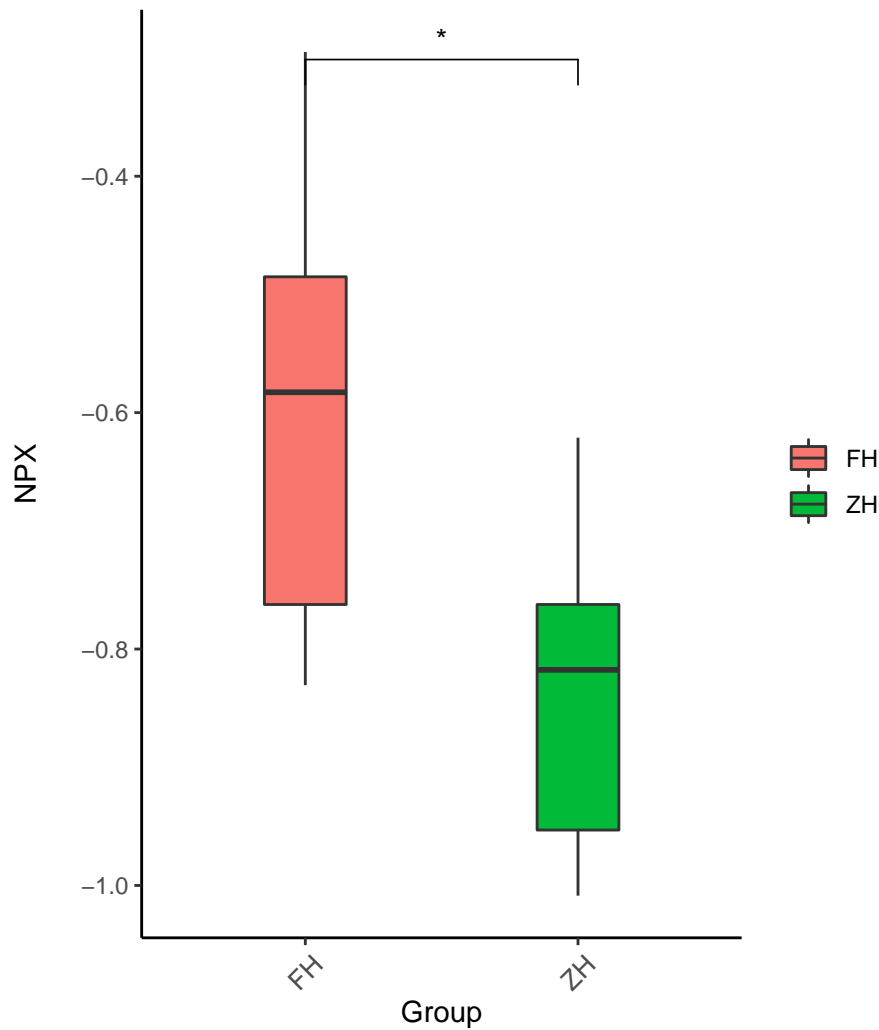

Supplement: Supplementary file 1 [file DataSheet1.zip › summary of proteomics/summary/04.Diff_analysis/COND1/FHVSZH/Top_boxplot/NRTN_boxplot.pdf]

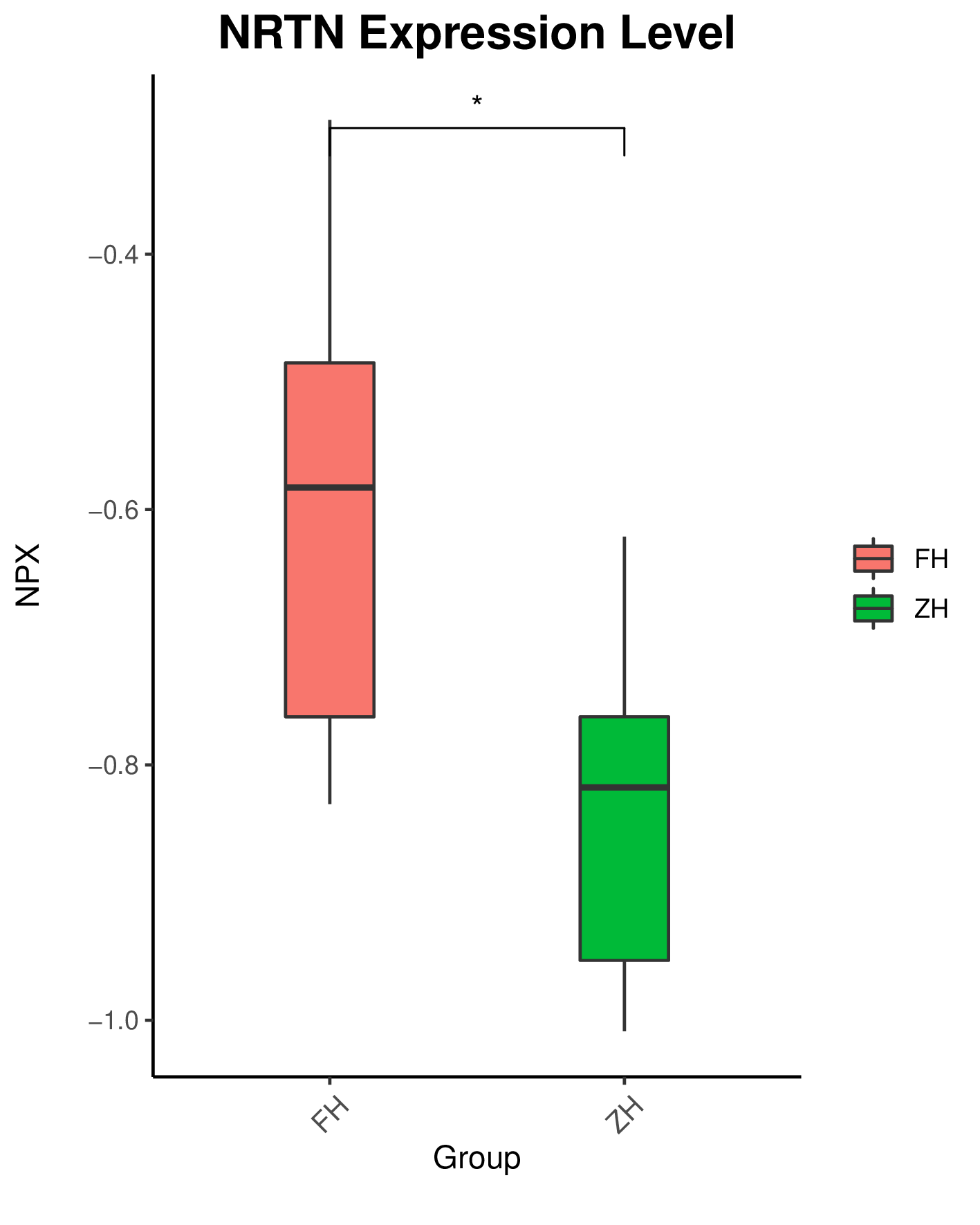

Supplement: Supplementary file 1 [file DataSheet1.zip › summary of proteomics/summary/04.Diff_analysis/COND1/FHVSZH/Top_boxplot/NRTN_boxplot.png]

# uPA Expression Level

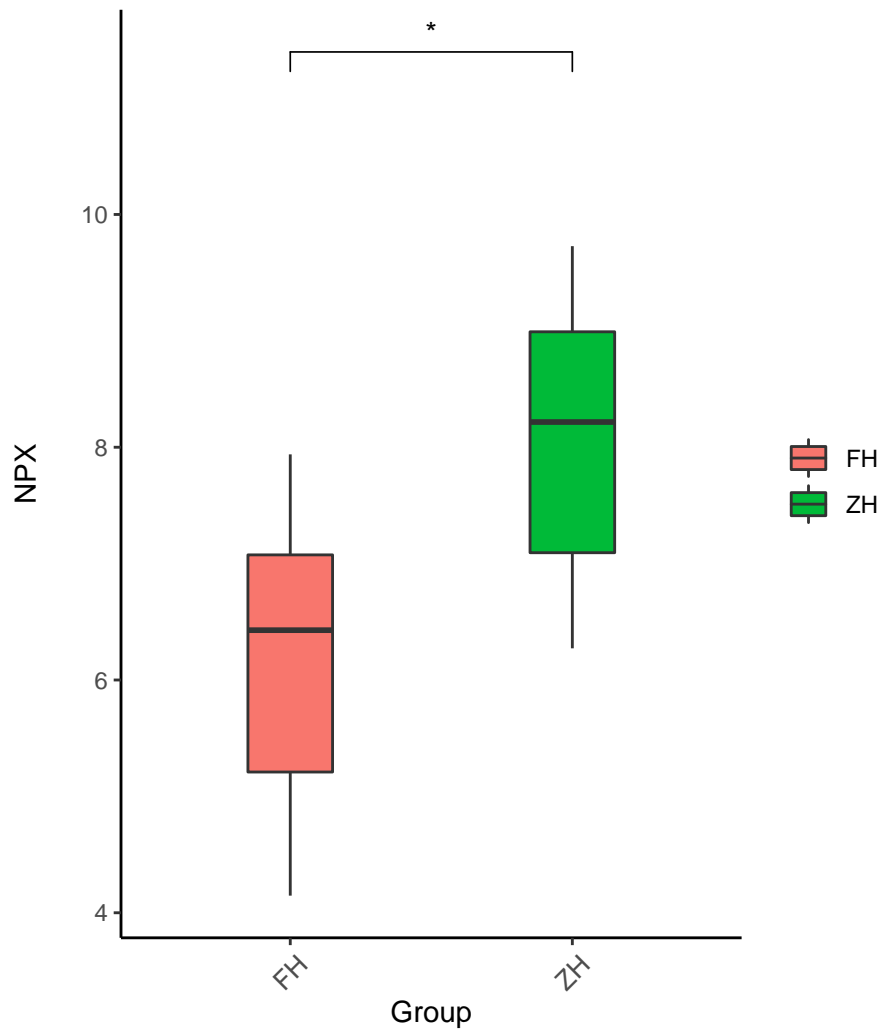

Supplement: Supplementary file 1 [file DataSheet1.zip › summary of proteomics/summary/04.Diff_analysis/COND1/FHVSZH/Top_boxplot/uPA_boxplot.pdf]

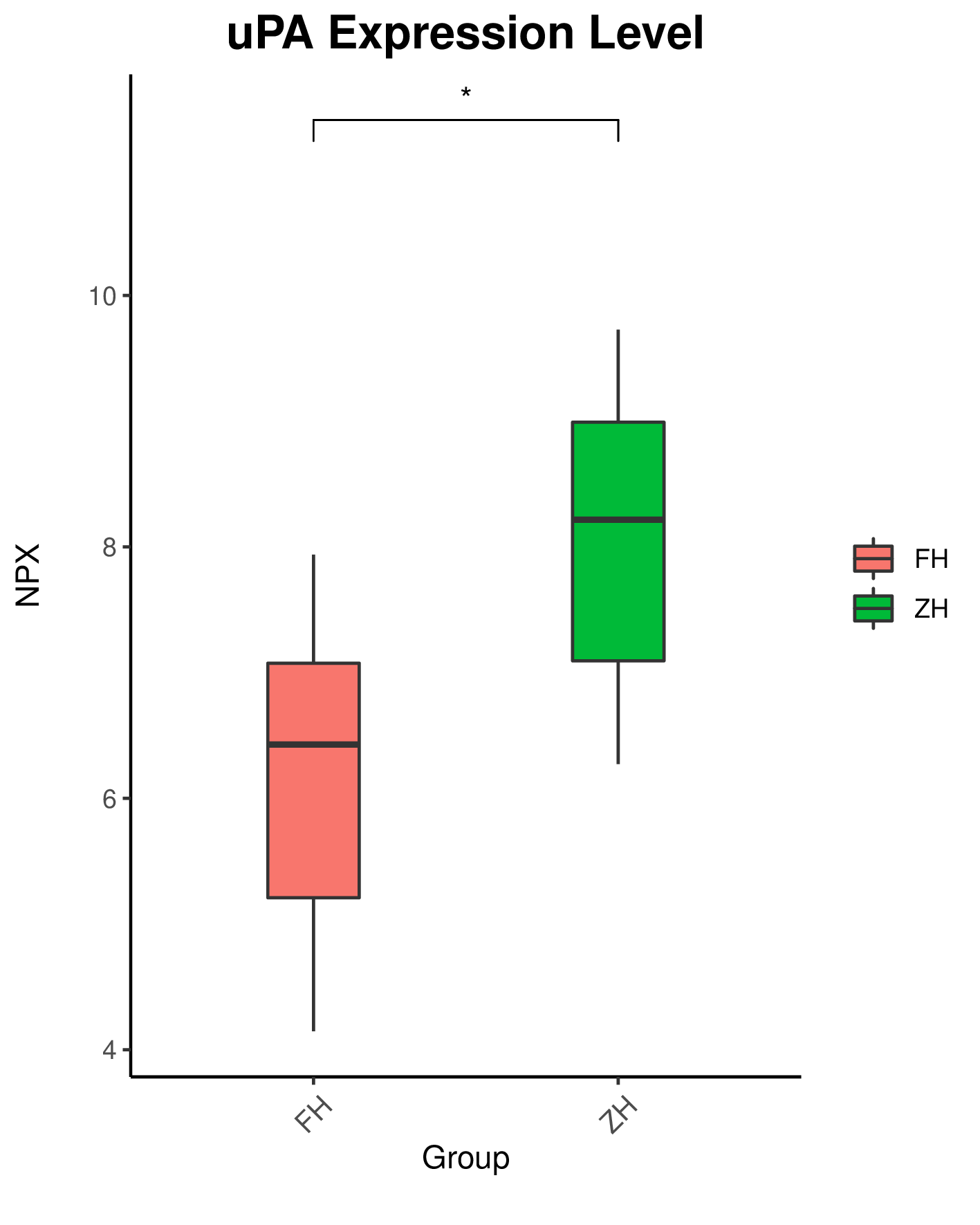

Supplement: Supplementary file 1 [file DataSheet1.zip › summary of proteomics/summary/04.Diff_analysis/COND1/FHVSZH/Top_boxplot/uPA_boxplot.png]

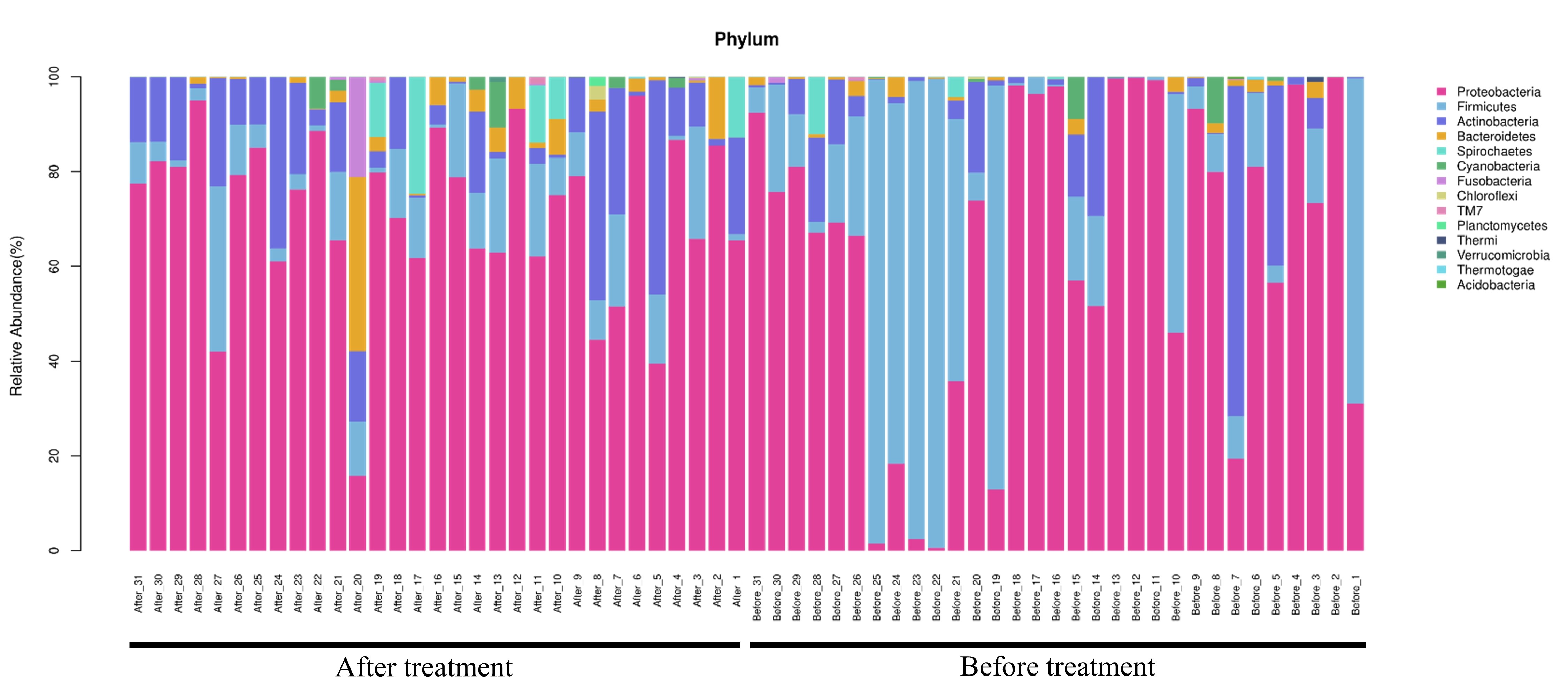

Supplement: Supplementary file 2 [file Image1.jpeg]

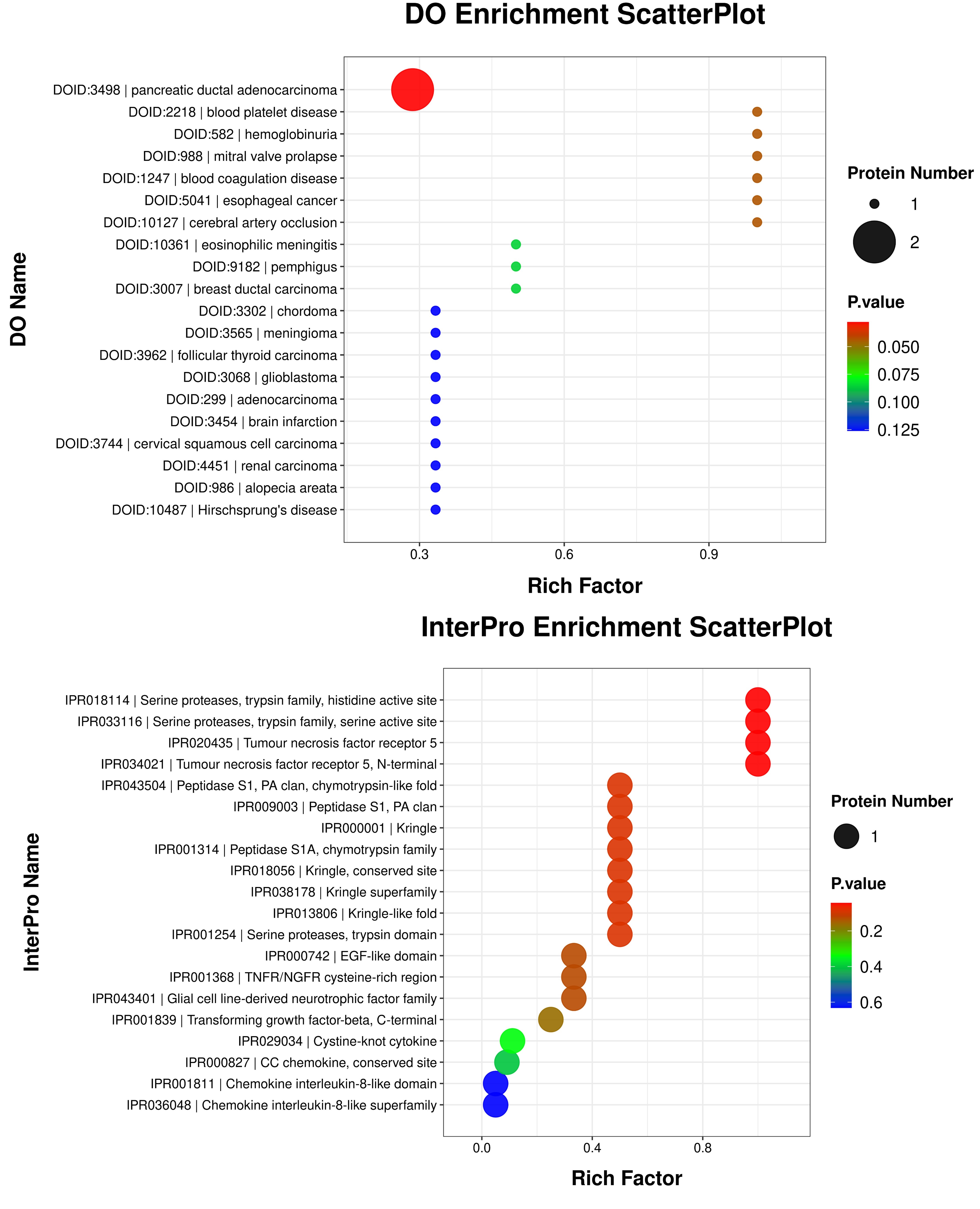

Supplement: Supplementary file 3 [file Image2.jpeg]
